# Reinforcing Oxygen Activation of Spinel Oxide via Mn─O Covalency Engineering for VOCs Oxidation

**DOI:** 10.1002/advs.75213

**Published:** 2026-04-10

**Authors:** Gan Li, Ruochen Zhou, Qianqian Chen, Guobo Li, Xiang Tu, Fengbo Yu, Wenming Liu, Jian Ji, Honggen Peng

**Affiliations:** ^1^ School of Resources and Environment Nanchang University Nanchang Jiangxi China; ^2^ Jiangxi Provincial Institute of Eco‐Environmental Science Research and Planning Nanchang Jiangxi China; ^3^ School of Chemistry and Chemical Engineering Nanchang University Nanchang Jiangxi China

**Keywords:** catalytic oxidation, covalency, oxygen activation, spinel oxide, VOCs

## Abstract

Removing VOCs efficiently demands catalysts that activate oxygen at low temperatures. Here, Mn─O covalency in MnCo spinel is strategically modulated through an in situ hard‐templating method to incorporate Si and generate active oxygen species. Si incorporation induces Mn─O bond elongation and charge redistribution, weakening Mn─O covalency and forming Mn^4+^─O─Co^3+^ centers that promote dual activation of molecular and lattice oxygen. The optimized catalyst achieves T_90_ of 168, 226, and 260°C for ethyl acetate, toluene, and propane, respectively, with excellent water resistance and long‐term stability (100 h) for ethyl acetate oxidation. Combined in situ spectroscopy (DRIFTS and EXAFS) studies and DFT calculations reveal that weakened Mn─O covalency can accelerate the rate‐limiting step of acetate oxidation to boost performance. This strategy can also be extended to synthesize MnO_x_, Co_3_O_4_, and MnCeO_x_ for efficient VOC oxidation. Our work offers a new strategy to enhance oxygen activation via metal‐oxygen covalency modulation for low‐temperature VOC abatement.

## Graphical abstract

A MnCo spinel catalyst with weakened Mn─O covalency was synthesized via a hard‐template method. The reduced Mn─O covalency facilitates localized electron redistribution, promoting the activation of both molecular oxygen and lattice oxygen, and thereby enabling the efficient and complete oxidation of VOCs (ethyl acetate, toluene, and propane).

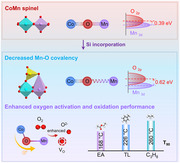

## 1 Introduction

VOCs are ubiquitous in modern industrial processes and pose significant environmental and health risks [1]. Catalytic oxidation is a promising strategy for completely converting VOCs into CO_2_ and H_2_O with low energy input [2]. Industrial VOC emissions typically consist of complex mixtures such as oxygenated VOCs and light alkanes, which contain robust chemical bonds (e.g., C═O, C─C, and C─H) [3]. The cleavage of these bonds via oxidation at low temperatures remains a major challenge due to the limited oxygen activation ability that forms reactive oxygen species (ROS) [4, 5, 6]. Therefore, enhancing the oxygen activation ability of the catalyst is critical for boosting the catalytic oxidation of VOCs [7].

Various catalysts, including noble metal and transition metal oxides, have been used for VOC oxidation. Although noble metal catalysts (Pt, Pd) exhibit exceptional catalytic performance, cost‐effective and efficient alternatives are more promising [8]. Spinel oxides (AB_2_O_4_), where A and B cations occupy tetrahedral and octahedral sites, respectively, have attracted increasing attention for oxidation reactions due to tunable compositions and valence states, flexible electronic configurations, and robust structural stability under harsh conditions [9, 10]. Although spinel oxides generally suffer from low specific surface area and thus insufficient exposure of active sites, their high tunability paves an effective way to modulate the oxygen activation process, including both molecular oxygen and lattice oxygen [11, 12, 13].

In heterogeneous catalytic oxidation of VOCs, oxygen activation involves the adsorption and dissociation of molecular oxygen, as well as dynamic migration and regeneration of lattice oxygen [14]. The activation of O_2_ involves its transformation from the stable ground state to highly reactive excited states [15, 16]. This process is fundamentally governed by degenerate π^*^_2p_ orbitals of ground‐state oxygen, which possess two unpaired electrons, enabling electron acceptance that reduces the O─O bond order, weakening or completing bond cleavage [17, 18, 19]. The key to lattice oxygen activation lies in a dynamic oxygen vacancy (OV) mediated redox cycle, where the M─O bond strength governs the barrier for O^2−^ desorption [20, 21, 22]. This process generates coordinatively unsaturated OV sites alongside electron‐enriched metal centers, which serve as active sites for O_2_ activation [23, 24]. Subsequently, dissociative chemisorption of O_2_ at these defect sites occurs via metal‐oxygen hybridization, completing the catalytic cycle through oxygen replenishment and catalyst reoxidation [25, 26]. The geometric and electronic configuration of active sites synergistically dictates the activation and transformation of oxygen species in oxidation reactions [27]. Guo et al. [28]. revealed that morphological control of NiCo_2_O_4_ spinel to expose the (311) facet enhances oxygen mobility. Ren et al. [29]. demonstrated that A‐site doping in MCo_2_O_4_ spinel tunes the Co^3+^/Co^2+^ ratio, thereby enhancing oxygen mobility for improved toluene oxidation. Liu et a.l [30]. constructed λ‐MnO_2_ on MnCo spinel via acid etching, weakening Mn─O bonds to promote lattice oxygen activation. Ye et al. [31]. constructed oxygen vacancies (OVs) through the solvothermal method to regulate catalyst oxygen mobility and surface‐reactive oxygen species, thereby enhancing toluene oxidation. Although the oxygen activation ability of spinel oxides can be modulated by facet engineering [32], metal doping [33, 34], and defect regulation [35, 36], existing strategies rely heavily on vacancy construction. From a molecular orbital perspective, the modulation of metal‐oxygen covalency is expected to be an effective approach to facilitate the activation of both molecular oxygen and lattice oxygen. Nevertheless, the mechanism underlying their simultaneous activation on spinel catalysts remains unclear.

Herein, we introduce an in situ hard‐templating strategy to synthesize Si‐incorporated MnCo spinel catalyst with tunable Mn─O covalency, enabling highly efficient oxidation of typical VOCs such as ethyl acetate (EA), toluene, and propane. A combination of structural characterizations, in situ spectroscopic studies, and DFT calculations was employed to elucidate how Si incorporation modulates Mn─O covalency and promotes the dual activation of molecular and lattice oxygen. These studies provide atomic‐scale mechanistic insights into oxygen activation and establish a correlation between catalyst structure and performance. Furthermore, by integrating in situ DRIFTS with theoretical calculations, a reaction pathway is proposed, offering fundamental understanding of how Mn─O covalency regulation in spinel oxides drives effective activation of both O_2_ and lattice oxygen for broad VOC oxidation applications.

## 2 Results and Discussion

### 2.1 Mn─O Covalency Regulation by Si Incorporation in MnCo_4_‐E

**FIGURE 1:**
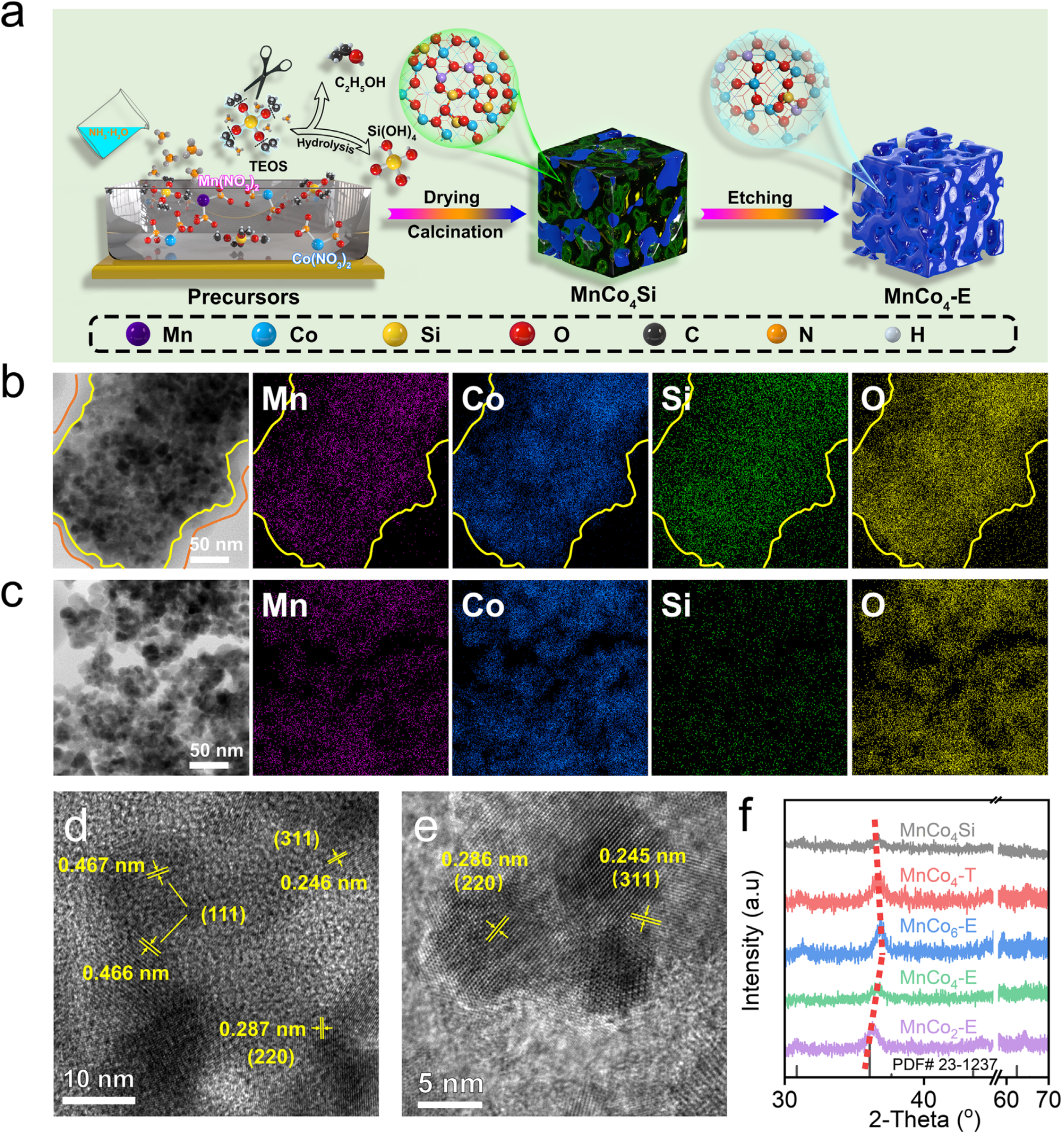
(a) Schematic illustration of the synthetic procedure of MnCo_4_‐E. TEM images and corresponding elemental mapping of (b) MnCo_4_Si and (c) MnCo_4_‐E. HRTEM images of (d) MnCo_4_‐E, and (e) MnCo_4_Si. (f) Powder XRD patterns of MnCo_4_Si, MnCo_4_‐T, and MnCo_x_‐E with varying Mn/Co ratios.

MnCo_4_‐E was synthesized via an in situ hard‐templating strategy (Figure 1a). Under alkaline conditions, a silicon precursor was introduced, followed by controlled addition of metal salts, drying, and calcination to form MnCo_4_Si. Subsequent selective alkaline etching removed SiO_2_ to yield MnCo_4_‐E. TEM images and elemental mapping of MnCo_4_Si and MnCo_4_‐E (Figure 1b,c) show a uniform distribution of Mn, Co, and Si. Notably, MnCo_4_Si exhibits a thin silica overlayer on the external surface, whereas MnCo_4_‐E shows a more open porous structure (Figure S1). High‐resolution TEM (HRTEM) images (Figure 1d,e; Figure S2) show characteristic morphologies of spinel‐type nanoparticles for all samples, with no detectable segregation of MnO_x_ or CoO_x_ phases. Combined with EDS mapping (Figure 1b,c; Figure S3), the uniform spatial distribution of Mn and Co confirms the formation of a homogeneous Mn─Co composite oxide. N_2_ adsorption–desorption isotherms exhibit a type‐IV adsorption isotherm with pronounced hysteresis loops at the p/p_0_ >0.6 for all catalysts, indicative of a mesoporous structure (Figure S4). MnCo_4_Si displays an H2(b) type hysteresis loop consistent with densely packed spherical particles with pores, while MnCo_4_‐E shows the presence of slit‐ or wedge‐shaped pores. Catalysts subjected to different etching times (Figure S5 and Table S1) exhibit type‐IV isotherms with capillary condensation at high relative pressures, confirming the generation of a stacked mesoporous structure by selective silica leaching. Compared to MnCo_4_‐T (Table S2), the MnCo_4_‐E catalysts show an increase in specific surface area from 68.6 to 81.8 m^2^ g^−1^ and pore volume from 0.15 to 0.31 cm^3^ g^−1^. Such porous architecture with high surface area and pore volume improves reactant accessibility to active sites.

ICP‐OES analysis reveals that MnCo_4_‐E has a Mn/Co/Si atomic ratio of 15.16:55.18:0.30 (Table S3). This corresponds to a Mn/Co ratio of 1:3.64, aligning with the ratios of MnCo_4_‐T (1:3.35) and MnCo_4_Si (1:3.37), and is close to the designed ratio of 1:4. Notably, Mn and Co show no detectable leaching during alkaline etching, as confirmed by ICP‐OES (Table S4). After 2 h of etching, most of the Si is removed, leaving approximately 1.02 wt. % Si (XRF), and the remaining Si species are highly dispersed according to the EDS mapping. Figure 1f presents the XRD patterns of MnCo_4_‐T, MnCo_4_Si, and MnCo_x_‐E, illustrating how varying Mn/Co ratios affect the crystalline structure. MnCo_4_‐E displays characteristic diffraction peaks at 30.5°, 36.0°, 43.8°, 57.9°, and 63.6°, indexed to the (220), (311), (400), (511), and (440) planes of the spinel MnCo_2_O_4_ phase (PDF #23‐1237), respectively. Increasing the Mn/Co ratio causes the (311) peak to shift to lower 2θ values (Figure S6), indicating lattice expansion due to Mn incorporation. This is consistent with Mn^3+^ having a larger ionic radius (0.645 Å) compared to Co^3+^ (0.545 Å) [37, 38, 39]. Notably, the crystalline phase remains unchanged regardless of the etching time (Figure S7). Compared to MnCo_4_‐T, the MnCo_4_Si and MnCo_4_‐E exhibit a left shift of the diffraction peaks, possibly due to the presence of lattice‐incorporated Si. ICP‐OES analysis (Table S3) shows that all samples have similar Mn/Co ratios, excluding the possibility that the observed lattice expansion originates from a different Mn/Co molar ratio. HRTEM images (Figure S2) further show that MnCo_4_‐E has increased lattice spacings for the (311) (from 0.242 to 0.245 nm) and (220) planes (from 0.282 to 0.289 nm) compared to MnCo_4_‐T, supporting the XRD findings of lattice expansion. These findings confirm the successful incorporation of Si into the MnCo spinel lattice.

**FIGURE 2:**
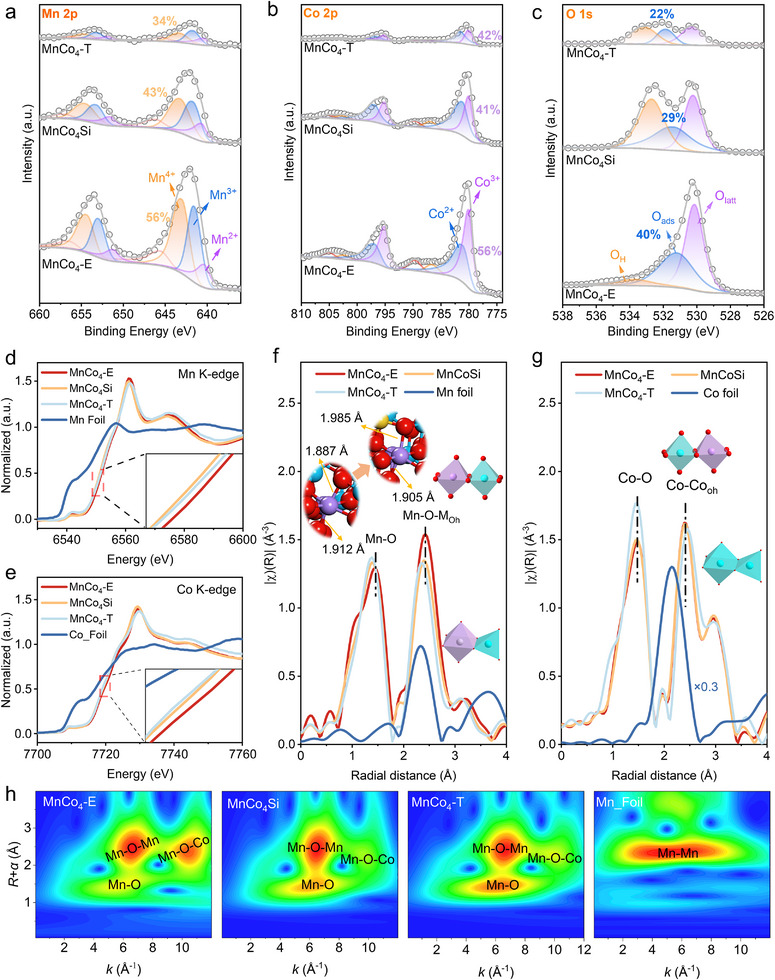
(a) Mn 2p, (b) Co 2p, and (c) O 1s XPS spectra of MnCo_4_‐T, MnCo_4_Si, and MnCo_4_‐E catalysts. Normalized (d) Mn, and (e) Co K‐edge XANES spectra of MnCo4‐E and reference samples. (f) Fourier‐transform Mn, and (g) Co K‐edge EXAFS spectra of MnCo_4_‐E and reference samples. (h) Wavelet transform of the Mn K‐edge EXAFS spectra of MnCo_4_‐E and reference samples.

The surface electronic structures of the MnCo oxide catalysts were investigated by XPS. Mn 2p_3/2_ XPS spectra (Figure 2a) exhibit peaks at ∼643.3, ∼641.7, and ∼640.4 eV, corresponding to Mn^4+^, Mn^3+^, and Mn^2+^ species, respectively [40]. The Co 2p_3/2_ XPS spectra (Figure 2b) can be deconvoluted into Co^3+^ (∼780.0 eV), Co^2+^ (∼781.5 eV), and a satellite peak [33]. Semiquantitative analysis (Table S5) shows that both the Mn^4+^/(Mn^4+^+Mn^3+^+Mn^2+^) and Co^3+^/(Co^3+^+Co^2+^) ratios increase after alkali treatment, with MnCo_4_‐E exhibiting the highest surface concentration of Mn^4+^ and Co^3+^. These results align with H_2_‐TPR profiles (Figure S8a and Table S6), which show four reductive peaks: Mn^4+^ to Mn^3+^, Co^3+^ to Co^2+^, Mn^3+^ to Mn^2+^, and Co^2+^ to Co^0+^, respectively [30, 41, 42]. MnCo_4_‐E displays markedly higher H_2_ consumption at peak 1 (0.69 mmol g^−1^) and 2 (2.62 mmol g^−1^) than MnCo_4_‐T and MnCo_4_Si, confirming the enriched Mn^4+^ and Co^3+^ species. The O 1s spectra (Figure 2c) could be deconvoluted into three peaks at ∼530, ∼531.4, and ∼533.7 eV, which can be ascribed to surface lattice oxygen (O_latt_), surface adsorption oxygen (O_ads_), and surface hydroxyl oxygen in H_2_O (O_OH_) [43], respectively. Notably, MnCo_4_‐E possesses a significantly higher O_ads_ fraction (0.40) than MnCo_4_‐T (0.22) and MnCo_4_Si (0.29), indicating its superior ability to adsorb oxygen. O_2_‐TPD profiles (Figure S8b) show a higher concentration of active oxygen species and superior oxygen storage capacity in MnCo_4_‐E (Table S7). These results demonstrate the enhanced oxygen activation ability for MnCo_4_‐E. In addition, the binding energy of Mn 2p_3/2_ in MnCo_4_‐E (641.78 eV) shifts positively compared to MnCo_4_‐T (641.55 eV), indicating reduced Mn electron density due to electron transfer [44]. This is accompanied by the lower O 1s binding energy, reflecting increased electron density localized on oxygen and decreased electron density on Mn. The electron transfer from Mn to oxygen suggests weakened Mn─O covalency in MnCo_4_‐E [45], which destabilizes the Mn─O bond, lowers the barrier for O─O bond cleavage, and thereby facilitates oxygen activation.

Normalized Mn and Co K‐edge X‐ray absorption near‐edge structure (XANES) spectra reveal average oxidation states between +3 and +4 for Mn and between +2 and +3 for Co (Figures S9 and S10). For MnCo_4_‐E, both Mn and Co K‐edge absorption edges shift to higher energies (Figure 2d,e), indicating increased average oxidation states, in agreement with the XPS and H_2_‐TPR results. Quantitative analysis against reference oxides (Figure S11) shows a gradual increase in Mn valence from 3.52 (MnCo_4_Si) to 3.69 (MnCo_4_‐E), higher than MnCo_4_‐T (3.67). The elevation in oxidation state, induced by etching, is corroborated by time‐resolved Mn K‐edge XANES measurements (Figure S12), which show a continuous edge shift to higher energies with prolonged etching. The corresponding Fourier‐transformed Mn K^2^‐weighted extended X‐ray absorption fine structure (EXAFS) spectra (Figure 2f) of MnCo_4_‐T display two dominant peaks assigned to Mn─O and Mn─O─Mn/Co coordination shells located at 1.91 and 2.87 Å. For MnCo_4_‐E, these peaks were slightly stretched to 1.93 and 2.89 Å, respectively. The corresponding Co K‐edge EXAFS spectra (Figure 2g) exhibit three well‐defined coordination features: a primary Co─O scattering path at ∼1.94 Å, an octahedral Co─O─Co/Mn contribution at ∼2.85 Å, and a tetrahedral Co─O─Co contribution at ∼3.36 Å (Fitting results are presented in Figures S13 and S14, and Tables S8 and S9). The Mn─O─Co coordination confirms the atomic‐level integration of Mn and Co within the MnCo spinel lattice, aligning with XRD and HRTEM analysis. In the octahedral field, the pre‐edge feature of Mn K‐edge spectra (Figure S15) corresponds to the 1s → 3d electronic transition. The highly symmetric MnCo_4_Si configuration renders this transition dipole‐forbidden, yielding characteristically weak pre‐edge intensity. In contrast, the low symmetry MnCo_4_‐T configuration allows the 1s → 3d transition to become dipole‐allowed, leading to a significant enhancement of pre‐edge intensity [46]. Si incorporation induces distinct distortion of the [MnO_6_] octahedra, including axial bond elongation and equatorial bond contraction, whereas MnCo_4_‐E maintains relatively high structural symmetry. DFT calculations reveal axial Mn─O bond elongation in MnCo_4_‐E (1.985/1.905 Å) compared to MnCo_4_‐T (1.887/1.912 Å), suggesting polarization of the Mn─O bond due to Si incorporation. The elongation indicates weakened Mn─O covalency in Si‐incorporated MnCo spinel. The higher electronegativity of Si drives electron redistribution, promoting electron transfer to O─Si bonds, which leads to elongation and weakening of Mn─O bonds. This electron transfer increases the Mn oxidation state, thereby decreasing the local electron density, in line with XPS results. A Si‐doping model was constructed based on XRD and XPS data, and DFT calculations were carried out to investigate the effect of the electronic structure of the catalyst. Electron localization function (ELF) analysis (Figure S16) further confirms this redistribution, showing electron depletion around Mn centers and accumulation near O─Si bonds, indicative of reduced electron density along Mn─O bonds and thus weakened Mn─O covalency. This electronic configuration facilitates electron transfer along the Mn─O σ bond and increases the local charge density on active oxygen species. To investigate the evolution of Mn─O bond strength, Raman spectroscopy was performed (Figure S17a). The characteristic band at 685 cm^−1^, assigned to the symmetric stretching vibration of Mn─O bonds in [MnO_6_] octahedra, exhibits a pronounced red shift upon etching. A similar shift is also observed with decreased Mn content, suggesting the weakened Mn─O bond strength. Furthermore, the force constant of Mn─O bonds was calculated using Hooke's law (Figure S17b), quantitatively confirming the gradual weakening of Mn─O bonds. The observed Mn─O bond elongation and charge redistribution further confirm that Si incorporation weakens Mn─O covalency in MnCo spinel. Wavelet transformed (WT) EXAFS analysis (Figure 2h; Figures S18 and S19) was employed to achieve simultaneous resolution in both R‐space and k‐space for the scattering environment. The Contour plots display a dominant intensity at 5.9 Å^−1^ in k‐space and ∼1.5 Å in R‐space, corresponding to the first‐shell Mn─O scattering, though with notably weakened Mn─O bond strength and reduced coordination numbers compared to reference catalysts, consistent with quantitative fitting results. Notably, MnCo_4_‐E exhibits two distinct intensity maxima at ∼2.5 Å in R‐space, located at *k* = 6.7 and 10.4 Å^−1^, assignable to Mn─O─Mn and Mn─O─Co coordination, respectively.

### 2.2 Catalytic Performance

**FIGURE 3:**
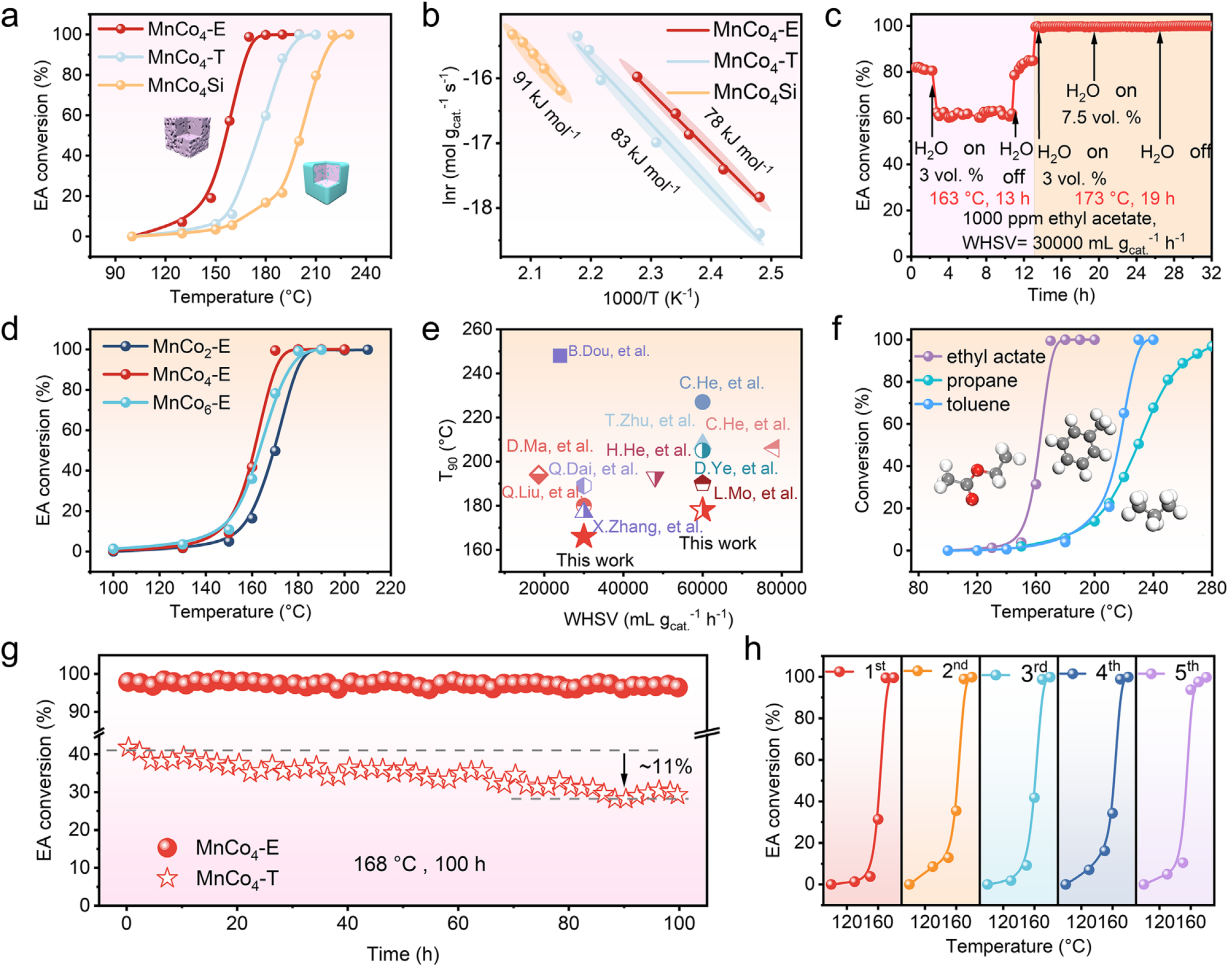
(a) Catalytic oxidation of EA over different catalysts, (b) the corresponding Arrhenius plots, (c) Influence of water vapor on EA conversion of MnCo_4_‐E, (d) EA conversion over catalysts with different Mn/Co ratios, (e) Specific activities of the catalysts synthesized in this work compared with previously reported catalysts. (f) Conversion of typical VOCs (EA, toluene, and propane), (g) Long‐term stability tests, and (h) Cycling stability tests. Reaction conditions: (a, d, g, and h) 1000 ppm EA + 20 % O_2_ balanced with N_2_, WHSV = 30000 mL h^−1^ g_cat._^−1^, (f) 2000 ppm C_3_H_8_ + 10 % O_2_ balanced with N_2_, WHSV = 36 000 mL h^−1^ g_cat._^−1^, 1000 ppm Toluene + 20 % O_2_ balanced with N_2_, WHSV = 36 000 mL h^−1^ g_cat._^−1^.

Catalytic performance of the as‐prepared catalysts was evaluated for the oxidation of EA. The temperature for 10 %, 50 %, and 90 % conversion (T_10_, T_50_, and T_90_) of EA was used to compare the catalytic activity (Figure S20). Among the catalysts, MnCo_4_‐E exhibits the lowest T_90_, outperforming both MnCo_4_Si and MnCo_4_‐T. The catalytic activity improves gradually with increasing alkali‐etching time and reaches a stable state after 2 h of treatment (Figure S21). As shown in Figure 3a, MnCo_4_‐E achieves complete EA conversion below 170°C, whereas MnCo_4_‐T displayed less than 40 % EA conversion at this temperature. The mineralization curve follows the conversion curve closely (Figure S22), indicating nearly complete EA degradation with minimal intermediates accumulation. The superior performance of MnCo_4_‐E was possibly ascribed to the improved oxygen activation ability and more active sites. In addition, the reaction rate (Figure S23) at 168°C of MnCo_4_‐E (4.1 nmol m^−2^ s^−1^) is higher than that of MnCo_4_‐T (1.7 nmol m^−2^ s^−1^) and MnCo_4_Si (0.3 nmol m^−2^ s^−1^). Kinetic studies (Figure 3b; Table S10) reveal apparent activation energy (E_a_) in the order MnCo_4_‐E (78 kJ mol^−1^)< MnCo_4_‐T (83 kJ mol^−1^)< MnCo_4_Si (91 kJ mol^−1^), consistent with the observed activity sequence. The lower E_a_ of MnCo_4_‐E underscores its superior intrinsic reactivity toward EA oxidation. Furthermore, other catalysts synthesized through the in situ hard‐template strategy all markedly improved EA degradation over MnSi‐E, CoSi‐E, MnCeSi, and MnCoSi (Figure S24), decreasing T_90_ by 17°C, 32°C, 16°C, and 40°C, respectively. These results indicate that the in situ hard‐templating approach exerts a more substantial influence on transition metal oxide catalysts, effectively regulating their structural features and optimizing active site accessibility for enhanced oxidation performance.

The influence of water vapor on the catalytic performance of MnCo_4_‐E is shown in Figure 3c. At 163°C, the introduction of 3 % H_2_O immediately suppresses EA conversion from ∼80 % to ∼60 %, and remains stable for 9 h. Notably, full activity recovery was observed upon H_2_O removal, indicating a reversible inhibition process dominated by competitive adsorption. Intriguingly, at the evaluated temperature of 173°C, MnCo_4_‐E exhibits exceptional water tolerance, maintaining stable activity even in the presence of 3 % and 7.5 % H_2_O. This behavior is indicative of a thermally induced desorption process, wherein the strength of water adsorption weakens once the reaction temperature surpasses the desorption threshold. In situ DRIFTS (Figure S25) further support this interpretation: below 110°C, surface sites are largely occupied by adsorbed H_2_O, whereas above 170°C, the disappearance of water vibrational signals aligns with the weakening of carboxylate bands, showing that less water facilitates oxidation of acetate intermediates.

MnCo_2_‐E and MnCo_6_‐E were also evaluated for EA oxidation (Figure 3d). T_90_ values (Figure S20) rank the activity as MnCo_4_‐E >MnCo_6_‐E >MnCo_2_‐E. Notably, MnCo_4_‐E achieves the lowest T_90_ under the reaction conditions (Figure 3e; Table S11), surpassing previously reported transition metal oxides and various noble metal catalysts. To evaluate its practical applicability, MnCo_4_‐E was further tested for the oxidation of other typical VOCs, such as toluene and propane, achieving T_90_ values of 225 and 260°C, respectively (Figure 3f). The catalyst also shows excellent long‐term durability, maintaining 97 % EA conversion over 100 h at 168°C, whereas MnCo_4_‐T loses 11 % activity (from 40 % to 29 %) under identical conditions (Figure 3g). NH_3_‐TPD‐MS profile (Figure S26) shows that MnCo_4_‐E exhibited weaker acidity than MnCo_4_Si after alkaline etching, which will effectively lower carbon deposition on the surface. The moderate surface acid may account for the long‐term durability of MnCo_4_‐E. Cycling stability tests further confirmed the robustness of MnCo_4_‐E, with no detectable deactivation and even slight performance enhancement.

### 2.3 Insights Into the Enhanced Oxygen Activation by Mn─O Covalency Modulation

The excellent performance of MnCo_4_‐E is closely related to the enhanced oxygen activation ability. To understand how Mn─O covalency modulation drives this, oxygen activation behaviors were investigated using O_2_‐TPD‐MS and DFT calculations by assessing dynamic OV formation and replenishment. O_2_‐TPD profiles show a clear desorption peak for adsorbed oxygen species on MnCo_4_‐E (Figure S27). Increasing the Co content lowers the desorption temperature but reduces oxygen storage capacity, whereas higher Mn content increases oxygen storage and shifts the desorption peak to higher temperatures. The optimal Mn/Co ratio of 1:4 thus balances oxygen activation and storage, in line with the superior performance of MnCo_4_‐E. Cyclic O_2_‐TPD‐MS analysis further elucidates the origin of oxygen activation and the excellent cycling stability of MnCo_4_‐E in EA oxidation (Figure S28). Two main oxygen species are identified: (i) physically and chemically adsorbed oxygen species (50°C–250°C), and (ii) lattice oxygen (>250°C) [24, 47]. To probe O_2_ adsorption ability, the catalysts were heated sequentially to 250°C and 450°C, then cooled to room temperature and re‐exposed to an O_2_/He flow. Distinct behaviors are observed for the two catalysts. For MnCo_4_‐T, the desorption peak shifts to higher temperatures, consistent with hindered oxygen migration and reduced lattice oxygen reactivity. In contrast, MnCo_4_‐E shows a shift of the oxygen desorption onset to lower temperatures after oxygen replenishment, indicating accelerated oxygen‐exchange kinetics.

**FIGURE 4:**
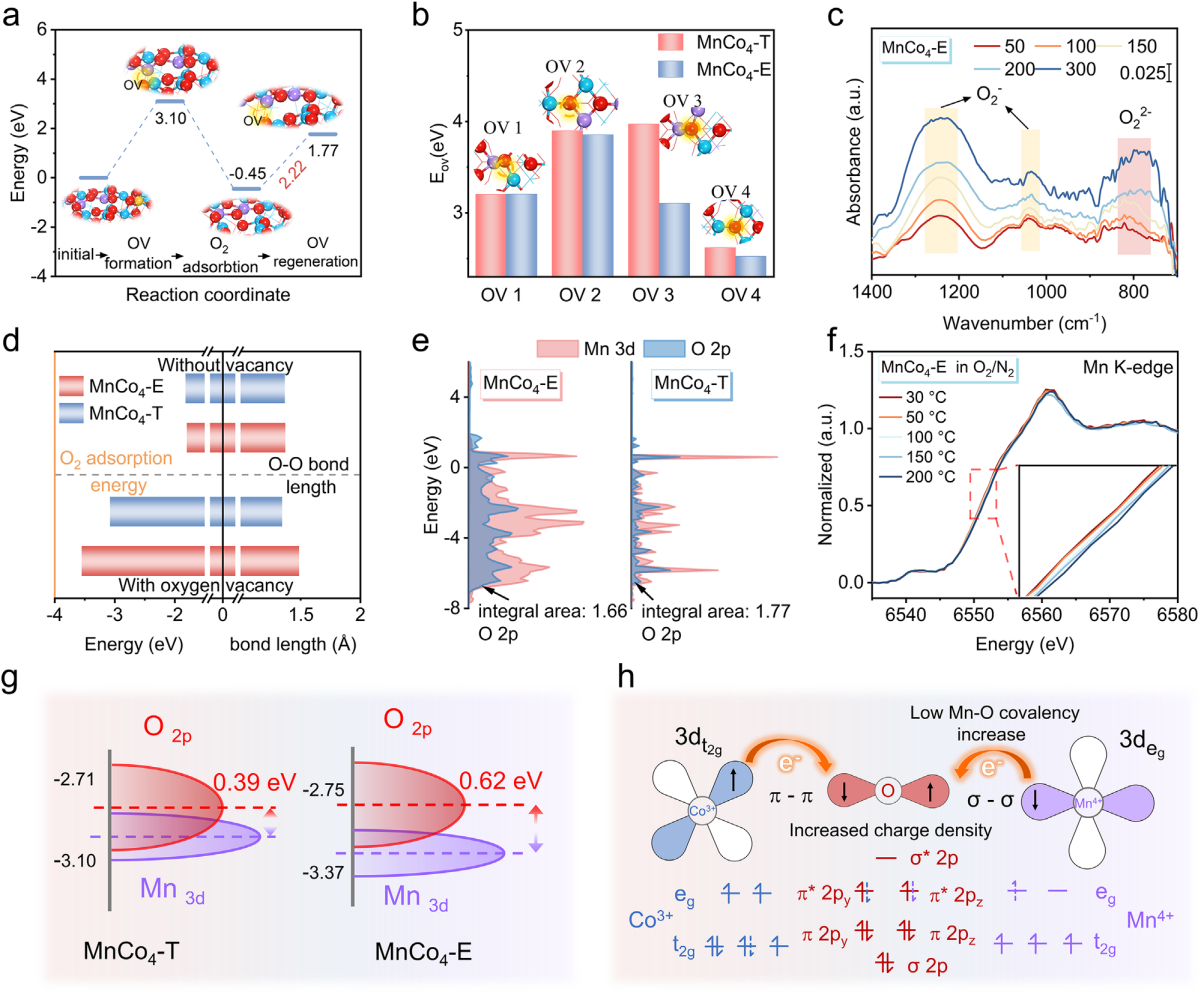
(a) Energy diagrams for the formation of OV and O_2_ adsorption on OV. (b) DFT‐calculated oxygen vacancy formation energy at different sites in MnCo_4_‐E and MnCo_4_‐T. (c) In situ DRIFTS of O_2_ adsorption on MnCo_4_‐E under 20 % O_2_/N_2_ at different temperatures. (d) O_2_ adsorption energies and corresponding O─O bond length for MnCo_4_‐E and MnCo_4_‐T in the presence and absence of oxygen vacancy. (e) The calculated partial DOS of O and Mn in MnCo_4_‐E and MnCO_4_‐T. (f) In situ normalized Mn K‐edge XANES spectra under O_2_/N_2_ atmosphere from 30°C to 200°C. (g) Schematic diagram of the band structures of MnCo_4_‐T and MnCo_4_‐E. (h) The mechanism of MnCo_4_‐E enhanced oxygen activation ability via Mn─O covalency modulation.

DFT calculations (Figure 4a) reveal that MnCo_4_‐E has an initial oxygen vacancy formation energy (E_OV_) of 3.10 eV, accompanied by spontaneous migration of subsurface oxygen to the surface. Under an O_2_ atmosphere, these vacancies readily adsorb and activate O_2_, generating active oxygen species with adsorption energies significantly higher than those on a defect‐free surface. Notably, the subsequent vacancy formation energy drops to 2.22 eV, enabling efficient and sustained oxygen depletion‐replenishment cycles, which account for the good cycling performance and long‐term stability. During EA oxidation, surface lattice oxygen directly participates in reactant activation, and the remaining OVs are subsequently replenished by gaseous O_2_, following the Mars‐van Krevelen (MVK) mechanism. As a result, both OV formation and replenishment govern the overall catalytic activity of MnCo_4_‐E. Theoretical calculations further highlight the key role of local oxygen coordination (Figure 4b; Figure S29). Among the accessible sites, the OV_4_ site with higher Co coordination exhibits the lowest oxygen vacancy formation energy (E_OV_ = 2.62 eV), suggesting that Co‐rich sites serve as preferential centers for OV generation. This is consistent with the experimental results, that is, increasing Co content lowers the onset of oxygen desorption and improves low‐temperature activity.

The weakened Mn─O covalency induced by Si incorporation further lowers oxygen desorption temperatures. This electronic modulation facilitates oxygen activation and enhances the efficiency of redox cycling. The nature of the activated oxygen species was further investigated by in situ DRIFTS during H_2_ oxidation in the absence of O_2_ (Figures S30 and S31). Three distinct oxygen species are observed, including bridge‐type (M^+^─O^2−^─M^+^, 750–800 cm^−1^), terminal‐type (M^2+^─O^2−^, 1300–1400 cm^−1^), and adsorbed molecular oxygen (M^+^─O_2_^−^,1100–1120 cm^−1^) [48, 49]. The oxidation products appear as hydroxyl groups (─OH, 3600–3660 cm^−1^) and absorbed water (δ(H_2_O), 1596 cm^−1^). Above 150°C, water‐related vibrational features increase sharply, while the M^2+^─O^2−^ bands weaken rapidly between 150°C and 250°C, indicating that surface lattice oxygen acts as the dominant reactive species in this temperature range. These results suggest that M^2+^─O^2−^ can react with H_2_ above 150°C to generate H_2_O and terminal‐type OV (bare M), which is consistent with the enhanced intensity of δ(H_2_O). Notably, above 250°C, the M^2+^─O^2−^ bands recover as the M^+^─O_2_^−^ bands weaken, indicating transformation of M^+^─O_2_^−^ to M^2+^─O^2−^ species [50]. The gradual increase in *v*(OH) intensity at 3660 cm^−1^ above 200°C indicates OH formation via interaction of H_2_O with M^2+^─O^2−^ sites, whereas the emergence of M^+^─O^2−^─M^+^ and M^+^─O^−^ vibrations implies surface reconstruction.

The oxygen replenishment capability of MnCo_4_‐E was further examined under an H_2_/Ar + O_2_/N_2_ atmosphere (Figure S32). Upon O_2_ introduction, the *v*(OH) bands decrease with temperature, opposite to their behavior under H_2_/Ar, while the intensified H_2_O vibration at 3400 cm^−1^ provides direct evidence for the hydroxyl‐assisted surface redox cycle. A similar behavior is also observed during in situ DRIFTS of EA oxidation (Figure S33). This process accelerates hydroxyl consumption, thereby weakening the M─OH peak intensity. In the presence of O_2_, the loss of surface oxygen during heating is also markedly smaller than in the absence of O_2_, indicating continuous replenishment of active oxygen species from the gas phase. These results establish that O_2_ activation promotes the surface redox cycle and maintains oxygen availability under reaction conditions. In situ DRIFTS during the regeneration of H_2_‐reduced MnCo_4_‐E under O_2_/N_2_ (Figure S34) further reveals the dynamic self‐healing of surface oxygen species. At near‐ambient temperature (50°C), the bridging M^+^─O^2−^─M^+^ and M^+^─O^−^ vibrations recover to pristine states, accompanied by restoration of M^2+^─O^2−^ species, demonstrating exceptional oxygen self‐healing ability. At operating temperatures above 150°C, full recovery is observed as M^+^─O_2_^−^ and M^2+^─O^2−^ vibrations grow in intensity, suggesting that rapid regeneration of M^+^─O_2_^−^ intermediates facilitates subsequent reformation of M^2+^─O^2−^ sites and thus ensures efficient redox cycling.

The dynamic evolution of active oxygen species was investigated by in situ DRIFTS of O_2_ adsorption and activation (Figure 4c; Figure S35). After purging MnCo_4_‐E with O_2_, bands assigned to superoxide (O_2_^−^; 1037 and 1238 cm^−1^) and peroxide (O_2_^2−^; 814 cm^−1^) species appear and remain thermally stable with increasing temperature. In contrast, MnCo_4_‐T generates mainly O_2_^−^ species with weaker intensity under heating. The pronounced O_2_^−^ and O_2_^2−^ signals on MnCo_4_‐E confirm its superior O_2_ activation ability, which results from the weakened Mn─O covalency. DFT calculations further show that Mn─O covalency modulation lowers the O_2_ dissociation barrier and promotes the formation of active oxygen species (Figure 4d; Figure S36). For MnCo_4_‐E, O_2_ adsorption induces O─O bond elongation from 1.23 to 1.37 Å (O_2_^−^, −1.80 eV adsorption energy) on defect‐free surface, and further to 1.49 Å (O_2_^2−^, −3.55 eV) at OV sites, underlining superior oxygen activation ability [51]. In contrast, MnCo_4_‐T is limited to O_2_^−^ formation, independent of vacancy presence (Figure S37). EPR (Figure S38) shows a signal at g = 2.003, assigned to unpaired electrons at OV sites, with intensities following the order MnCo_4_‐E< MnCo_4_‐T< MnCo_4_Si, indicating a gradual increase in the OV concentration. The weaker signal for MnCo_4_‐E arises from its superior low‐temperature oxygen activation ability, whereby O_2_ rapidly replenishes OV, consistent with the O_2_ DRIFTS and DFT calculation results. Bader charge analysis (Figures S39 and S40) shows more electron transfer from MnCo_4_‐E (0.559|e|) than from MnCo_4_‐T (0.544|e|) to O_2_. The transferred electrons primarily occupy the π^*^2p antibonding orbital of O_2_, lowering the bond order and facilitating O_2_ activation. PODS analysis (Figure 4e) indicates that the reduced hybridization between Mn 3d and O 2p orbitals weakens Mn─O covalency, limiting electron back‐donation from oxygen to Mn while increasing electron density on oxygen. Owing to the high electronegativity of oxygen, the weakened Mn─O covalency facilitates electron transfer from Mn to O along Mn─O σ bonds, in line with the XAFS and XPS data. This charge redistribution supports the formation of reactive oxygen species that are more readily involved in catalytic redox cycles.

Under O_2_/N_2_ atmosphere (Figure 4f; Figure S41), the Mn K‐edge in the XANES region shifts to higher energy with increasing temperature, indicating higher Mn oxidation state and oxygen activation on MnCo_4_‐E during heating. EXAFS fitting (Figure S42 and Table S12) shows the Mn─O coordination number increases from 4.3°C at 50°C to 4.6°C at 200°C, with a slight Mn─O bond elongation (1.91 Å → 1.92 Å). This is consistent with the O_2_‐DRIFTS data, confirming the improved oxygen dissociation ability of MnCo_4_‐E. Based on the PDOS calculations, the schematic in Figure 4g illustrates the Mn 3d band center, O 2p band center, and their coupling as a function of Mn─O covalency. The rapid low‐temperature conversion of acetate and formate intermediates over MnCo_4_‐E indicates that tuning Mn─O covalency effectively promotes oxygen activation and lowers the reaction temperature. During oxygen activation (Figure 4h), electrons in the Co 3d t_2g_ orbitals interact with O π^*^_2p_ orbitals through π‐type bonding, whereas electrons in the Mn 3d orbitals interact with O π^*^_2p_ through σ‐type bonding. The downward shift of the Mn 3d band center weakens Mn─O hybridization, indicative of reduced Mn─O covalency. This electronic configuration facilitates electron transfer along the Mn─O σ bond and increases the local charge density on active oxygen species, thereby enhancing their activation and reactivity in VOCs oxidation.

### 2.4 Reaction Mechanism of Ethyl Acetate Oxidation Over MnCo_4_‐E

**FIGURE 5:**
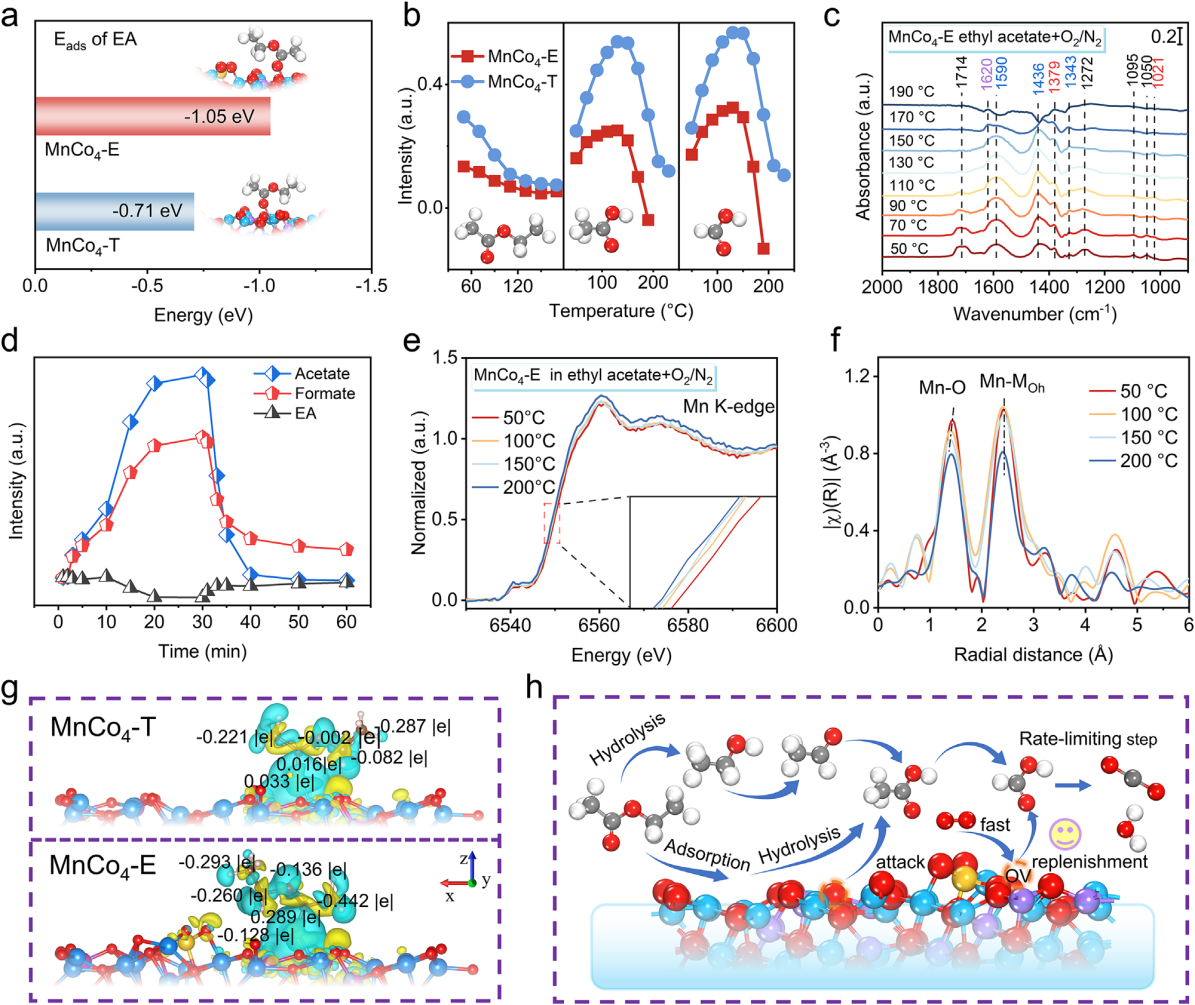
(a) Optimized adsorption energies of ethyl acetate molecules on MnCo_4_‐E and MnCo_4_‐T. (b) Temperature‐dependent evolution of EA, acetate, and formate over MnCo_4_‐E during absorption in N_2_ and oxidation in O_2_/N_2_. (c) In situ DRIFTS of EA oxidation over MnCo_4_‐E in O_2_/N_2_ from 50°C to 190°C. (d) Temperature‐dependent evolution of EA, acetate, and formate over MnCo_4_‐E during absorption in N_2_ and oxidation in O_2_/N_2_ at 170°C. (e) Normalized Mn K‐edge XANES spectra and (f) EXAFS spectra of EA oxidation over MnCo_4_‐E from 50°C to 200°C. (g) Bader charge analysis of ethyl acetate for theoretical models of MnCo_4_‐T and MnCo_4_‐E. (h) Proposed reaction pathway for EA oxidation over MnCo_4_‐E.

The mechanism of EA oxidation over MnCo_4_‐E, MnCo_4_‐T, and MnCo_4_Si was investigated by in situ DRIFTS. As shown in Figure S43, seven distinct bands are observed on MnCo_4_‐E at 30°C. The bands at 1716, 1268, and 1095/1050 cm^−1^ are assigned to stretching vibrations of C═O, C─O─C, and C─O in absorbed EA, respectively [52, 53]. The peak at 1427 cm^−1^ corresponds to the symmetric stretching vibration of carboxylate in acetate species, and the bands at 1590 cm^−1^ and 1336 cm^−1^ are assigned to the asymmetrical and symmetrical stretching vibration of carboxylate in formate intermediates [54, 55]. Time‐resolved DRIFTS shows that EA can adsorb on MnCo_4_‐E at ambient temperature and rapidly decomposes into acetate and formate species. Notably, the C═O stretching vibration (1716 cm^−1^) of molecularly adsorbed EA is markedly intensified on MnCo_4_‐T and MnCo_4_Si than on MnCo_4_‐E (Figures S44 and S45), indicating that MnCo_4_‐E exhibits higher hydrolysis activity. This is further supported by the rapid appearance of carboxylate bands at 1548 and 1427 cm^−1^, which demonstrates efficient conversion of EA to surface intermediates. With prolonged adsorption, carboxylate species gradually accumulate on MnCo_4_‐E, while surface hydroxyl groups are consumed, suggesting that hydroxyl participates in promoting C─O bond cleavage during EA hydrolysis. Although acetate accumulation partially suppresses further reaction, the stronger carboxylate signals observed on MnCo_4_‐E compared with MnCo_4_‐T and MnCo_4_Si indicate the presence of more active sites. DFT calculations (Figure 5a; Figure S46) further show that EA adsorbs more strongly on MnCo_4_‐E (E_ads_ = −1.05 eV) than on MnCo_4_‐T (E_ads_ = −0.71 eV).

In situ DRIFTS shows that, on MnCo_4_‐E, alkoxide intermediates are rapidly oxidized to aldehyde and then to carboxylates by surface oxygen species. This is attributed to the weakening of Mn─O covalency induced by Si, which facilitates electronic transfer from Mn to oxygen. In addition, the elongation of the Mn─O bond is prone to releasing oxygen to form active oxygen species. Furthermore, the superior redox cycling ability facilitates the conversion of intermediates such as alkoxides and aldehydes. In contrast, MnCo_4_‐T exhibits slower oxidation, leading to the accumulation of alkoxide species (Figure S44). As the temperature increases, the bands at 1716, 1268, 1095, and 1050 cm^−1^ associated with adsorbed EA, gradually weaken and disappear completely at 130°C (Figure 5b,c), while the accumulation of carboxylate species reached a maximum. The intensity of acetate (1427 cm^−1^) and formate (1584 cm^−1^) species begins to decrease above 130°C and 140°C on MnCo_4_‐E and MnCo_4_‐T, respectively, indicating that their conversion is the rate‐limiting step. The acetate intermediates proceed through a formate‐based pathway, achieving full mineralization at 170°C, which is notably lower than the temperature required for MnCo_4_‐T preparation (190°C). Under anaerobic conditions (Figure S47), the gradual depletion of surface‐adsorbed oxygen species, accompanied by the accumulation of acetate intermediates, leads to a marked loss of catalytic oxidation capacity. This promotes the formation of olefinic byproducts, as seen the emergence of the characteristic ethylene vibrational at 928 cm^−1^. The carboxylate species gradually diminish above 170°C, indicating the participation of surface lattice oxygen in the oxidation process. Complete degradation of acetate species occurs before 250°C under anaerobic conditions, confirming the complete mineralization. As shown in Figure 5d and Figure S48, EA first adsorbs on MnCo_4_‐E at 170°C, but quickly undergoes hydrolysis to carboxylate species, as evidenced by the disappearance of the characteristic EA vibrational bands. With prolonged reaction time, the depletion of active oxygen species weakens the oxidation capacity of the catalyst, leading to the accumulation of carboxylates, olefinic byproducts, and aldehyde intermediates. Upon switching to a 20 % O_2_/N_2_ atmosphere, the accumulated carboxylates are rapidly oxidized by reactive oxygen species, and the intermediates are completely consumed without byproduct formation. In situ XAFS was used to probe the dynamic evolution of metal valence states and metal‐oxygen coordination in MnCo_4_‐E under different atmospheres and temperatures to elucidate lattice oxygen participation and replenishment during oxidation. Under reaction conditions (EA in air), both Mn and Co K edges shifted to lower energy with increasing temperature (Figure 5e; Figure S49), indicating reduction of the metal oxide due to consumption of surface lattice oxygen and OV formation. A pre‐edge peak at ∼6540 eV, corresponding to the dipole‐forbidden but quadrupole‐allowed 1s → 3d transition, demonstrates 3d‐4p orbital hybridization at the Mn center in a non‐centrosymmetric environment. The increasing pre‐edge intensity during the reaction indicates a symmetry‐lowering transition in Mn coordination, evolving from an octahedral‐like toward a more tetrahedral‐like geometry. The structure evolution of MnCo_4_‐E was further investigated by in situ EXAFS (Figure 5f). As temperature increases under EA flow, the attenuation of first‐shell Mn─O scattering intensity indicates a gradual decrease in Mn─O coordination number. EXAFS fitting (Figure S50 and Table S13) shows a decrease in coordination number from 4.0°C at 50°C to 3.4°C at 200°C, accompanied by a slight Mn─O bond contraction (1.91 Å → 1.90 Å). These changes are consistent with the Mn valence reduction observed in XANES (Figure 5e), induced by continuous consumption of lattice oxygen. At lower temperatures (<150°C), the Mn─O scattering intensity decreased significantly, while the Mn─O─Co coordination is largely maintained, suggesting preferential loss of terminal oxygen species during EA hydrolysis without influencing the Mn─O─Co coordination. Above 150°C, the bridge oxygen participates in acetate degradation, leading to the formation of bridging OV and local coordination rearrangement. These results confirm that EA oxidation over MnCo_4_‐E follows the MvK mechanism. Charge density difference analysis (Figure 5g) further reveals distinct electronic interactions between EA and the catalysts. On MnCo_4_‐E, there is a clear electron transfer from the MnCo spinel surface to the adsorbed EA, whereas this effect is much weaker on MnCo_4_‐T. This charge redistribution facilitates oxidation on MnCo_4_‐E, leading to electron enrichment in the α‐carbon of the acetate species. The electron‐rich α‐carbon becomes particularly vulnerable to attack by surface lattice oxygen, facilitating C─C bond cleavage, which is a key step in acetate oxidation. Figure 5h therefore presents the proposed degradation mechanism of EA over MnCo_4_‐E, in which the oxidation of carboxylate intermediates is rate‐limiting.

## 3 Conclusion

In summary, Mn─O covalency was successfully tuned in a CoMn spinel catalyst via an in situ hard‐templated method. Weakening the Mn─O covalency promotes electron transfer from Mn to O, creating effective oxygen activation centers that enhance both gaseous and lattice oxygen activation. This accelerates the rate‐limiting carboxylate decomposition step at relatively low temperatures, and simultaneously improves water‐resistance as well as long‐time durability (100 h). The catalyst also shows efficient performance for the oxidation of other typical VOCs, including aromatic hydrocarbons (toluene) and alkanes (propane). This work highlights the significant role of metal‐oxygen covalency in oxygen activation for VOCs oxidation and demonstrates that Si incorporation and reduced Mn─O covalency offer a promising route to design efficient catalysts with strong oxygen activation ability for enhanced VOCs degradation.

## 4 Experimental Section

### 4.1 Catalyst Synthesis

MnCo_x_Si was synthesized by a modified Stöber method. Typically, a certain amount of Mn(NO_3_)_2_ solution (50 wt. %) and Co(NO_3_)_2_·6H_2_O was dissolved in 50 mL of ethanol and stirred thoroughly to obtain solution A. Meanwhile, 6 mL of water, 100 mL of ethanol, and 8 mL of ammonia solution were mixed in another beaker. Afterward, the mixture was vigorously stirred for 10 min at room temperature to form a homogeneous solution. Subsequently, 2 g of tetraethyl orthosilicate (TEOS) was added. Once the hydrolysis of TEOS occurred, solution A was added, and the pH of the resulting mixture was adjusted to 10. The reaction mixture was then continuously stirred for 12 h, followed by static aging for 1 h. Subsequently, the supernatant was carefully removed, and the remaining solid was dried in an oven at 110°C for 24 h and then calcined in a muffle furnace at 400°C for 4 h to obtain the MnCo_4_Si catalyst.

MnCo_4_‐E was synthesized by an in situ hard‐template method. First, 1 g MnCo_4_Si catalyst was added into 50 mL of 2 mol L^−1^ NaOH solution, stirred at 80°C for 120 min, centrifuged, washed, and then dried to obtain the MnCo_4_‐E catalyst.

MnCo_4_‐T was prepared via the co‐precipitation method, following the analogous steps as those for MnCo_4_Si, with the exception that TEOS was not added.

The synthesis details for the other catalysts are provided in the Supporting Information.

### 4.2 Catalytic Testing

The catalytic activities of catalysts toward EA oxidation were evaluated in a fixed‐bed quartz tube microreactor (6 mm id × 500 mm length). In each experiment, 80 mg sample was loaded in a quartz tube reactor under a total weight hourly space velocity of 30 000 mL h^−1^ g^−1^. The detailed experiments are provided in the Supporting Information.

### 4.3 Catalyst Characterization

The catalysts were characterized using various techniques such as X‐ray diffraction (XRD), X‐ray photoelectron spectroscopy (XPS), and X‐ray absorption fine structure spectroscopy (XAFS) to provide a comprehensive understanding of their structural and chemical properties. In situ diffuse reflectance infrared Fourier transform spectroscopy (in situ DRIFTS) was also performed to explore the reaction mechanism of EA on catalysts. The detailed characterization procedures were presented in the Supporting Information.

### 4.4 DFT Calculations

All calculations were based on the first principles of Density Functional Theory (DFT), carried out using the Vienna ab‐initio simulation package. The projected augmented wave method was employed for the processing of the Kohn–Sham equation. The generalized gradient approximation and Perdew–Burke–Ernzerhof of exchange‐correlation functional were adopted. The detailed procedures of DFT studies are provided in the SI.

## Conflicts of Interest

The authors declare no conflict of interest.

## Supporting information




**Supporting File**: advs75213‐sup‐0001‐SuppMat.docx.

## Data Availability

The data that support the findings of this study are available from the corresponding author upon reasonable request.
