# Supplementary material for: Reinforcing Oxygen Activation of Spinel Oxide via Mn─O Covalency Engineering for VOCs Oxidation
Source: Adv Sci (Weinh). 2026 Apr 10:e75213. Online ahead of print. doi: 10.1002/advs.75213 (PMC13334685; doi:10.1002/advs.75213)
Supplement: Supplementary file 1 — Supporting File: advs75213‐sup‐0001‐SuppMat.docx. [file ADVS-9999-e75213-s001.docx]

**Supporting Information**

**Reinforcing oxygen activation of spinel oxide via Mn-O covalency engineering for VOCs oxidation**

Gan Li,^a^ Ruochen Zhou,^a^ Qianqian Chen,^a^ Guobo Li,^a^ Xiang Tu,^c^ Fengbo Yu,^a^ Wenming Liu,^b^ Jian Ji,^a*^ Honggen Peng, ^a,b*^

^a^ *School of Resources and Environment, Nanchang University, 999 Xuefu Road, Nanchang, Jiangxi 330031, China*

^b^ *School of Chemistry and Chemical Engineering, Nanchang University, 999 Xuefu Road, Nanchang, Jiangxi 330031, China*

^c^ *Jiangxi Provincial Institute of Eco-Environmental Science Research and Planning, Nanchang, Jiangxi 330039, China*

^*^ Corresponding authors, E-mail: *jijian@ncu.edu.cn* (J Ji)

*penghonggen@ncu.edu.cn* (H Peng)

This Supporting Information consists **76 pages** including experimental details, **50 Figures**, and **13 Tables**.

**Table of Contents**

[Supplementary Experimental Details 5](#_Toc225099138)

[Text S1: Catalysts Synthesis 5](#_Toc225099139)

[Text S2: Catalyst Characterization 5](#_Toc225099140)

[Test S3: Catalytic Testing 7](#_Toc225099141)

[Test S4: Kinetic Studies 8](#_Toc225099142)

[Test S5: DFT Calculations 9](#_Toc225099143)

[Test S6: Detailed in situ experimental tests 10](#_Toc225099144)

[Supplementary Figures 12](#_Toc225099145)

[Figure S1. SEM images of and (a) MnCo_4_-E, (b) MnCo_4_Si and (c) MnCo_4_-T. 12](#_Toc225099146)

[Figure S2. TEM images of (a-c) MnCo_4_-E, (d-f) MnCo_4_Si, and (g-i) MnCo_4_-T. 13](#_Toc225099147)

[Figure S3. (a) STEM image and (b-d) elemental mappings of MnCo_4_-T. 14](#_Toc225099148)

[Figure S4. (a) N_2_ adsorption-desorption isotherms and (b) pore size distribution profiles of MnCo_4_Si, MnCo_4_-T, MnCo_4_-E, and related catalysts. 15](#_Toc225099149)

[Figure S5. (a) N_2_ adsorption-desorption isotherms and (b) pore size distribution profiles of MnCo_4_-E with different alkali-treated time. 16](#_Toc225099150)

[Figure S6. Lattice diffraction peak of the MnCo spinel (311) plane of the MnCo_4_-E and related catalysts. 17](#_Toc225099151)

[Figure S7. The XRD patterns of MnCo_4_-E with different alkali-treated time. 18](#_Toc225099152)

[Figure S8. (a) H_2_-TPR and (b) O_2_-TPD profiles of MnCo_4_-E and related catalysts. 19](#_Toc225099153)

[Figure S9. Normalized Mn K-edge XANES spectra of MnCo_4_-E and standard substance. 20](#_Toc225099154)

[Figure S10. Normalized Co K-edge XANES spectra of MnCo_4_-E and standard substance. 21](#_Toc225099155)

[Figure S11. The valence states of (a) Mn and (b) Co in different substances. 22](#_Toc225099156)

[Figure S12. Fourier-transform Mn K-edge EXAFS spectra of MnCo_4_-E with different alkali-treated time. 23](#_Toc225099157)

[Figure S13. Mn K-edge EXAFS fitting curves of (a-b) MnCo_4_-E, (b-c) MnCo_4_Si, and (e-f) MnCo_4_-T sample in K and R spaces. 24](#_Toc225099158)

[Figure S14. Co K-edge EXAFS fitting curves of (a-b) MnCo_4_-E, (b-c) MnCo_4_Si, and (e-f) MnCo_4_-T sample in K and R spaces. 25](#_Toc225099159)

[Figure S15. Pre-edge spectra of normalized Mn K-edge XANES for MnCo_4_-E and reference catalysts. 26](#_Toc225099160)

[Figure S16. The ELF images of MnCo spinel with Si atoms substituted Co_Td_ sites. 27](#_Toc225099161)

[Figure S17. (a) Raman spectra and (b) Mn-O force constant of prepared catalysts. 28](#_Toc225099162)

[Figure S18. Wavelet transform of the Mn K-edge EXAFS spectra of Mn oxides reference samples. 29](#_Toc225099163)

[Figure S19. Wavelet transform of the Co K-edge EXAFS spectra of MnCo_4_-E and reference samples. 30](#_Toc225099164)

[Figure S20. The activity (a) T_10_, T_50_ and T_90_ of MnCo_4_-E and related catalysts, (b) Ethyl acetate conversation of catalysts with different Mn/Co ratio. 31](#_Toc225099165)

[Figure S21. The ethyl acetate conversion of MnCo_4_-E with different alkali-treated time. 32](#_Toc225099166)

[Figure S22. Plot of ethyl acetate mineralization versus temperature over various catalysts. 33](#_Toc225099167)

[Figure S23. Reaction rates of series catalysts at 168 °C (excluding the effect of specific surface area). 34](#_Toc225099168)

[Figure S24. (a) Catalytic performance and (b) T_90_ temperature of different metal oxide prepared by *in situ* hard-template toward ethyl acetate. 35](#_Toc225099169)

[Figure S25. *In situ* DRIFTS of ethyl acetate oxidation in 5% H_2_O balanced with O_2_/N_2_ (a and a_1_) from 50 to 210 ℃ over MnCo_4_-E. 36](#_Toc225099170)

[Figure S26. NH_3_ TPD-MS results of (a) MnCo_4_-E, (b) MnCo_4_-T, and (c) MnCo_4_Si. 37](#_Toc225099171)

[Figure S27. O_2_-TPD profiles of MnCo series catalysts from 30 to 250 °C. 38](#_Toc225099172)

[Figure S28. Cycling O_2_-TPD-MS of (a) MnCo_4_-E and (b) MnCo_4_-T. 39](#_Toc225099173)

[Figure S29. Top view of oxygen vacancy locations in (a) MnCo_4_-T and (b) MnCo_4_-E. 40](#_Toc225099174)

[Figure S30. *In situ* DRIFTS of H_2_ oxidation by oxygen species at MnCo_4_-E surface in H_2_/Ar at 200 °C as a function of time. 41](#_Toc225099175)

[Figure S31. *In situ* DRIFTS of H_2_ oxidation by oxygen species on MnCo_4_-E surface in H_2_/Ar as a function of temperature. 42](#_Toc225099176)

[Figure S32. *In situ* DRIFTS of H_2_ oxidation by oxygen species on MnCo_4_-E surface in H_2_/Ar +O_2_/N_2_ as a function of temperature. 43](#_Toc225099177)

[Figure S33. *In situ* DRIFTS of ethyl acetate oxidation in O_2_/N_2_ from 50 to 190 °C over MnCo_4_-E. 44](#_Toc225099178)

[Figure S34. *In situ* DRIFTS of MnCo_4_-E catalyst replenishing oxygen vacancies in O_2_/N_2_ as a function of temperature. 45](#_Toc225099179)

[Figure S35. *In situ* DRIFTS of MnCo_4_-T exposed under the 20% O_2_/N_2_ as a function of temperature. 46](#_Toc225099180)

[Figure S36. Optimized adsorption configurations of oxygen molecules on MnCo_4_-E catalysts. 47](#_Toc225099181)

[Figure S37. Optimized adsorption configurations of O_2_ molecules on MnCo_4_-T catalysts. 48](#_Toc225099182)

[Figure S38. EPR profiles of MnCo_4_-E and related catalysts. 49](#_Toc225099183)

[Figure S39. Bader charge variation of ethyl acetate for theoretical models of MnCo_4_-E catalysts. 50](#_Toc225099184)

[Figure S40. Bader charge variation of ethyl acetate for theoretical models of MnCo_4_-T catalysts. 51](#_Toc225099185)

[Figure S41. *In situ* EXAFS spectra of MnCo_4_-E under O_2_/N_2_ flow from 30 to 200 °C. 52](#_Toc225099186)

[Figure S42. Mn K-edge EXAFS K and R space fitting curves of MnCo_4_-E under O_2_/N_2_ flow at (a-b) 50 °C, and (c-d) 100 °C. 53](#_Toc225099187)

[Figure S43. *In situ* DRIFTS of ethyl acetate adsorption on the MnCo_4_-E (a and a_1_) under 20% O_2_/N_2_ flow. 54](#_Toc225099188)

[Figure S44. *In situ* DRIFTS of ethyl acetate (a) absorption in O_2_/N_2_ at 30 °C and (b) oxidation from 50 to 250 °C over MnCo_4_-T. 55](#_Toc225099189)

[Figure S45. *In situ* DRIFTS of ethyl acetate adsorption over MnCo_4_Si from 0 to 30 min at 30 ℃ in (a) O_2_/N_2_ flow. *In situ* DRIFTS of ethyl acetate oxidation in (b) O_2_/N_2_, (c) N_2_ from 50 to 250 ℃ over MnCo_4_Si. 56](#_Toc225099190)

[Figure S46. Optimized adsorption configurations of ethyl acetate on (a) MnCo_4_-T and (b) MnCo_4_-E catalysts. 57](#_Toc225099191)

[Figure S47. *In situ* DRIFTS of EA absorption in N_2_ at 30 °C and oxidation from 50 to 250 °C. 58](#_Toc225099192)

[Figure S48. *In situ* DRIFTS of EA (a) absorption in N_2_ and (b) oxidation in O_2_/N_2_ as a function of time at 170 °C over MnCo_4_-E. 59](#_Toc225099193)

[Figure S49. (a) Normalized Co K-edge *in situ* XANES spectra of MnCo_4_-E and (b) In-situ EXAFS spectra of the transient reaction with ethyl acetate from 30 to 200 °C. 60](#_Toc225099194)

[Figure S50. Mn K-edge EXAFS K and R space fitting curves of MnCo_4_-E under ethyl acetate flow balanced with O_2_/N_2_ at (a-b) 50 °C, (c-d) 100 °C, (e-f) 150 °C, and (g-h) 200 °C. 61](#_Toc225099195)

[Supplementary Tables 62](#_Toc225099196)

[Table S1. Physicochemical properties of catalysts measured by N_2_ sorption isotherms. 62](#_Toc225099197)

[Table S2. Physicochemical properties of catalysts measured by N_2_ sorption isotherms. 63](#_Toc225099198)

[Table S3. The Element content of MnCo_4_-E and related catalysts. 64](#_Toc225099199)

[Table S4. The ICP-OES result of MnCo_4_-E with different alkali-treated time. 65](#_Toc225099200)

[Table S5. XPS semi-quantitative analysis data of MnCo_4_-T and reference catalysts. 66](#_Toc225099201)

[Table S6. H_2_ consumption of MnCo_4_-E and related catalysts. 67](#_Toc225099202)

[Table S7. O_2_ desorption on MnCo_4_-E and related catalysts. 68](#_Toc225099203)

[Table S8. Structural parameters of MnCo_4_-E, MnCo_4_Si, and MnCo_4_-T extracted from the EXAFS fitting. 69](#_Toc225099204)

[Table S9. Structural parameters of MnCo_4_-E, MnCo_4_Si, and MnCo_4_-T extracted from the EXAFS fitting. 70](#_Toc225099205)

[Table S10. The parameters of Weisz-Prater criterion (*C_WP_*) for internal diffusion of all catalysts. 71](#_Toc225099206)

[Table S11. The catalytic activities of ethyl acetate oxidation over reported catalysts. 72](#_Toc225099207)

[Table S12. Structural parameters of MnCo_4_-E in O_2_/N_2_ flow at different temperatures extracted from the EXAFS fitting. 73](#_Toc225099208)

[Table S13. Structural parameters of MnCo_4_-E in ethyl acetate flow at different temperatures extracted from the EXAFS fitting. 74](#_Toc225099209)

[Supplementary References 75](#_Toc225099210)

Supplementary Experimental Details

Text S1: Catalysts Synthesis

The synthesis of MnCoSi, MnCo_2_Si, MnCo_6_Si, MnSi, CoSi, and MnCeSi followed the same procedure as that used for MnCo_4_Si in the main manuscript, with only the precursor ratios adjusted accordingly. For the MnCo_x_Si (x=1, 2, 6) catalysts, only the feeding amounts of Mn(NO_3_)_2_ and Co(NO_3_)_2_ were varied, calculated on the basis that the final oxide components are MnO_2_ and Co_3_O_4_, with Co/Mn molar ratios of x. For MnSi and CoSi, appropriate amounts of Mn(NO_3_)_2_ or Co(NO_3_)_2_ were individually added according to the desired stoichiometry. For MnCeSi, the precursor amounts were calculated assuming MnO_2_ and CeO_2_ as the final oxide, with a Mn/Ce molar ratio of 1:1. For all catalysts, the Si content was fixed at 15 wt% (calculated as SiO_2_).

The corresponding etching catalysts (MnCo-E, MnCo_2_-E, MnCo_6_-E, MnSi-E, CoSi-E, and MnCeSi-E) were prepared by an *in situ* hard-template method. Typically, 1 g of the sample was added into 50 mL of 2 mol L^-1^ NaOH solution and stirred at 80 °C for 120 min, followed by centrifuging, washing, and then drying to obtain the catalysts. The series MnCo_4_-E-X (X represents the etching time) were synthesized in an same way, with the etching time varied.

Text S2: Catalyst Characterization

X-ray diffraction (XRD) patterns were recorded on a Bruker AXS D8 Focus diffractometer that operated at 40 kV and 30 mA with a Cu target and Kα-ray irradiation (λ = 1.54178 Å). Scans were collected in the 2θ range from 10° to 80° with a rate of 5° min^-1^ to analyze the phase structure.

The N_2_ adsorption-desorption isotherms were measured at about -196 °C using a Kubo-X1000 produced by Beijing Biaode Electronic Technology Co., Ltd. Before the test, the samples were treated under vacuum at 200 °C for 5 h. The Brunauer-Emmett-Teller (BET) adsorption isotherm was used to calculate the specific surface area of the samples, and the pore size distribution was calculated using the Barret-Joyner-Halenda (BJH) method.

The Mn, Co, and Si contents of catalysts were determined by inductively coupled plasma optical emission spectrometry (ICP-OES) and was performed on an Agilent 5100 ICP OES with Dichroic Spectral Combiner (DSC) technology (Agilent Technologies, Mulgrave, Australia). Chemical composition of the samples was determined by X-ray fluorescence (XRF) using a Rigaku ZSX Primus III+ spectrometer.

The X-ray photoelectron spectroscopy (XPS) was conducted on a Thermo Scientific K-Alpha spectrometer equipped with the excitation source of Al Kα radiation which operates at 12 kV and 6 mA. Electron paramagnetic resonance (EPR) measurements were performed to monitor the active oxygen species of prepared catalysts on Bruker A300 at -173 °C. The operating parameters were as follows: central field of 3370 G, microwave frequency of 9.5 GHz, microwave power of 19.7 mW, and sweep time of 61.4 s.

H_2_-temperature programmed reduction (H_2_-TPR) and temperature programmed desorption of oxygen (O_2_-TPD) were performed on a PCA-1200 chemisorption analyzer equipped with a thermal conductivity detector (TCD). For H_2_-TPR experiment, 50 mg of the sample was placed in a quartz tube and pretreated at 200 °C for 1 h under Ar atmosphere, then cooled to 50 °C. After baseline stabilization, the catalyst was tested from 50 to 800 °C (10 ^o^C min^-1^ heating rate) in a 10% H_2_/Ar (30 mL min^-1^) flow. For tO_2_-TPD experiment, 50 mg of the sample was pretreated under He atmosphere at 200 °C for 1 h and then cool down to 50 °C. Then the gas was switched to 10 % O_2_/He (30 mL min^-1^) for 1 h. Afterwards, the sample was purged with a 30 mL min^-1^ He flow for 1 h to remove any physically adsorbed O_2_. After baseline stabilization, the catalyst was tested from 50 to 800 °C (10 °C min^-1^ heating rate) in He (30 mL min^-1^) flow.

Scanning Electron Microscope (SEM) was conducted on a ZEISS Sigma 300 to investigate the morphology of the samples. Transmission electron microscopy (TEM) and elemental mapping images were obtained using FEI Talos F200x microscope equipped with an energy dispersive spectroscopy (EDS) detector.

X-ray absorption fine structure spectroscopy (XAFS) was performed using the RapidXAFS 1M (Anhui Absorption Spectroscopy Analysis Instrument Co., Ltd.) in transmission mode. For Mn measurements, the system was operated at 10 kV and 20 mA, with a Si (440) spherically bent crystal analyzer (radius of curvature: 500 mm), In contrast, Co measurements were conducted at 20 kV and 20 mA using a Si (533) spherically bent crystal analyzer with the same radius of curvature (500 mm). XANES and EXAFS data reduction and analysis were processed by Athena and Artemis software. This instrument demonstrates its capability to provide precise structural parameters, including coordination environment and atomic distances.

Test S3: Catalytic Testing

The catalytic oxidation of ethyl acetate (EA) was conducted in a fixed-bed quartz tube reactor (6 mm id × 500 mm length). The ethyl acetate vapor (1000 ppm) was obtained by bubbling air through liquid ethyl acetate maintained at -35°C, with air as the balance gas, and the total flow rate was maintained at 40 mL min^-1^. In each experiment, 80 mg of catalyst was loaded in a quartz tube reactor under a weight hourly space velocity (WHSV) of 30,000 mL h^-1^ g^-1^. All gas lines were heated sufficiently at 100 °C to prevent the condensation of ethyl acetate in the tubes. The VOCs gas and reaction products were online detected by a gas chromatograph (GC-9790 plus, FuLi) with two FID detectors. One was for VOCs detection, and the other was for CO_2_ detection. The EA conversion (X), CO_2_ yield (Y) and CO_2_ selectivity (S) were calculated by the following equations:

$$\text{X}_{\text{EA}}\text{=}\frac{\left[ \text{C}_{\text{4}}\text{H}_{\text{8}}\text{O}_{\text{2}} \right]_{\text{in}}\text{-}\left[ \text{C}_{\text{4}}\text{H}_{\text{8}}\text{O}_{\text{2}} \right]_{\text{out}}}{\left[ \text{C}_{\text{4}}\text{H}_{\text{8}}\text{O}_{\text{2}} \right]_{\text{in}}}\text{×100\%}$$

where $\left[ \text{C}_{\text{4}}\text{H}_{\text{8}}\text{O}_{\text{2}} \right]_{\text{in}}$ and $\left[ \text{C}_{\text{4}}\text{H}_{\text{8}}\text{O}_{\text{2}} \right]_{\text{out}}$ are the inlet and outlet concentrations of EA, respectively.

$$\text{Y}_{\text{CO}_{\text{2}}}\text{=}\frac{\left[ \text{CO}_{\text{2}} \right]_{\text{out}}}{\text{4}\left[ \text{C}_{\text{4}}\text{H}_{\text{8}}\text{O}_{\text{2}} \right]_{\text{in}}}\text{×100\%}$$

where $\left[ \text{CO}_{\text{2}} \right]_{\text{out}}$ is the concentration of the outlet CO_2_.

$$\text{CO}_{\text{2}}\text{ selectivity=}\frac{\left[ \text{C}\text{O}_{\text{2}} \right]_{\text{out}}}{\text{4}\left[ \text{C}_{\text{4}}\text{H}_{\text{8}}\text{O}_{\text{2}} \right]_{\text{in}}\text{×}\text{X}_{\text{EA}}}\text{×100\%}$$

Test S4: Kinetic Studies

The kinetic studies for the oxidation of EA were performed using a fixed-bed reactor. Prior to the kinetic test, both internal and external diffusion effects were excluded by lowering the catalyst size (>100 mesh) and increasing the weight hourly space velocity (WHSV) of 120,000 mL g^-1^ h^-1^ to ensure accurate measurement of the reaction rate. The conversion of EA was kept less than 15% and the reaction rate ($\text{r}_{\text{C}_{\text{4}}\text{H}_{\text{8}}\text{O}_{\text{2}}}$, mol g^-1^ s^-1^) of EA oxidation were calculated according to the following equation:

$$\text{r}_{\text{C}_{\text{4}}\text{H}_{\text{8}}\text{O}_{\text{2}}}\text{=}\frac{\text{X}_{\text{C}_{\text{4}}\text{H}_{\text{8}}\text{O}_{\text{2}}}\text{⋅}\text{V}_{\text{C}_{\text{4}}\text{H}_{\text{8}}\text{O}_{\text{2}}}}{\text{g}_{\text{cat}}}$$

where $\text{V}_{\text{C}_{\text{4}}\text{H}_{\text{8}}\text{O}_{\text{2}}}$ is the gas flow rate of EA (mol·s^-1^), $\text{g}_{\text{cat}}$ is the weight of catalyst (g).

$$\ln\text{r}_{\text{C}_{\text{4}}\text{H}_{\text{8}}\text{O}_{\text{2}}}\text{=-}\frac{\text{E}_{\text{a}}}{\text{RT}}\text{+C}$$

where R is the ideal gas constant (8.314 J mol^-1^ K^-1^), T is the reaction temperature (K), and C is a constant. $\text{E}_{\text{a}}$ could be obtained from the slope of the resulting linear plot of $\ln\text{r}_{\text{C}_{\text{4}}\text{H}_{\text{8}}\text{O}_{\text{2}}}$ versus 1/T.

Test S5: DFT Calculations

All calculations were based on the first principles of Density Functional Theory (DFT), using the Vienna Ab-initio Simulation Package (VASP). ^1-3^ The projected augmented wave method was employed for the processing of the Kohn-Sham equation.^4,5^ The generalized gradient approximation and Perdew-Burke-Ernzerh of exchange-correlation functional were adopted.^6^ A kinetic energy cutoff of 450 eV was used in the adsorption calculations. The Brillouin zone integrals were performed using a gamma-centered 1 × 1 × 1 grid. The entire adsorbed molecular model relaxation technique was used for geometry optimization and a vacuum layer with a thickness of 20 Å was added in the Z-direction to simulate the surface and ensure that the reaction is not affected by the next layer. The electronic and geometric convergence criteria were set to 10^−5^ eV and 0.02 eV·Å^−1^, respectively. Adsorption is allowed on only one side of the exposed surfaces, with the dipole moment corrected accordingly in the z-direction. All the adsorption reaction is carried out on the top of active center sites. The formation energy of oxygen vacancy was defined as E_OV_=E_slab+OV_+$\frac{\text{1}}{\text{2}}$E$\text{O}_{\text{2}}$-E_slab_, where E_slab+OV_, E$\text{O}_{\text{2}}$, and E_slab_ represent the energies of the surface with oxygen defect, gaseous oxygen, and the perfect slab surface. The adsorption energy (E_ads_) was calculated as E_ads_=E_adsorbed state_-E_slab_-E_molecule_. Here, E_adsorbed state_ is the total energy of the catalyst with adsorbed molecules, and E_molecule_ is the gaseous adsorbate molecules such as ethyl acetate and oxygen.

Test S6: Detailed *in situ* experimental tests

*In situ* DRIFTS measurements over the synthesized samples were obtained using a Thermo Scientific Nicolet IS50 equipped with a mercury-cadmium-telluride (MCT) detector and a Harrick DRIFTS cell. The liquid nitrogen was used to cool the detector. The sample was loaded into cell and pretreated with nitrogen at temperature of 200 °C for 60 min, and then cool down to 30 °C. Background measurements were taken over the original catalyst surface under N_2_ flow. The spectra were acquired with a scanning time of 11 seconds each scan, using a spectral resolution of 4 cm^-1^. In this work, three set of *in situ* DRIFTS experiments were used to explore the intermediates species and reaction pathways of ethyl acetate oxidation.

ⅰ) Ethyl acetate temperature-programmed oxidation

Following pretreatment with N_2_ at 200 °C for 60 min, the gas mixture was change to 1000 ppm ethyl acetate in 20% O_2_/N_2_ (balanced with N_2_). The temperature was programmed with a 10 °C min^-1^ heating ramp, maintaining each target temperature (at 20 °C intervals) for 13 min to ensure stable spectroscopic readings.

ⅱ) Ethyl acetate temperature-programmed desorption

Following pretreatment with N_2_ at 200 °C for 60 min, the temperature was lowered to 30 °C and the gas mixture was change to 1000 ppm ethyl acetate balanced with N_2_. After adsorbed and stabilization for 30 min, switch the gas to N_2_ and continue purging for 10 min until stabilization. Subsequently, increase the temperature to 250 °C at a rate of 10 °C min^-1^, with a 20 °C increment at each stage, and collect the spectrum after maintaining stability for 13 min.

ⅲ) Adsorption and total oxidation at 170 °C

Following pretreatment with N_2_ at 200 °C for 60 min, the temperature was cooled to 170 °C and gas flow was changed to 1000 ppm ethyl acetate balanced with N_2_ for 30 min of absorption. After adsorbed for 30 min, the gas replaced with 20% O_2_/N_2_ for deep oxidation.

For *in situ* XAFS measurements, 40 mg of the catalyst was pressed into a slice. Gases were supplied via rotary flow controllers and the total gas flow was set to 60 mL min^-1^. The catalyst was pre-treated in N_2_ for 30 min at 200 ^o^C and then cooled to 30 ^o^C. After that, ethyl acetate (balanced with 20 % O_2_/N_2_) was continuously introduced for 30 min. And then, the sample was heated at a rate of 10 °C·min^-1^ to 50, 100, 150, and 200 ^o^C, respectively. XANES spectra were collected during the heating process, and EXAFS spectra acquired after stabilization at each corresponding temperature. For the oxygen activation experiments, the same pretreatment condition was conducted without EA feed. The other conditions remained consistent with described above.

Supplementary Figures


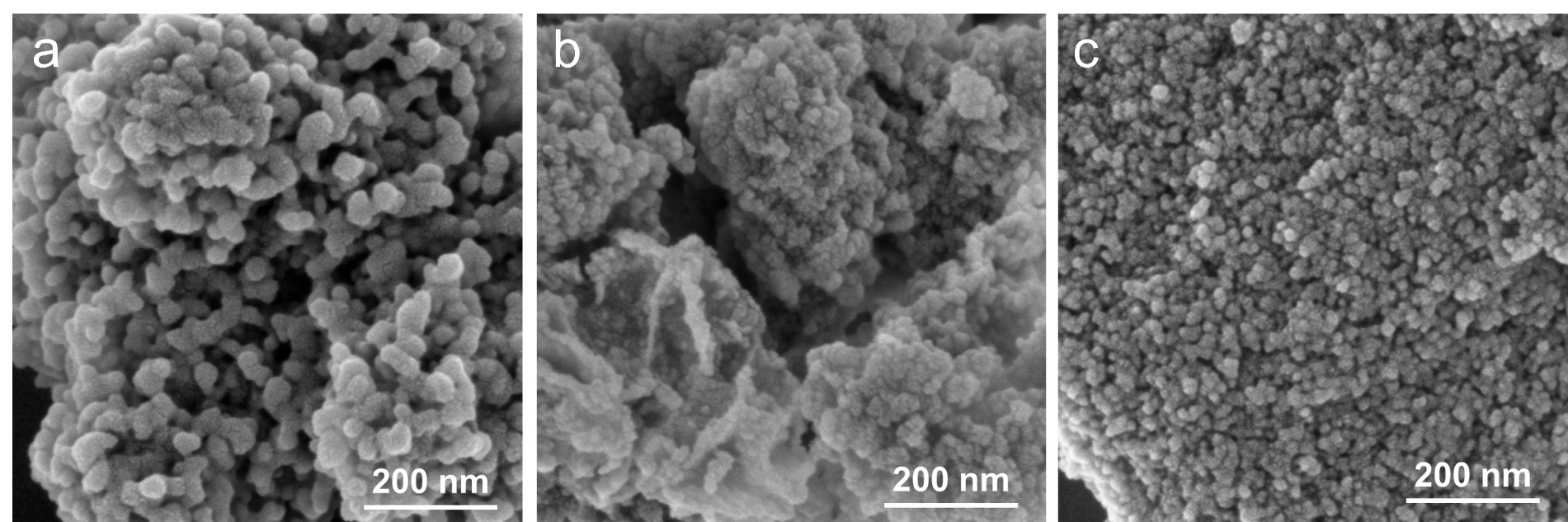


**Figure S1.** SEM images of and (a) MnCo_4_-E, (b) MnCo_4_Si and (c) MnCo_4_-T.


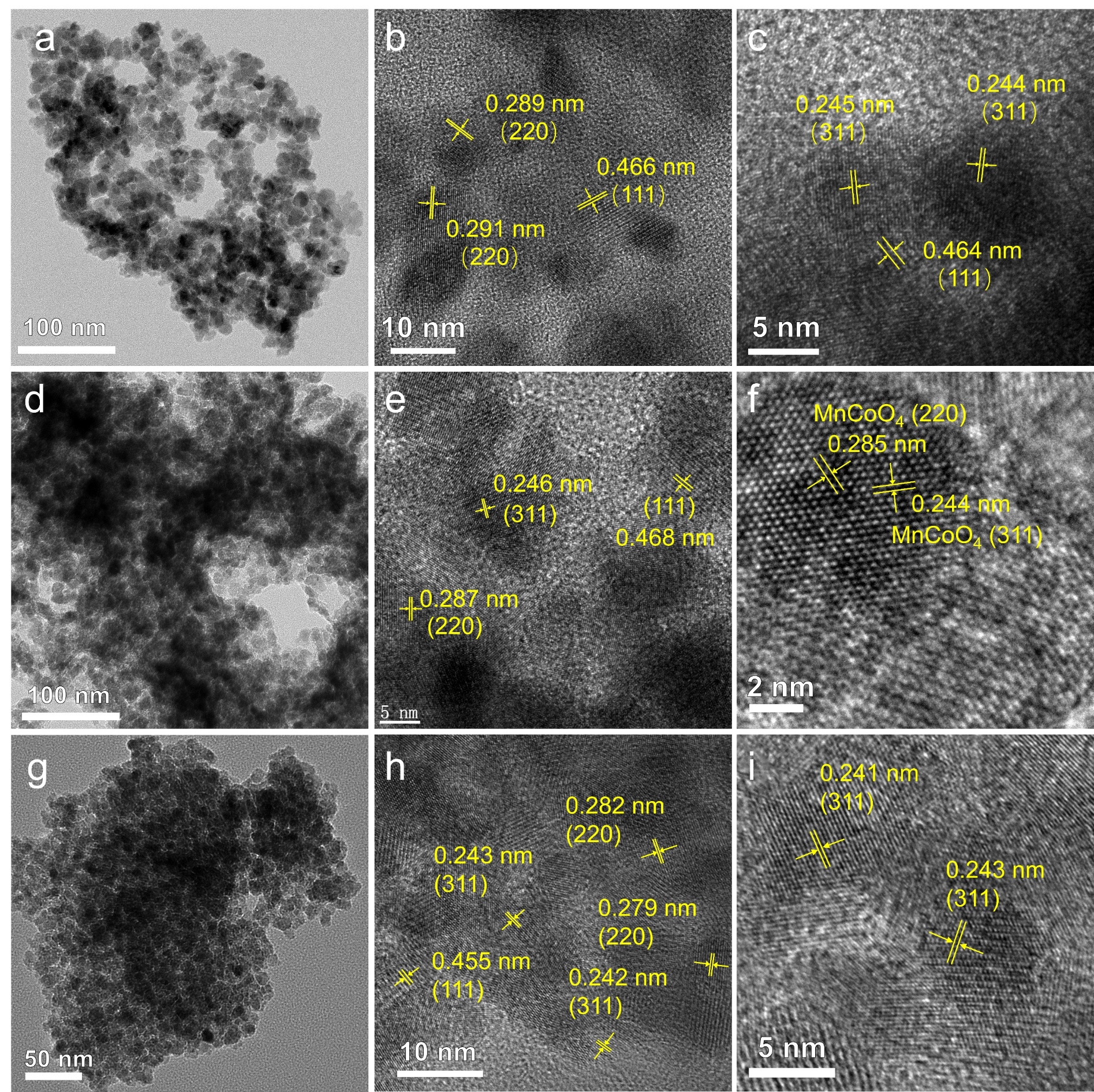


Figure S2. TEM images of (a-c) MnCo_4_-E, (d-f) MnCo_4_Si, and (g-i) MnCo_4_-T.


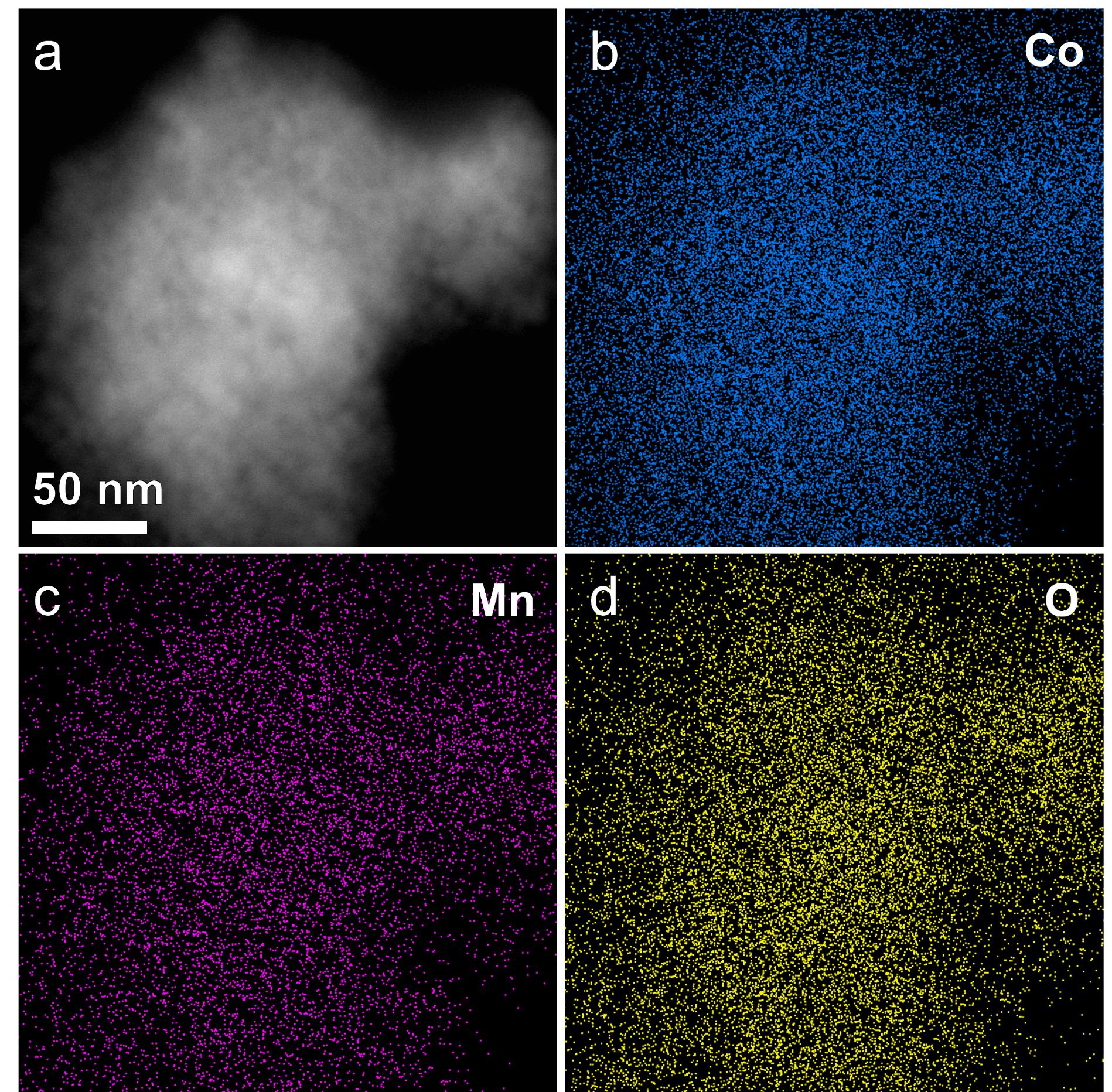


Figure S3. (a) STEM image and (b-d) elemental mappings of MnCo_4_-T.


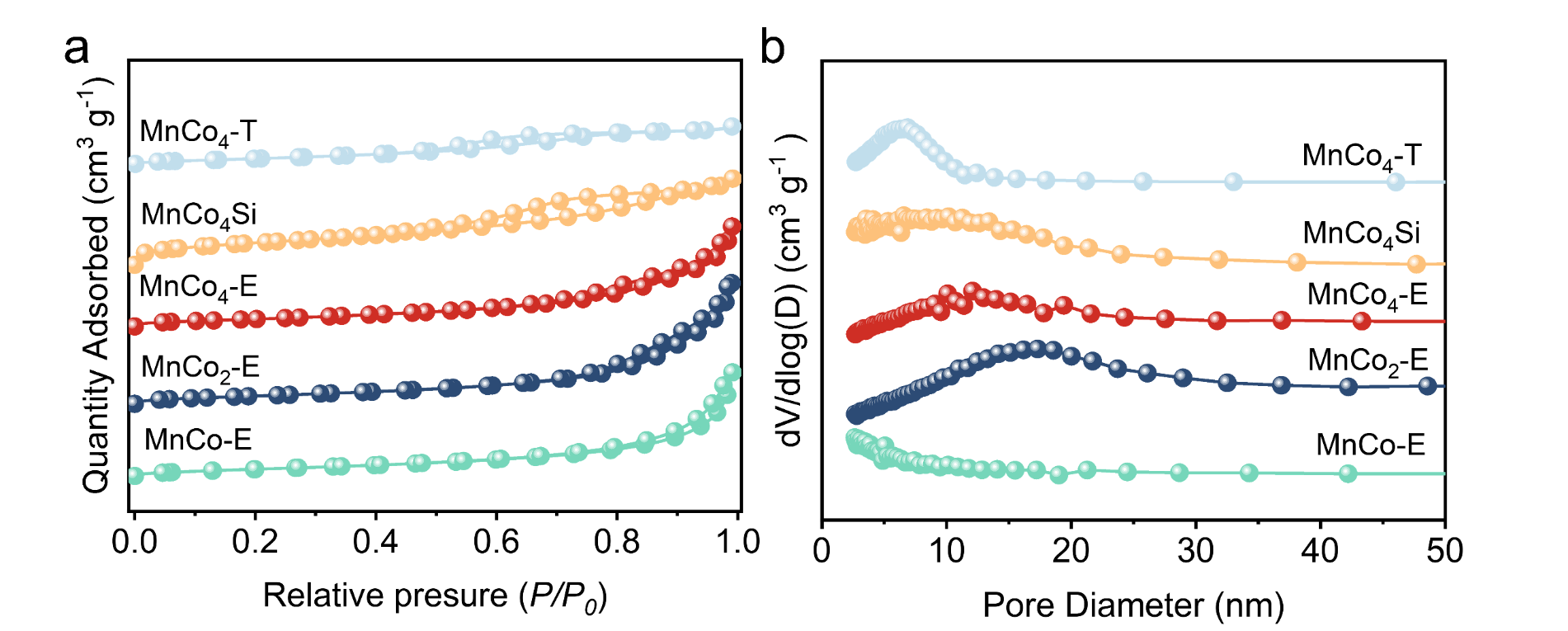


Figure S4. (a) N_2_ adsorption-desorption isotherms and (b) pore size distribution profiles of MnCo_4_Si, MnCo_4_-T, MnCo_4_-E, and related catalysts.


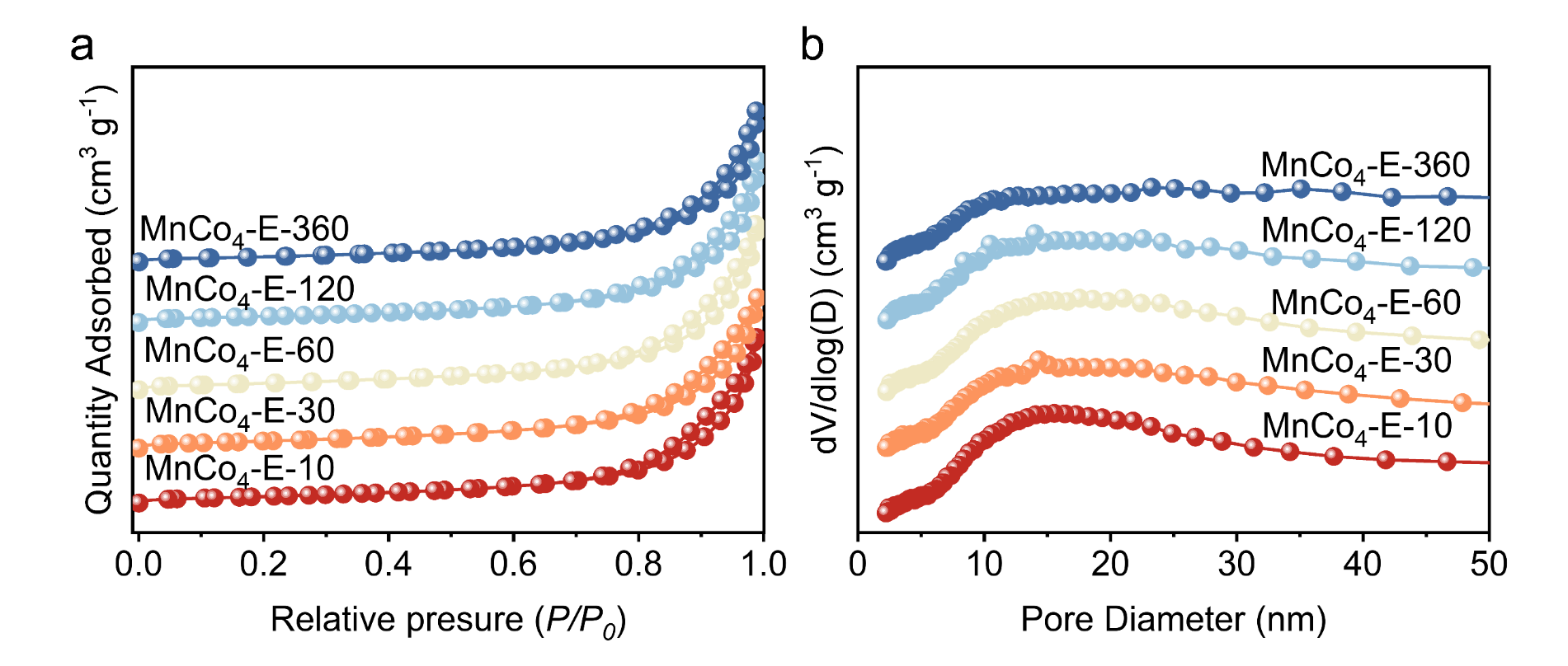


Figure S5. (a) N_2_ adsorption-desorption isotherms and (b) pore size distribution profiles of MnCo_4_-E with different alkali-treated time.


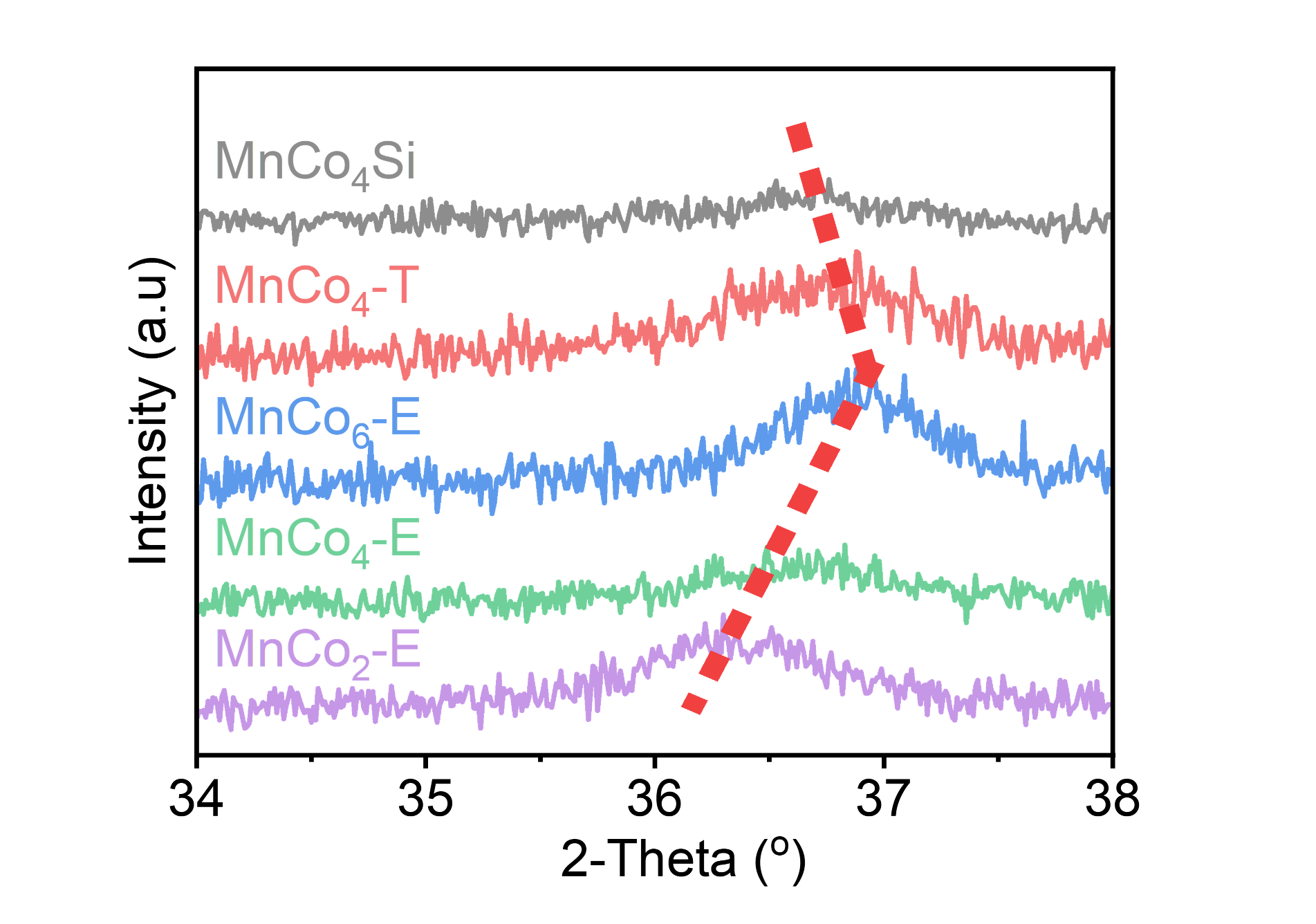


Figure S6. Lattice diffraction peak of the MnCo spinel (311) plane of the MnCo_4_-E and related catalysts.


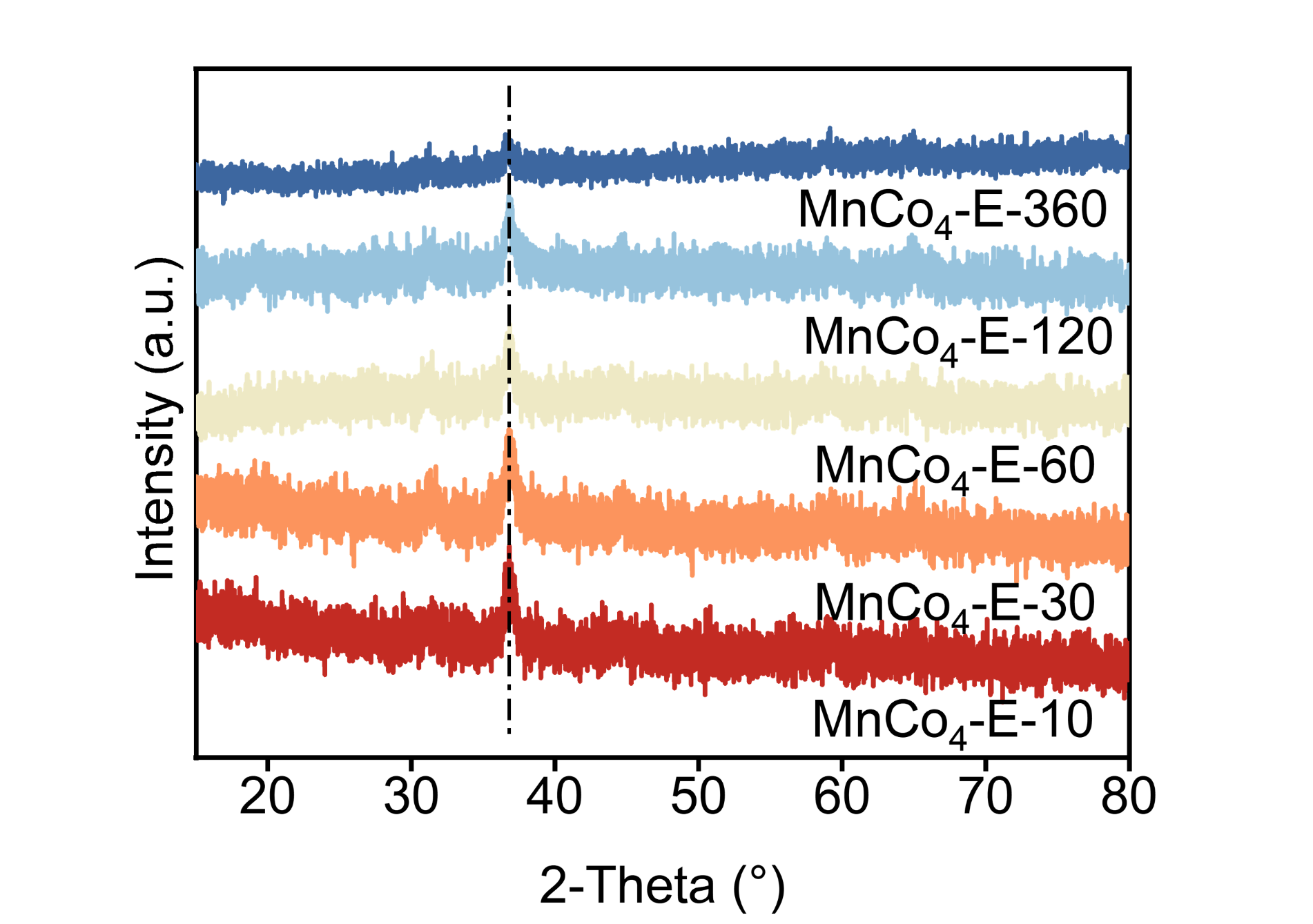


Figure S7. The XRD patterns of MnCo_4_-E with different alkali-treated time.


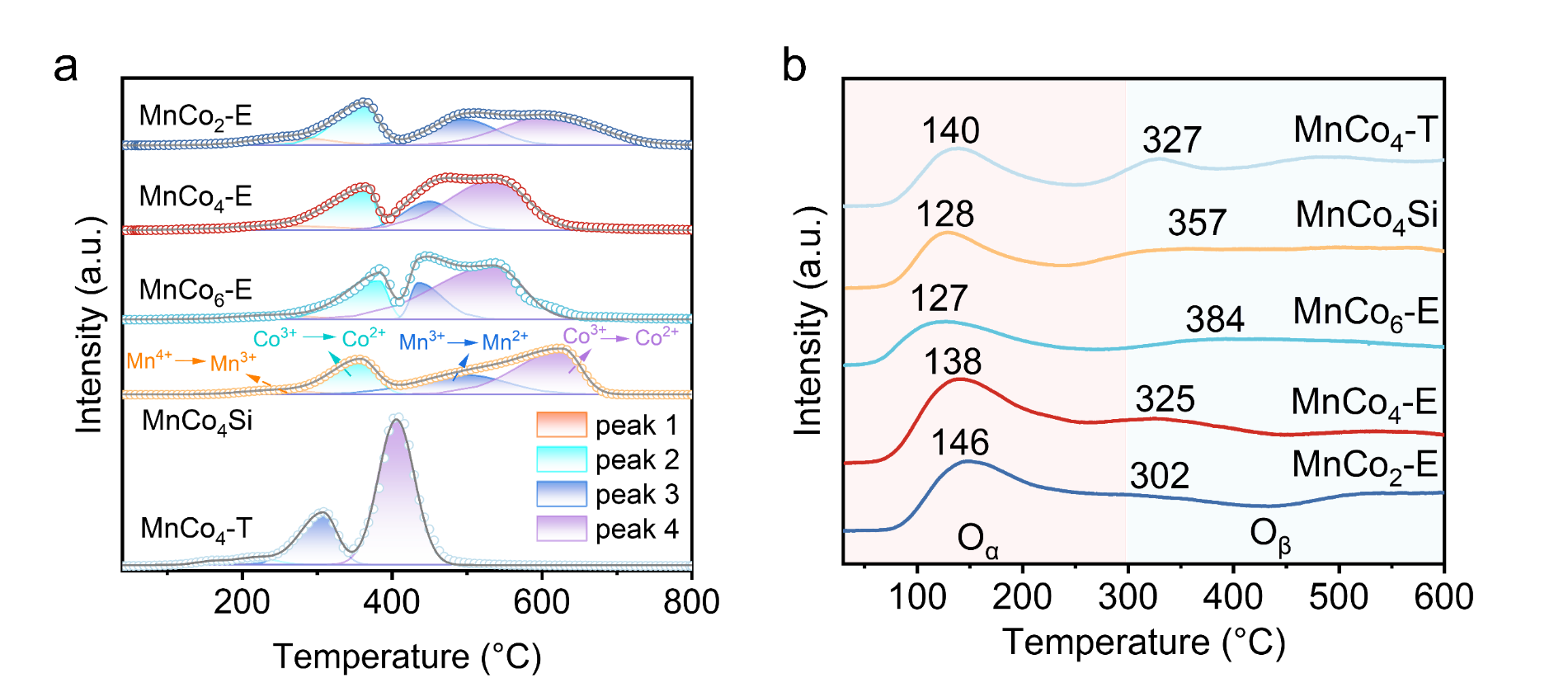


Figure S8. (a) H_2_-TPR and (b) O_2_-TPD profiles of MnCo_4_-E and related catalysts.


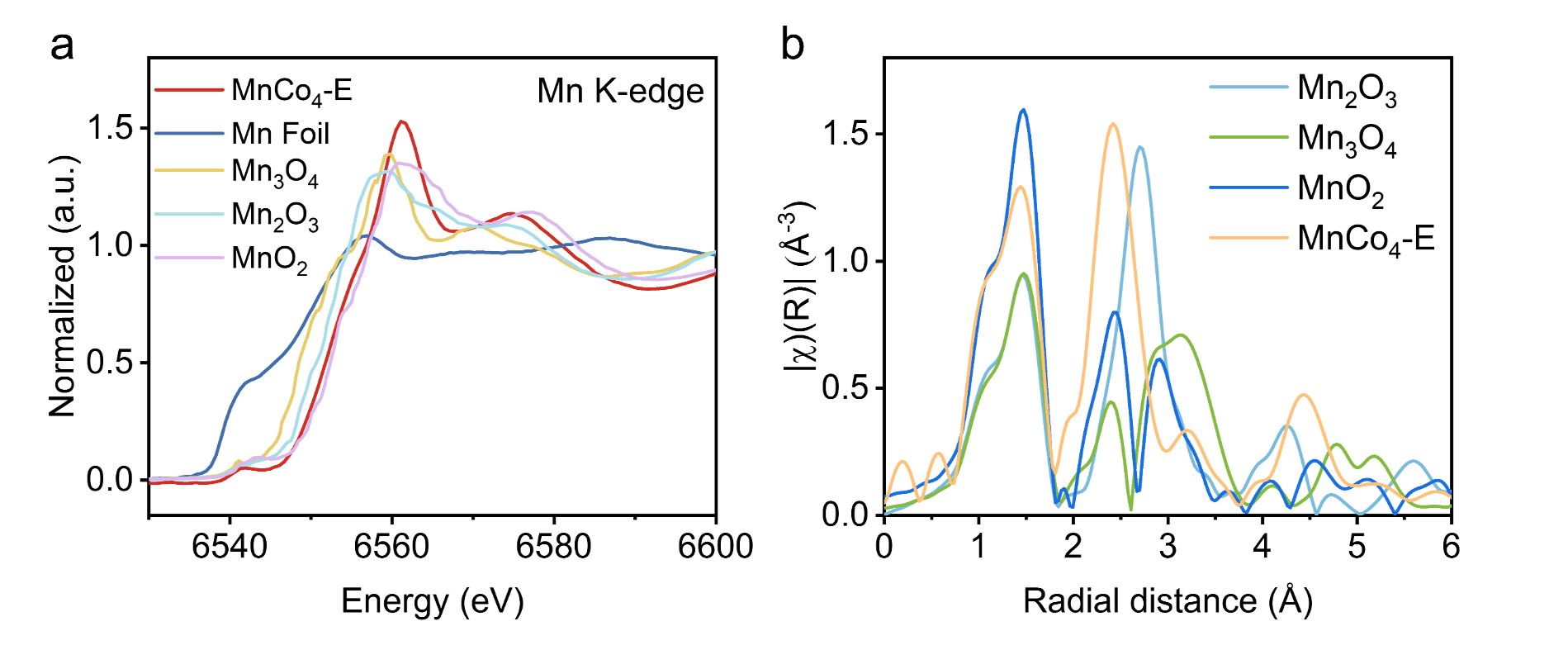


Figure S9. Normalized Mn K-edge XANES spectra of MnCo_4_-E and standard substance.


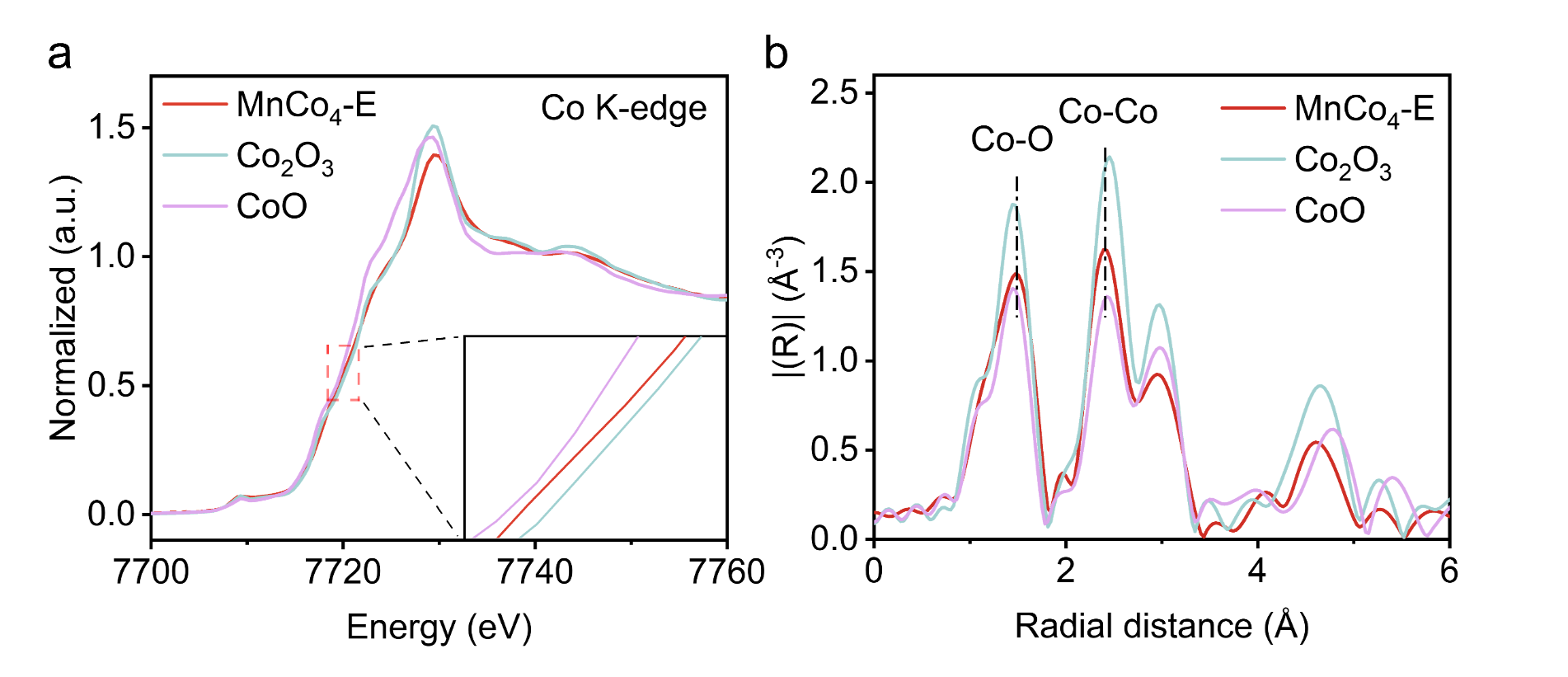


Figure S10. Normalized Co K-edge XANES spectra of MnCo_4_-E and standard substance.


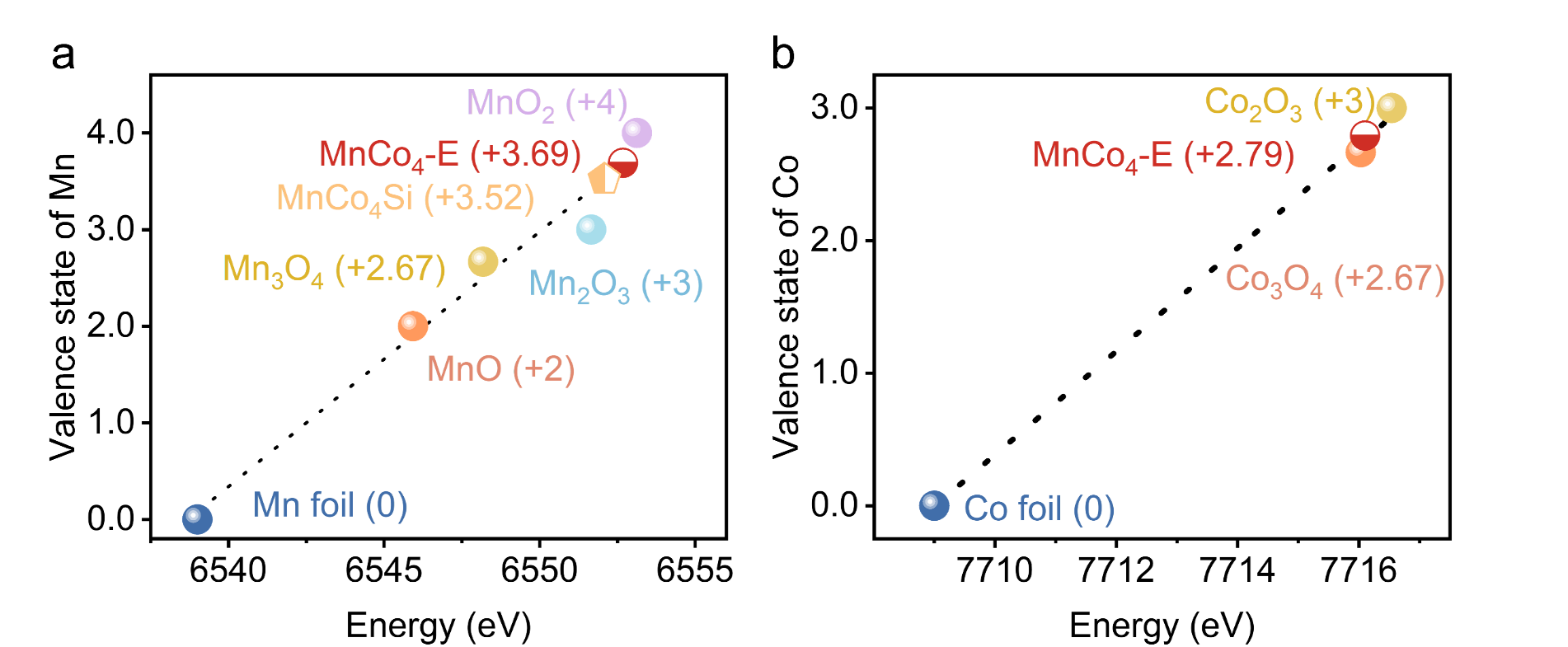


Figure S11. The valence states of (a) Mn and (b) Co in different substances.


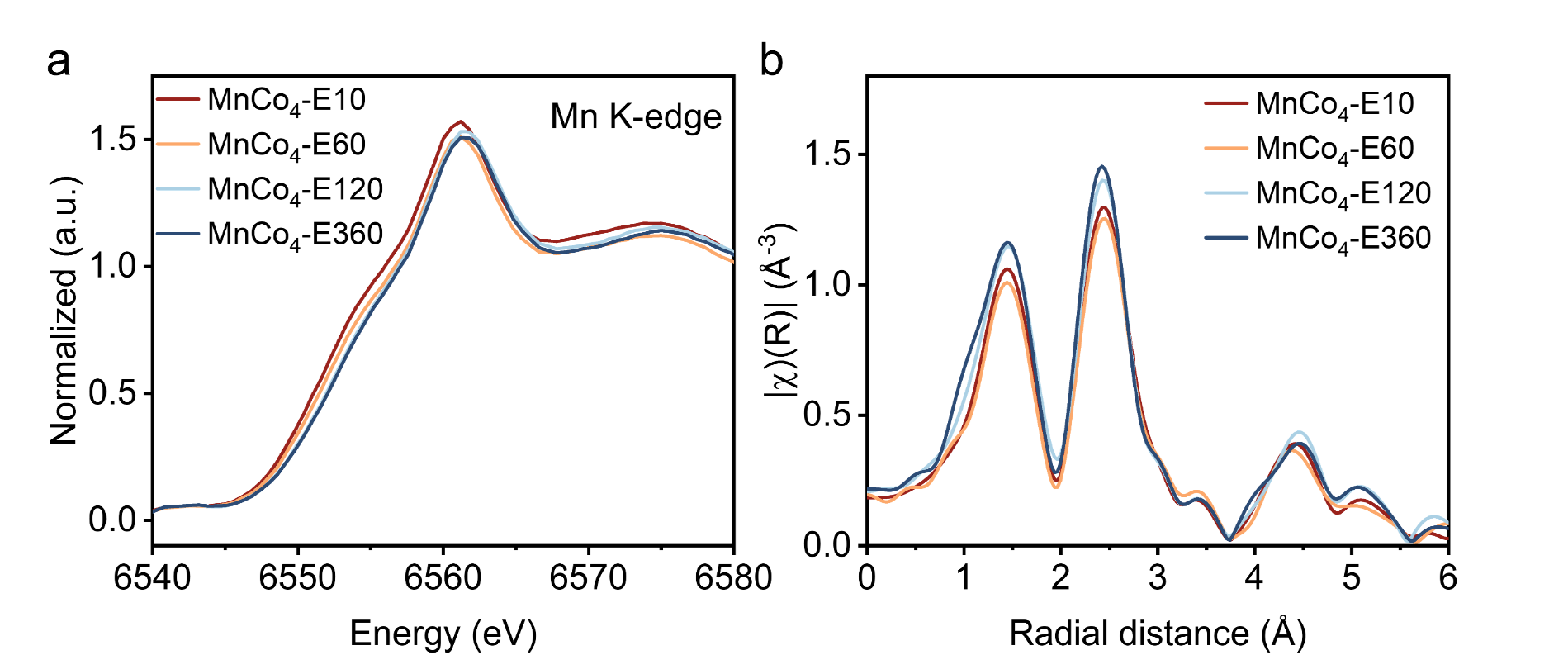


Figure S12. Fourier-transform Mn K-edge EXAFS spectra of MnCo_4_-E with different alkali-treated time.


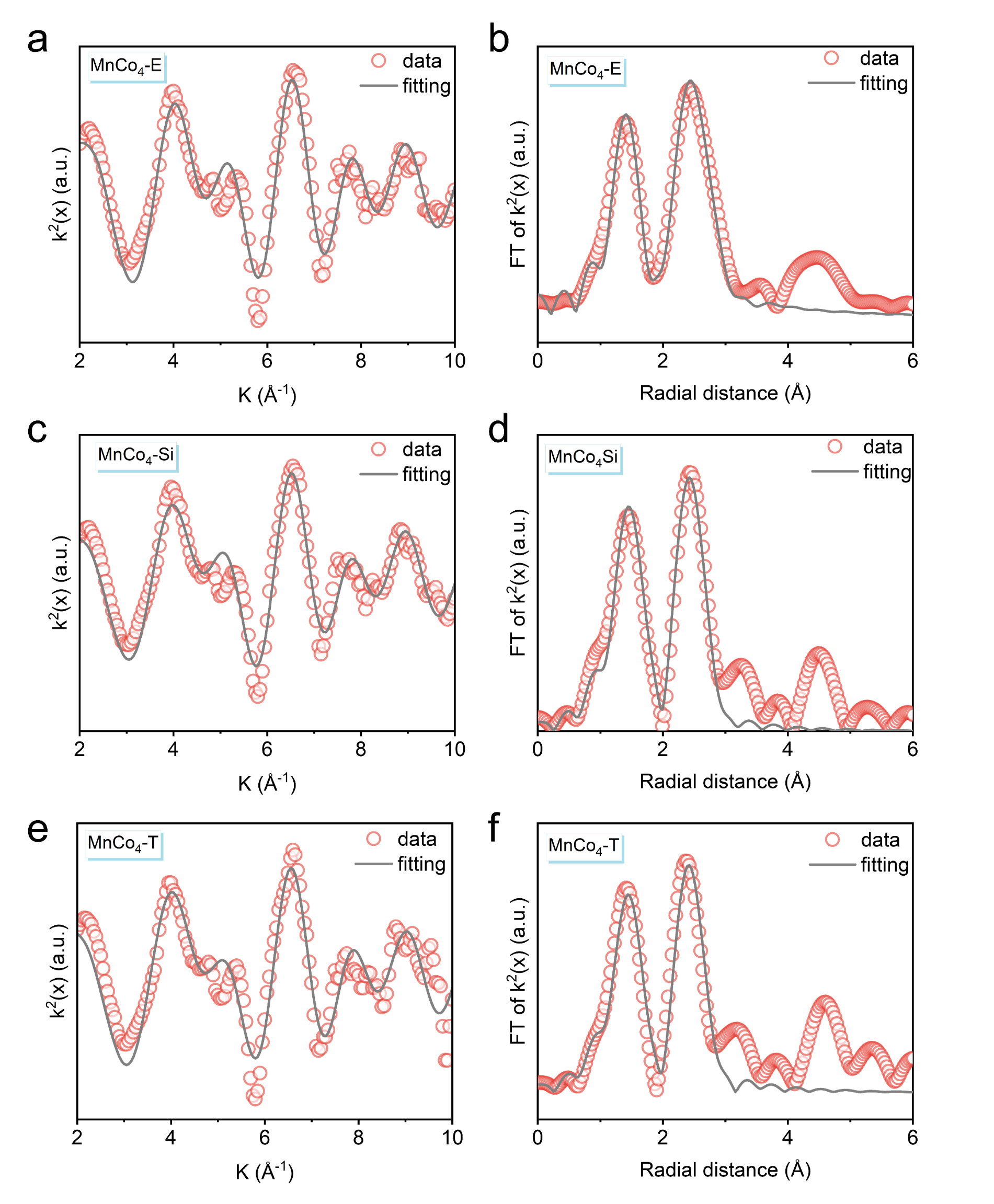


Figure S13. Mn K-edge EXAFS fitting curves of (a-b) MnCo_4_-E, (b-c) MnCo_4_Si, and (e-f) MnCo_4_-T sample in K and R spaces.


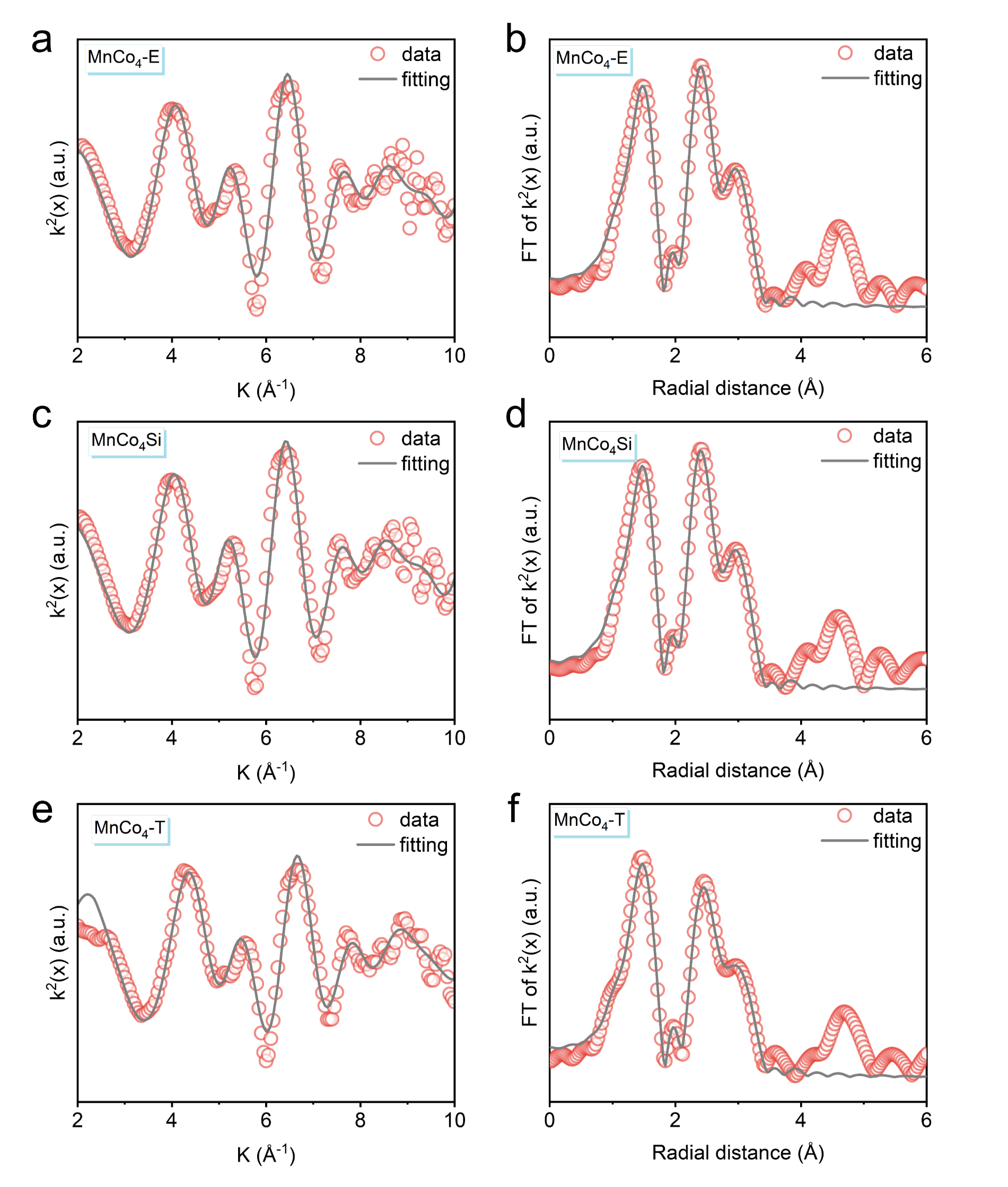


Figure S14. Co K-edge EXAFS fitting curves of (a-b) MnCo_4_-E, (b-c) MnCo_4_Si, and (e-f) MnCo_4_-T sample in K and R spaces.


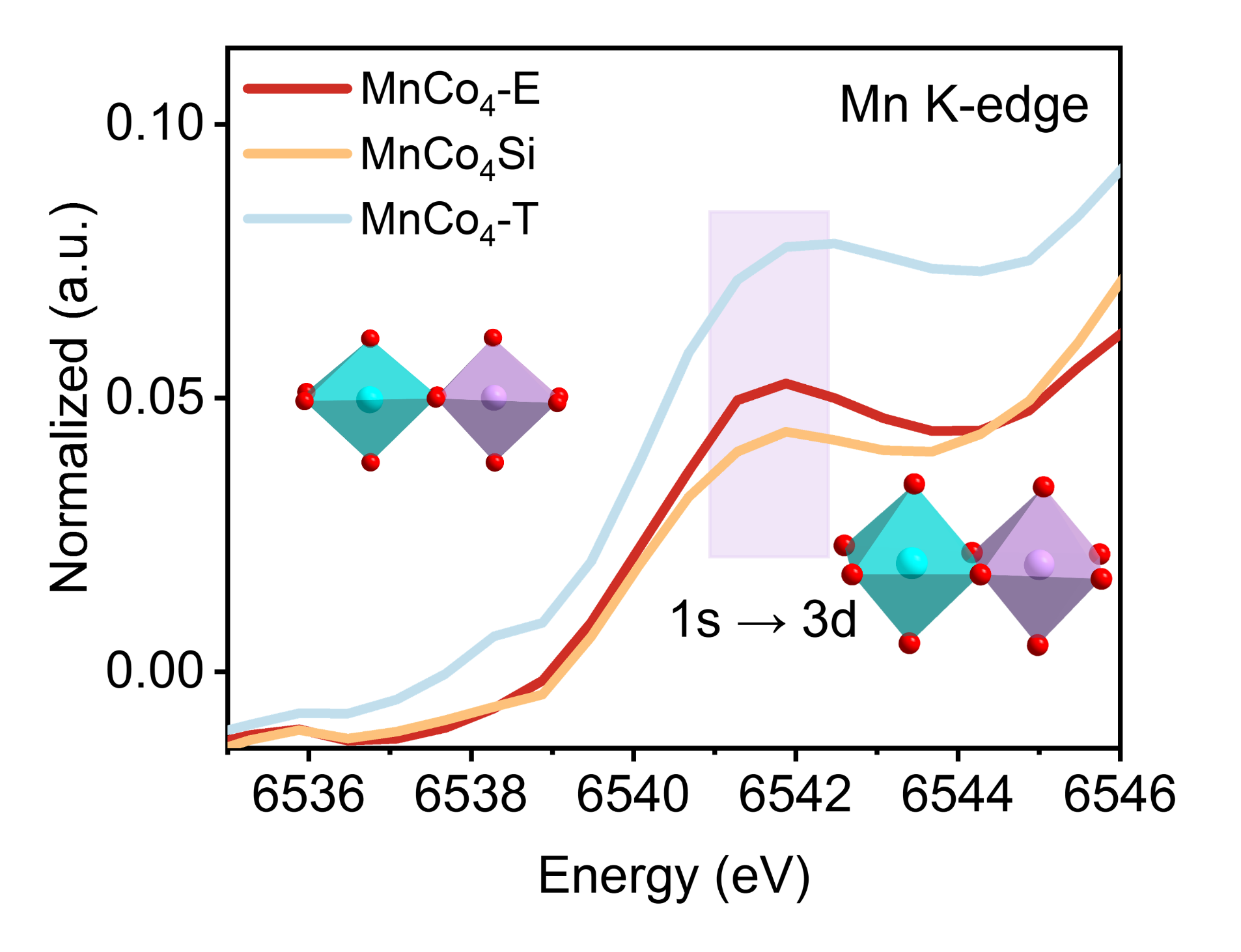


Figure S15. Pre-edge spectra of normalized Mn K-edge XANES for MnCo_4_-E and reference catalysts.


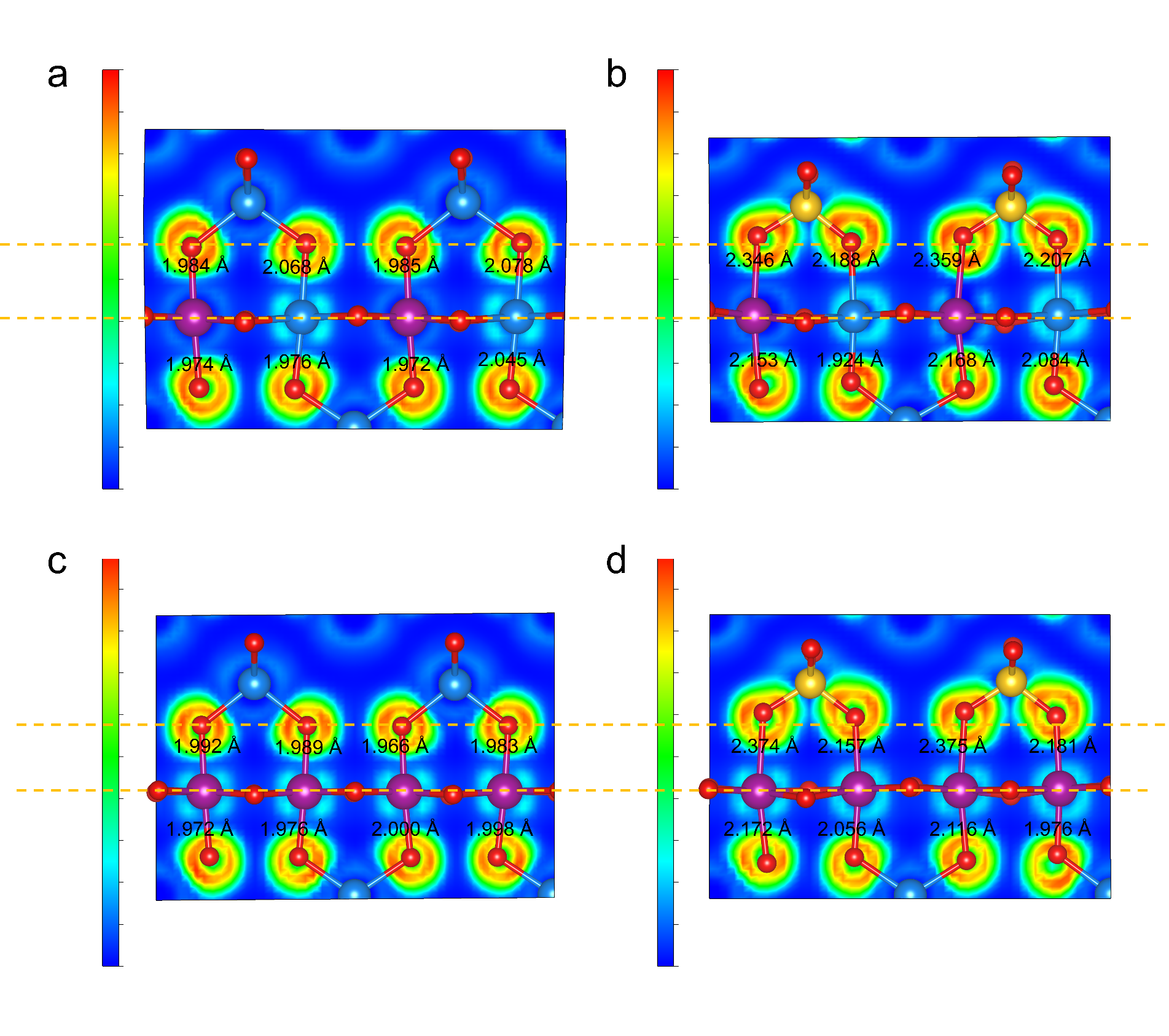


Figure S16. The ELF images of MnCo spinel with Si atoms substituted Co_Td_ sites.


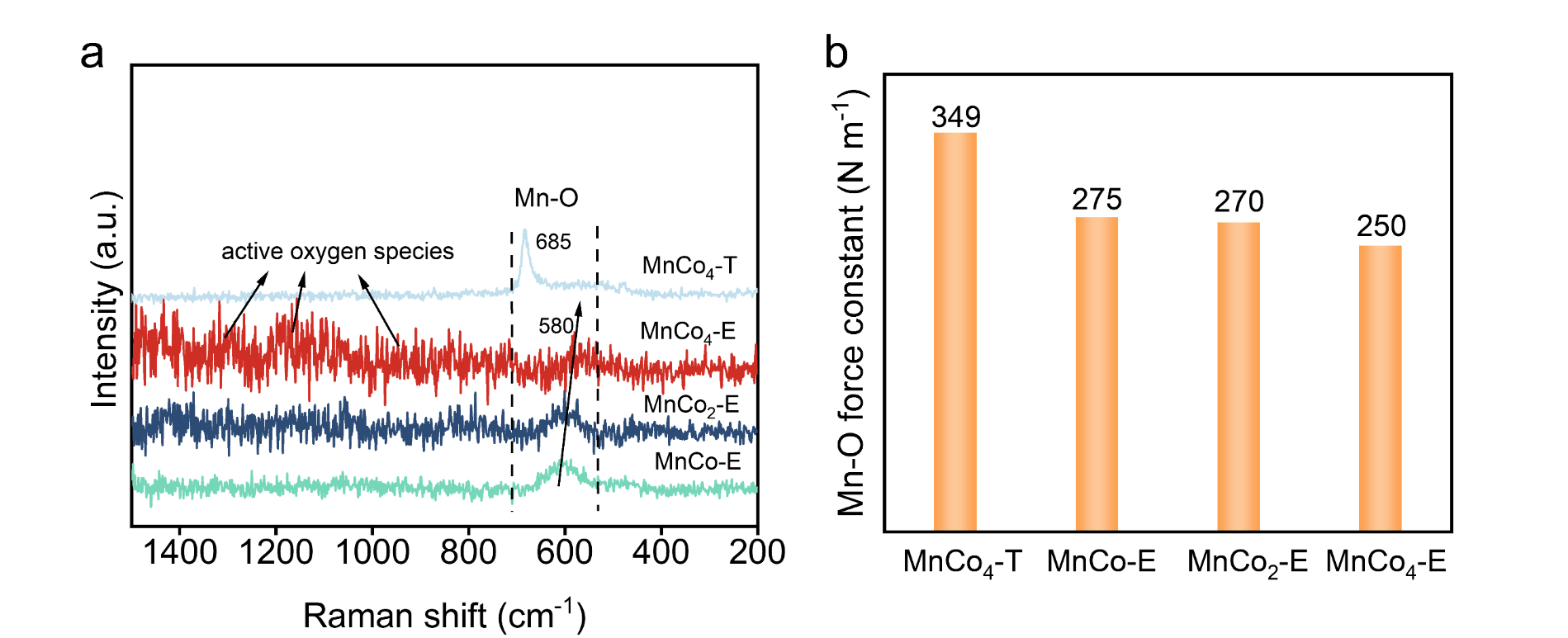


Figure S17. (a) Raman spectra and (b) Mn-O force constant of prepared catalysts.


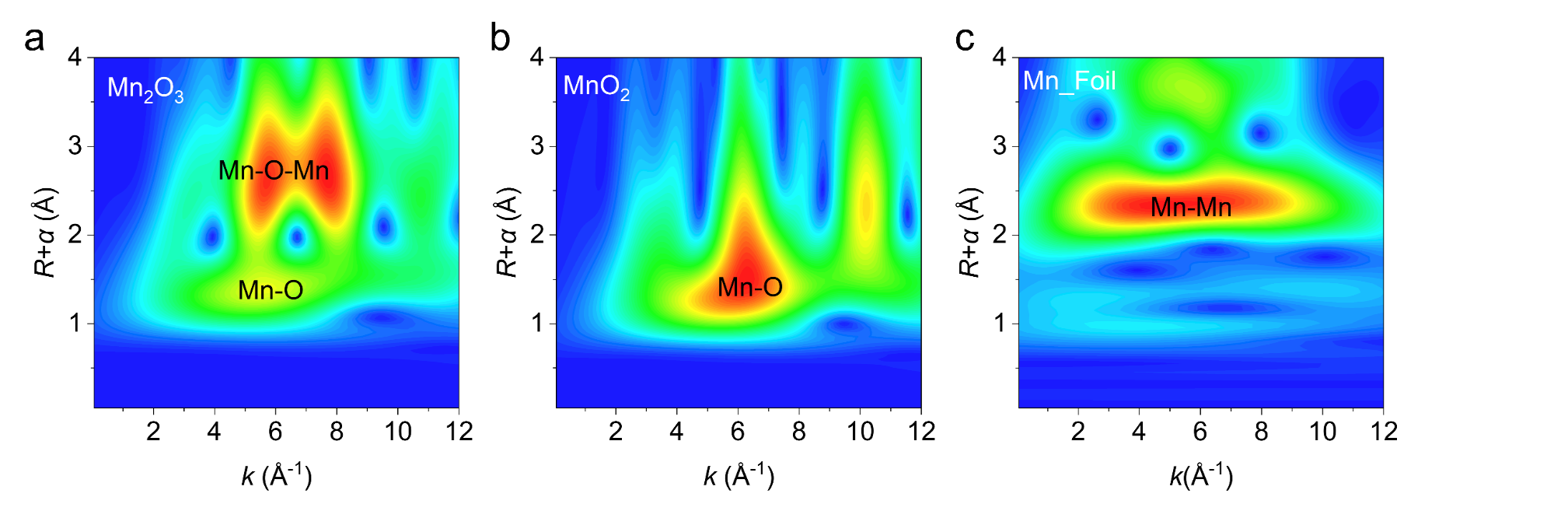


Figure S18. Wavelet transform of the Mn K-edge EXAFS spectra of Mn oxides reference samples.


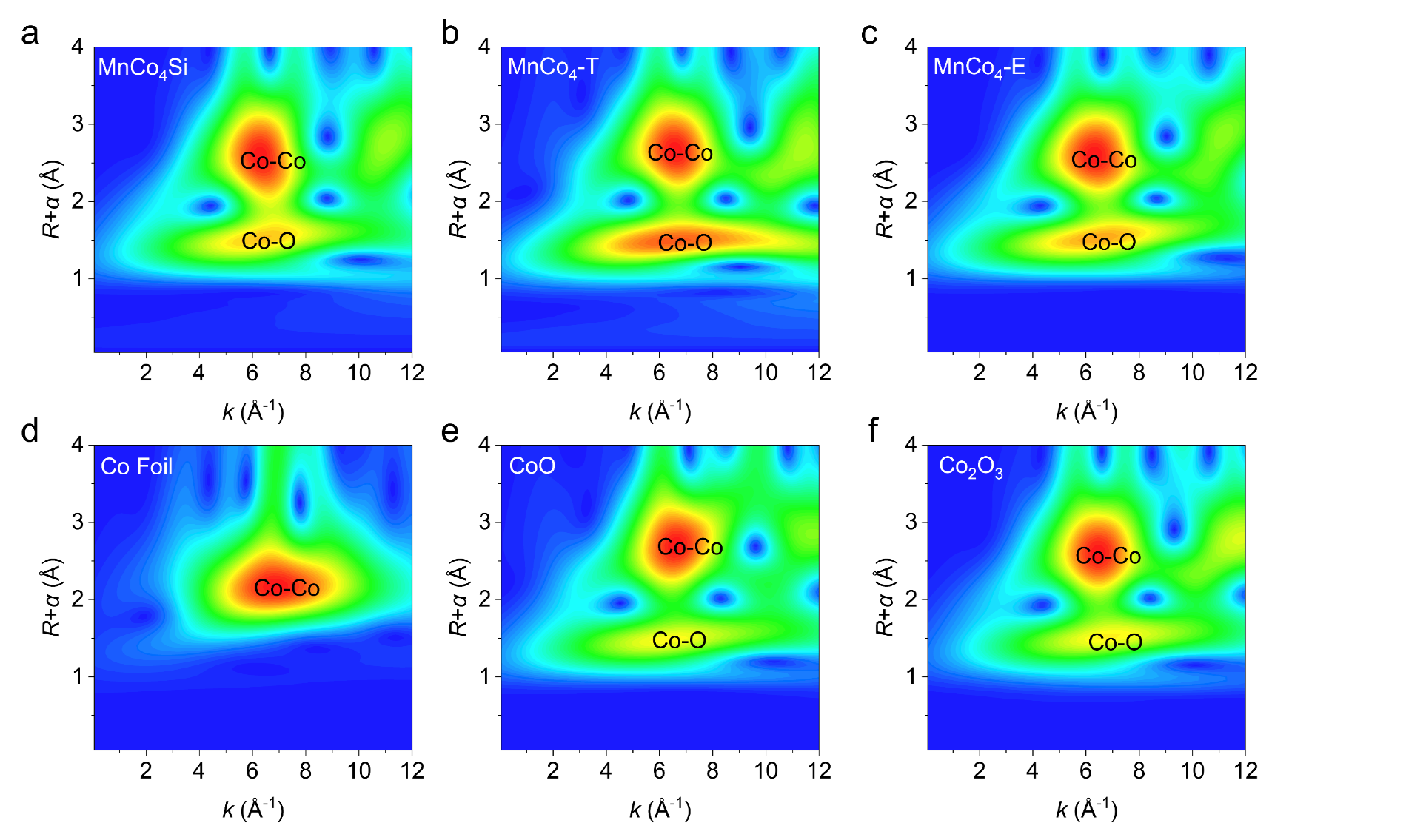


Figure S19. Wavelet transform of the Co K-edge EXAFS spectra of MnCo_4_-E and reference samples.


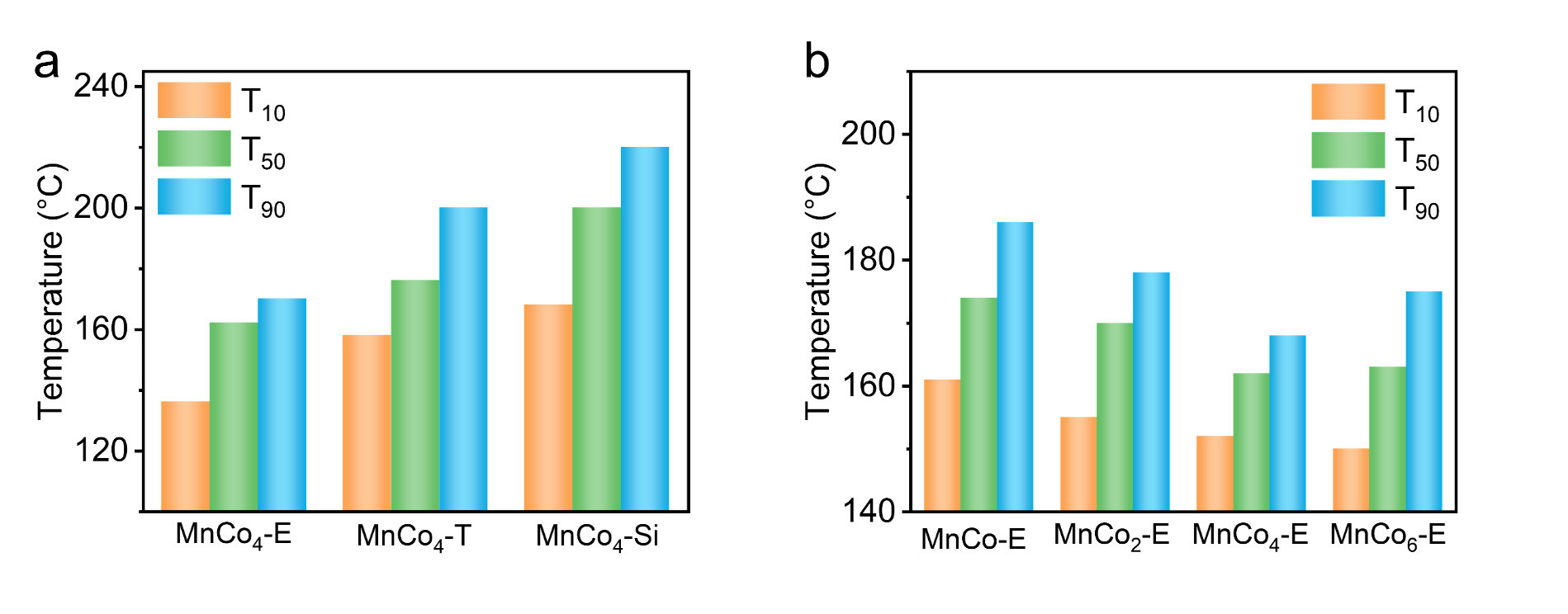


Figure S20. The activity (a) T_10_, T_50_ and T_90_ of MnCo_4_-E and related catalysts, (b) Ethyl acetate conversation of catalysts with different Mn/Co ratio.


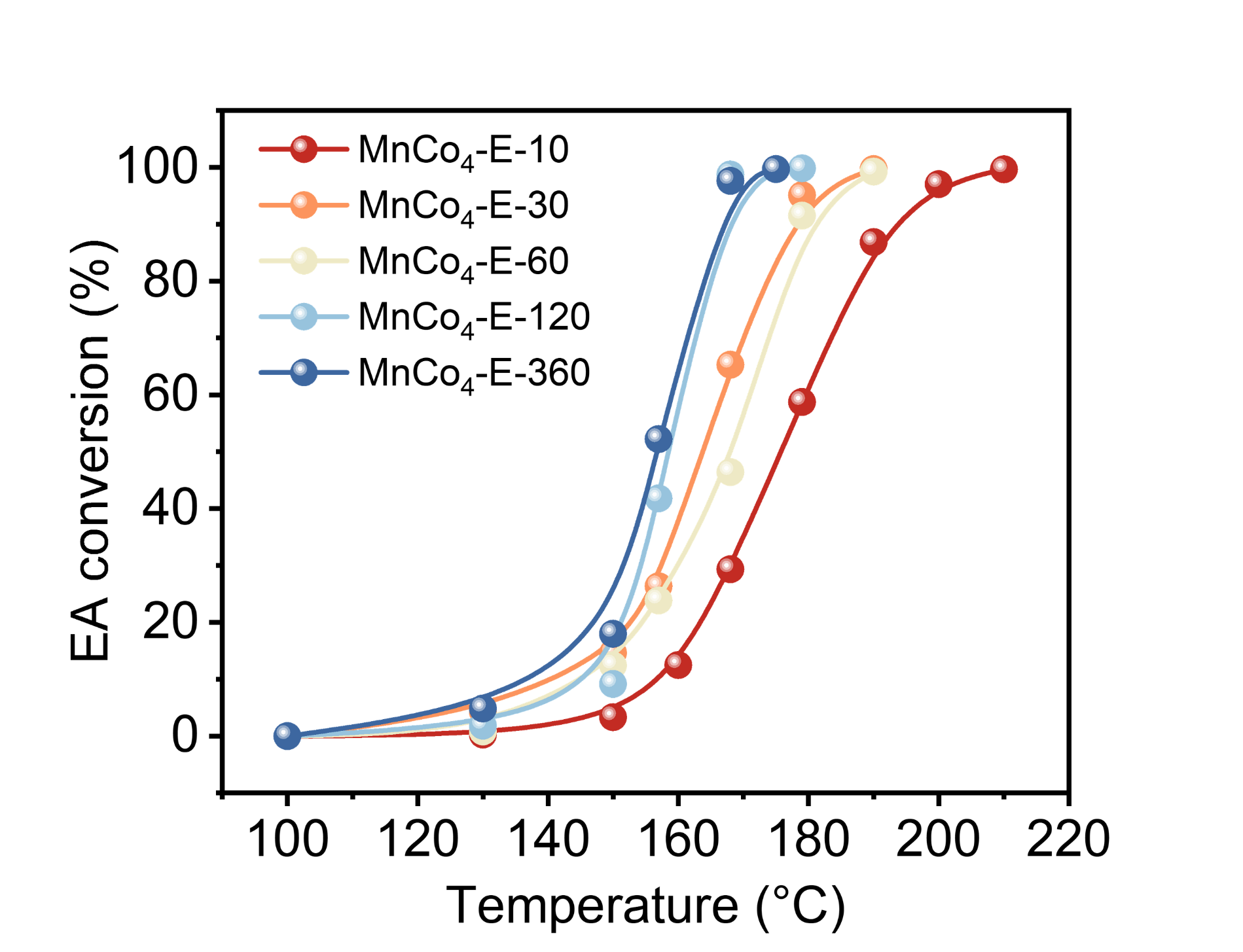


Figure S21. The ethyl acetate conversion of MnCo_4_-E with different alkali-treated time.


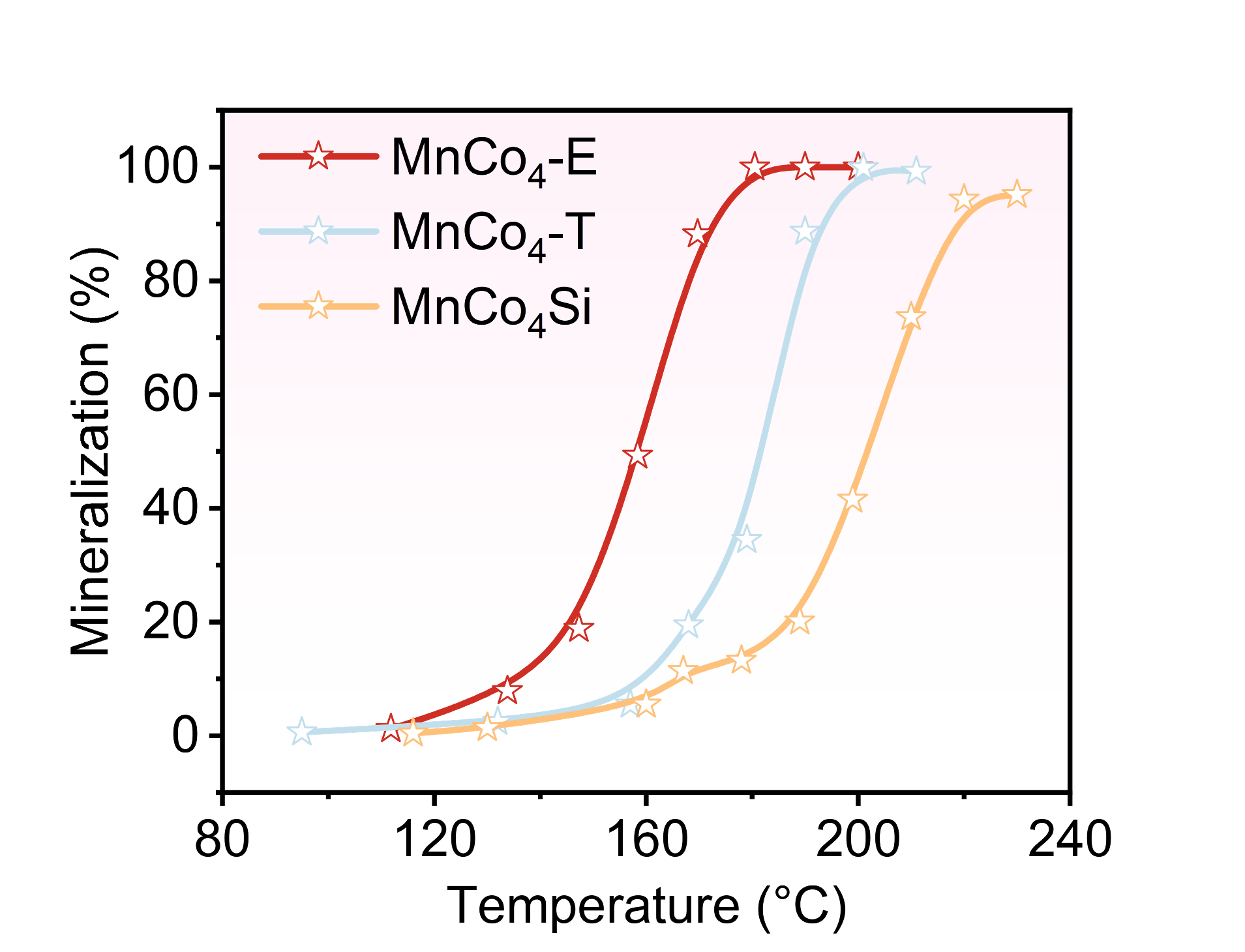


Figure S22. Plot of ethyl acetate mineralization versus temperature over various catalysts.


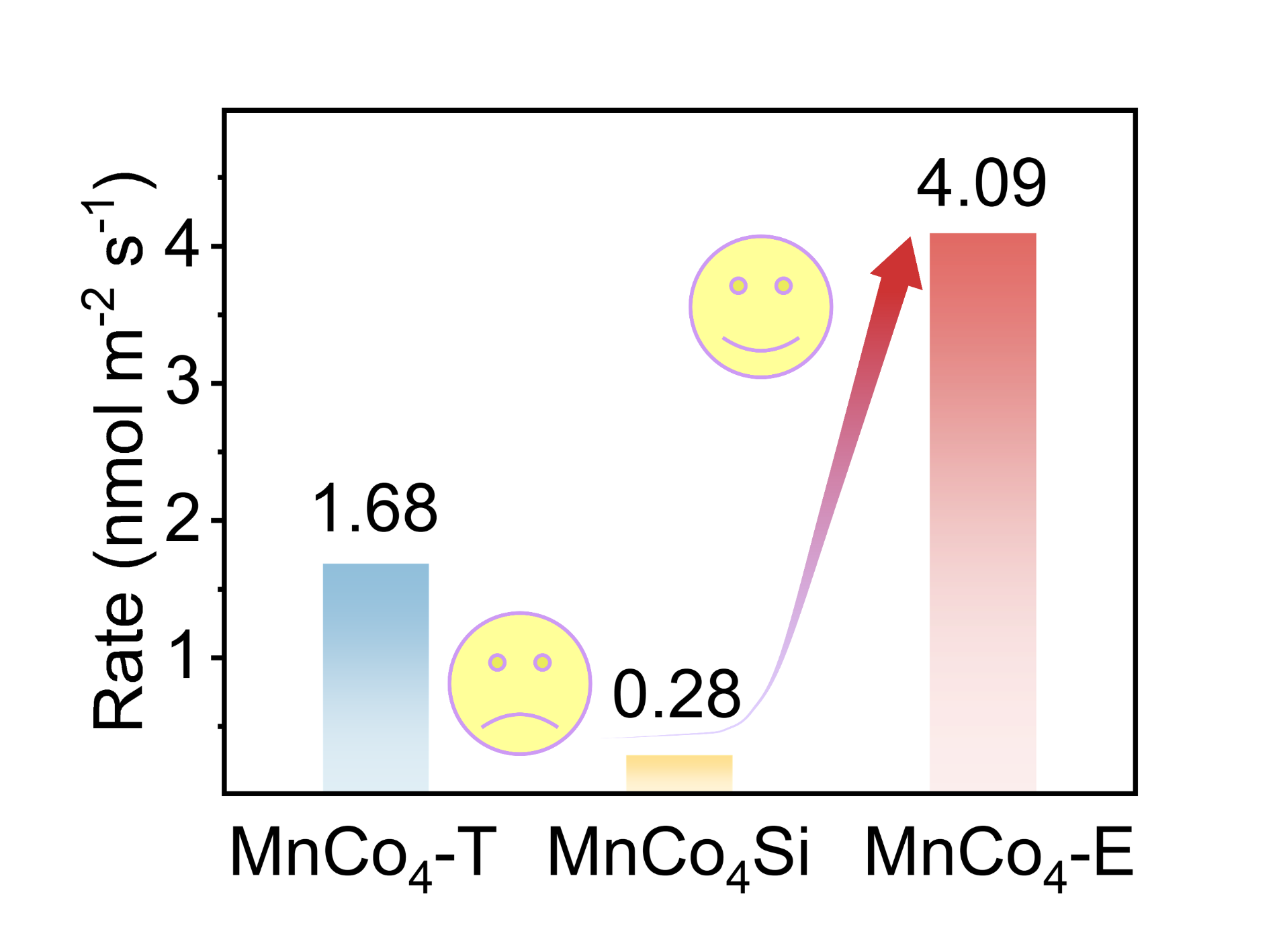


Figure S23. Reaction rates of series catalysts at 168 °C (excluding the effect of specific surface area).


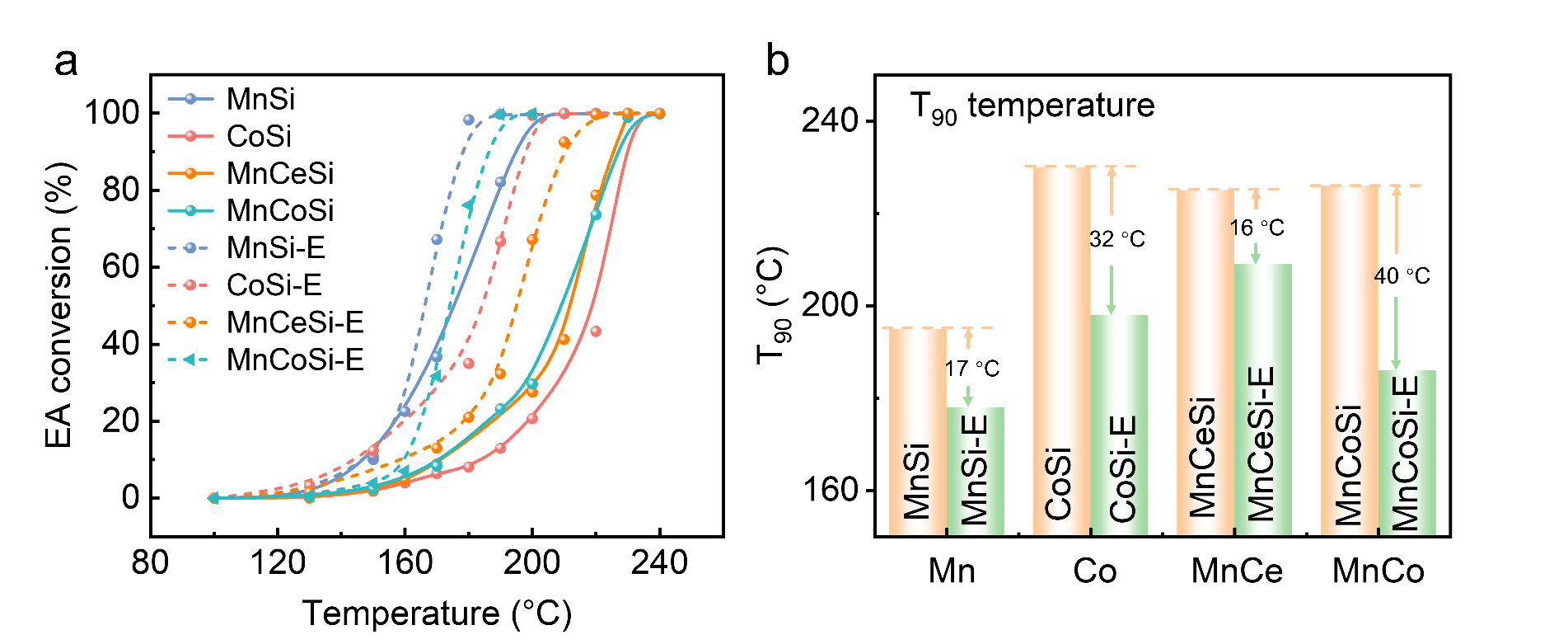


Figure S24. (a) Catalytic performance and (b) T_90_ temperature of different metal oxide prepared by *in situ* hard-template toward ethyl acetate.


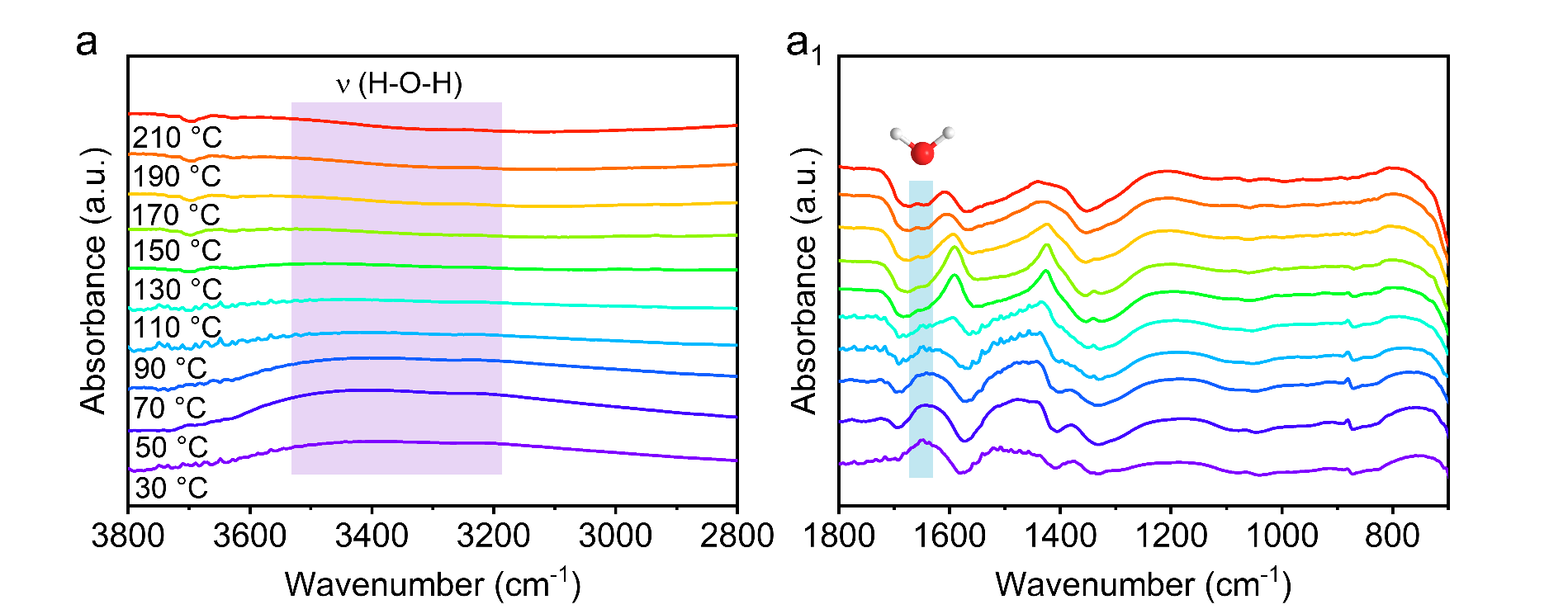


Figure S25. *In situ* DRIFTS of ethyl acetate oxidation in 5% H_2_O balanced with O_2_/N_2_ (a and a_1_) from 50 to 210 ℃ over MnCo_4_-E.


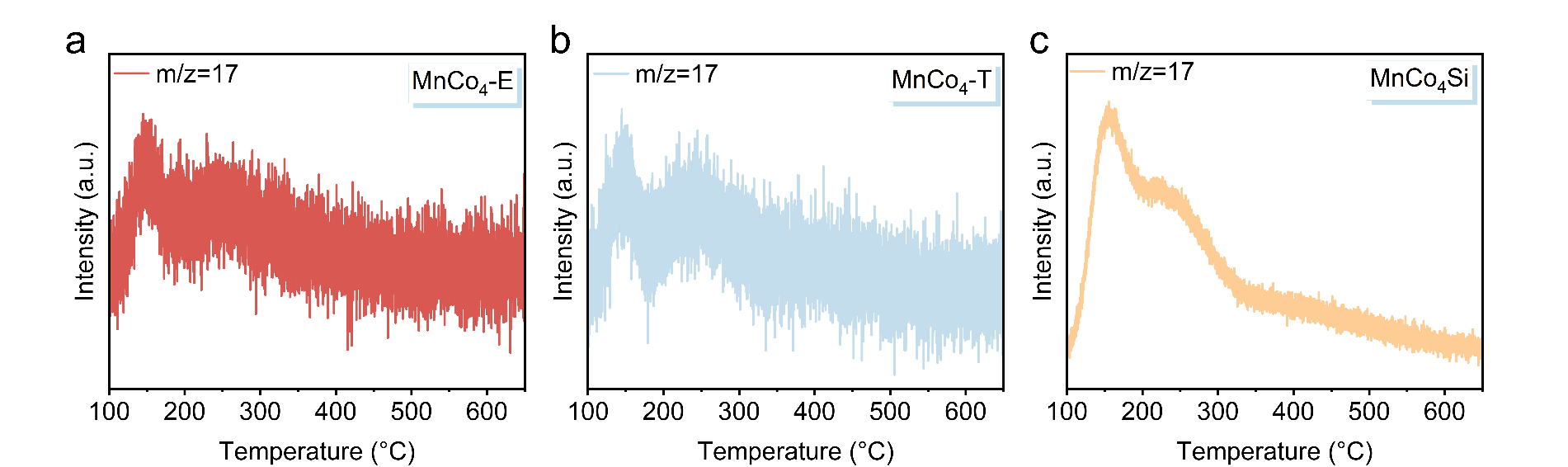


**Figure S26**. NH_3_ TPD-MS profiles of (a) MnCo_4_-E, (b) MnCo_4_-T, and (c) MnCo_4_Si.


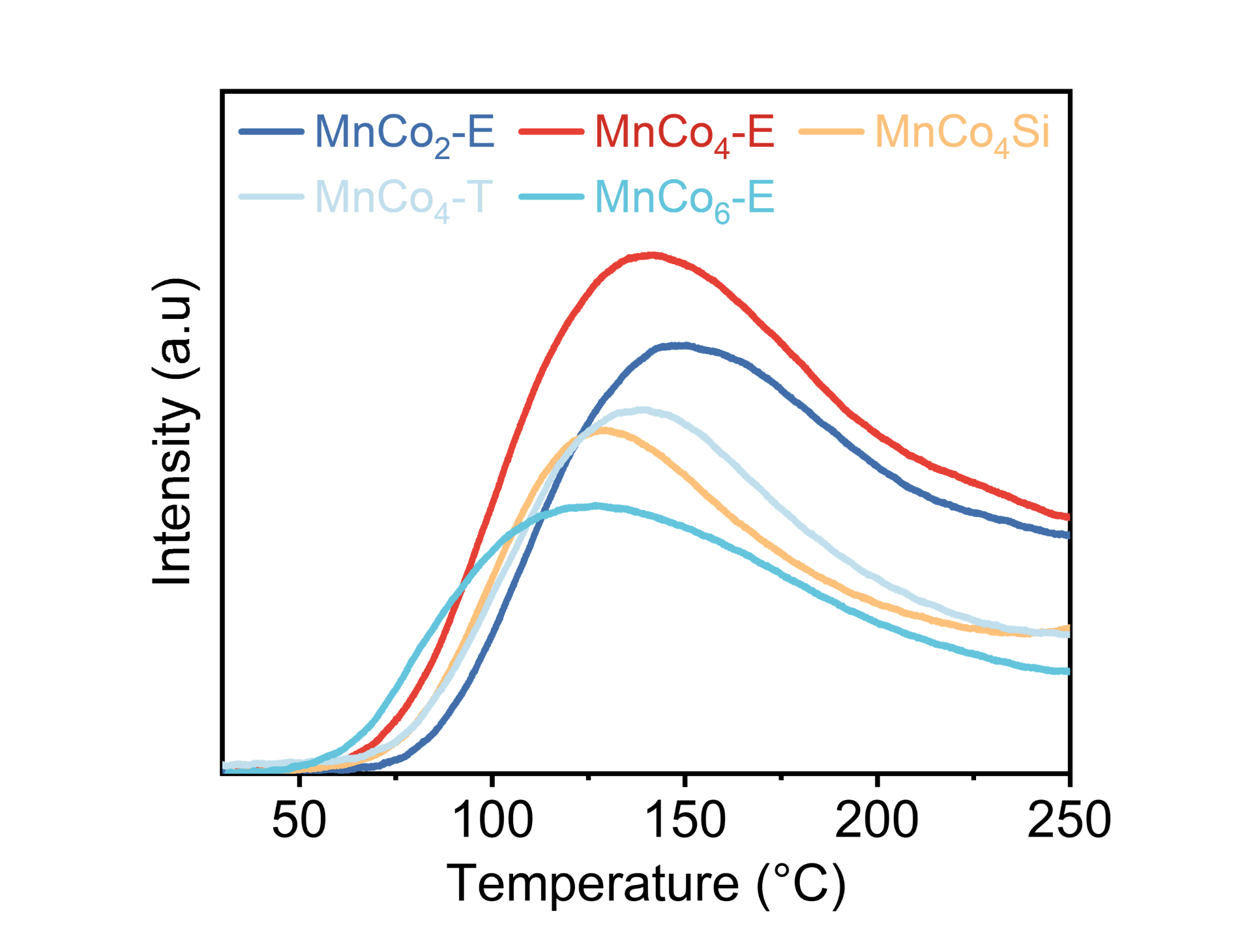


Figure S27. O_2_-TPD profiles of MnCo series catalysts from 30 to 250 °C.


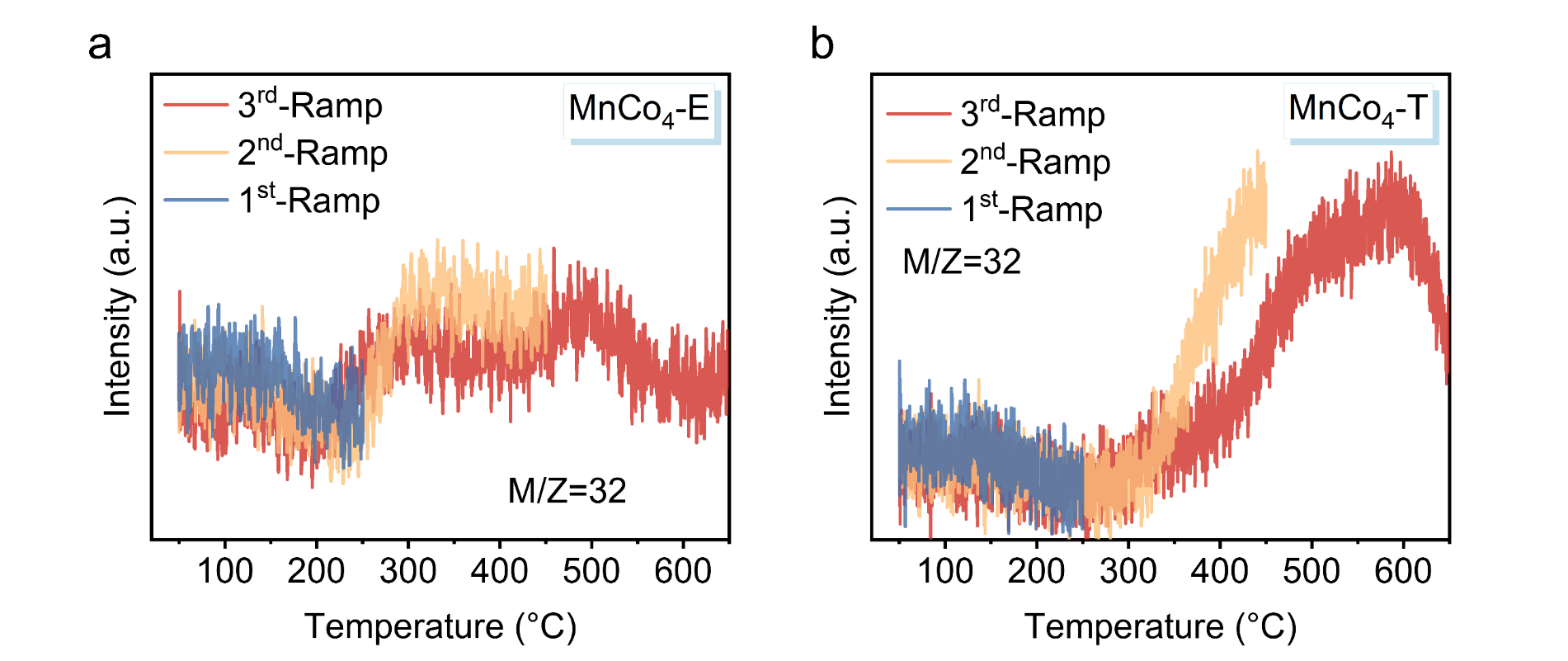


Figure S28. Cycling O_2_-TPD-MS of (a) MnCo_4_-E and (b) MnCo_4_-T.


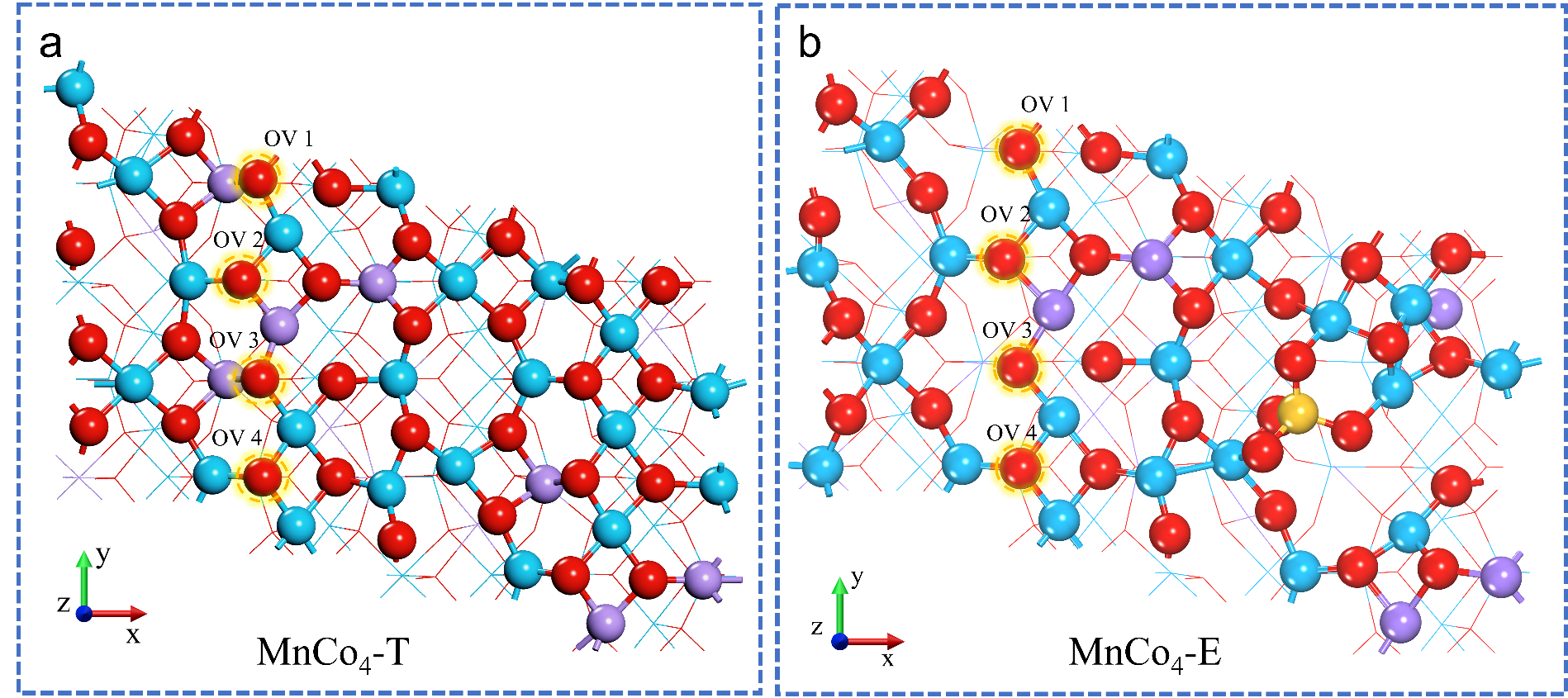


Figure S29. Top view of oxygen vacancy locations in (a) MnCo_4_-T and (b) MnCo_4_-E.


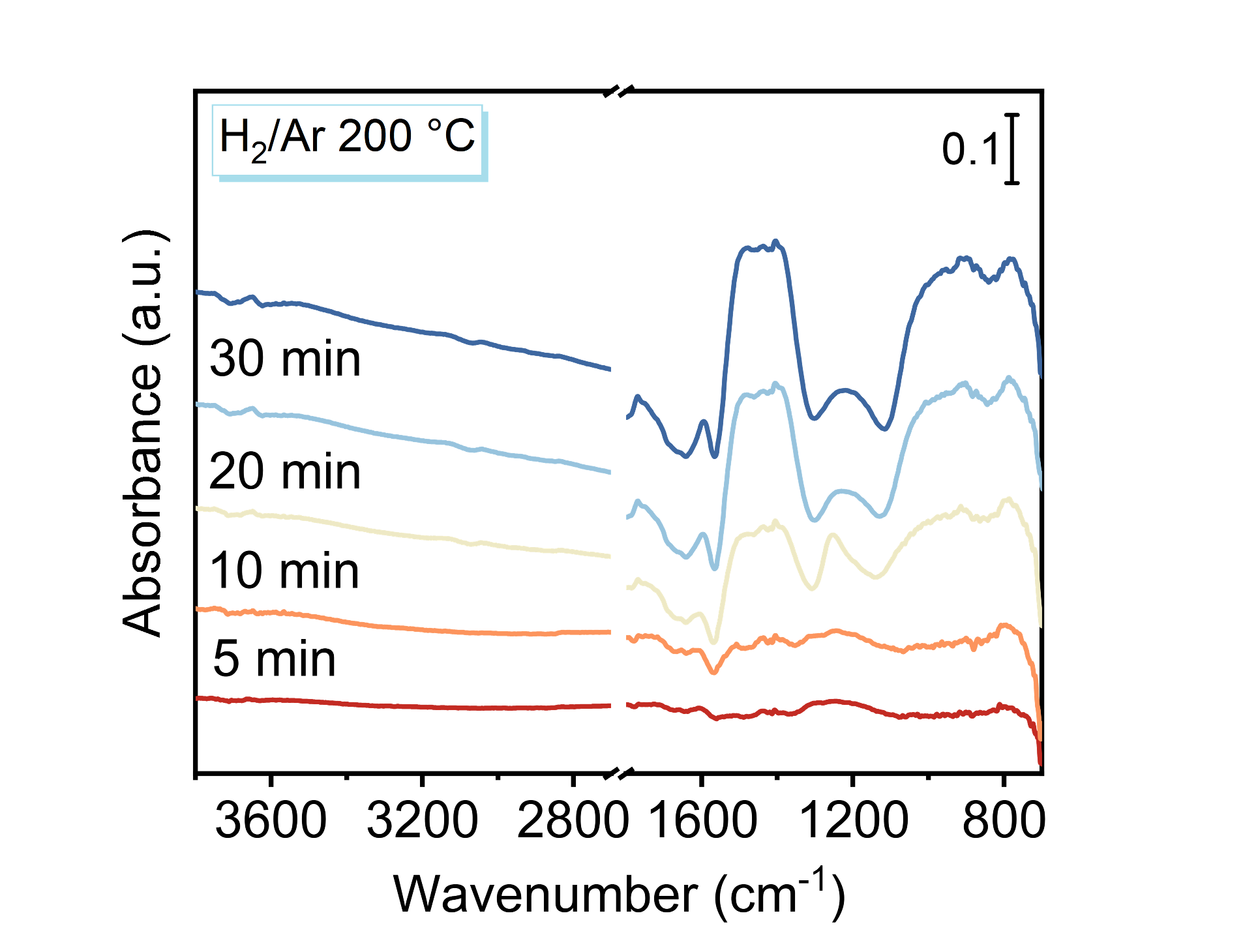


Figure S30. *In situ* DRIFTS of H_2_ oxidation by oxygen species at MnCo_4_-E surface in H_2_/Ar at 200 °C as a function of time.


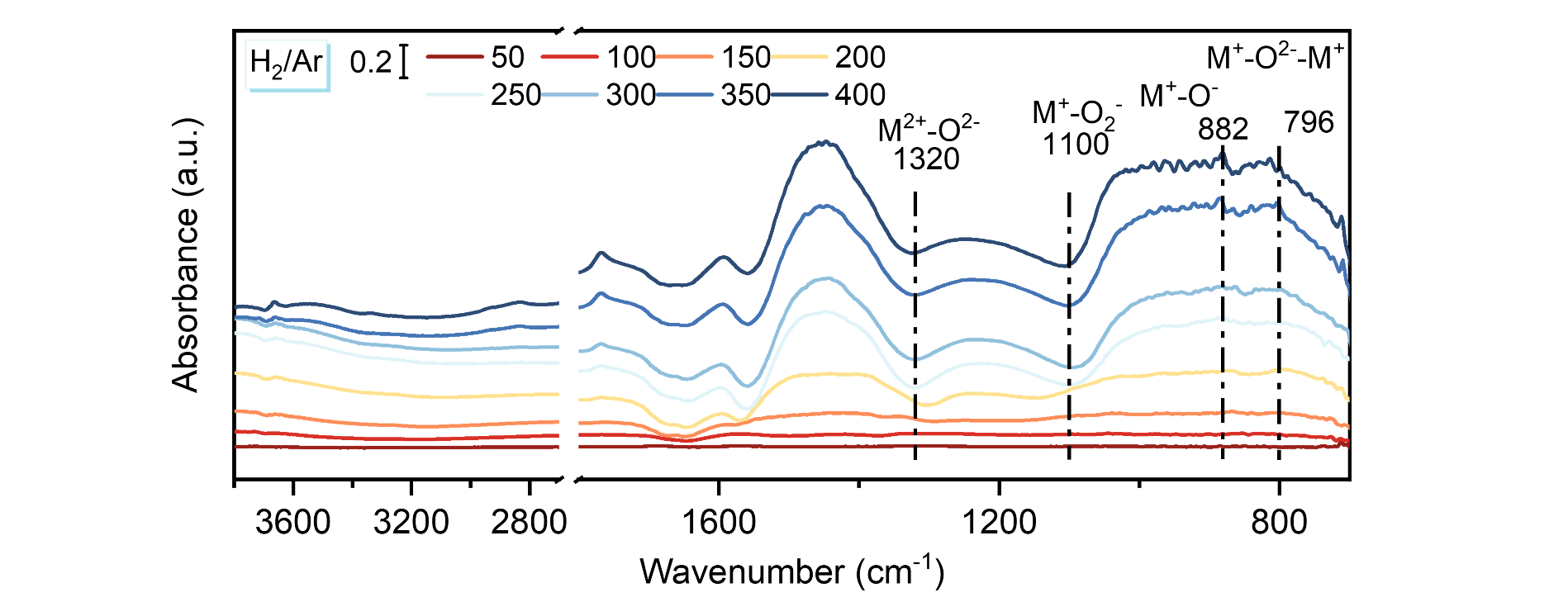


Figure S31. *In situ* DRIFTS of H_2_ oxidation by oxygen species on MnCo_4_-E surface in H_2_/Ar as a function of temperature.


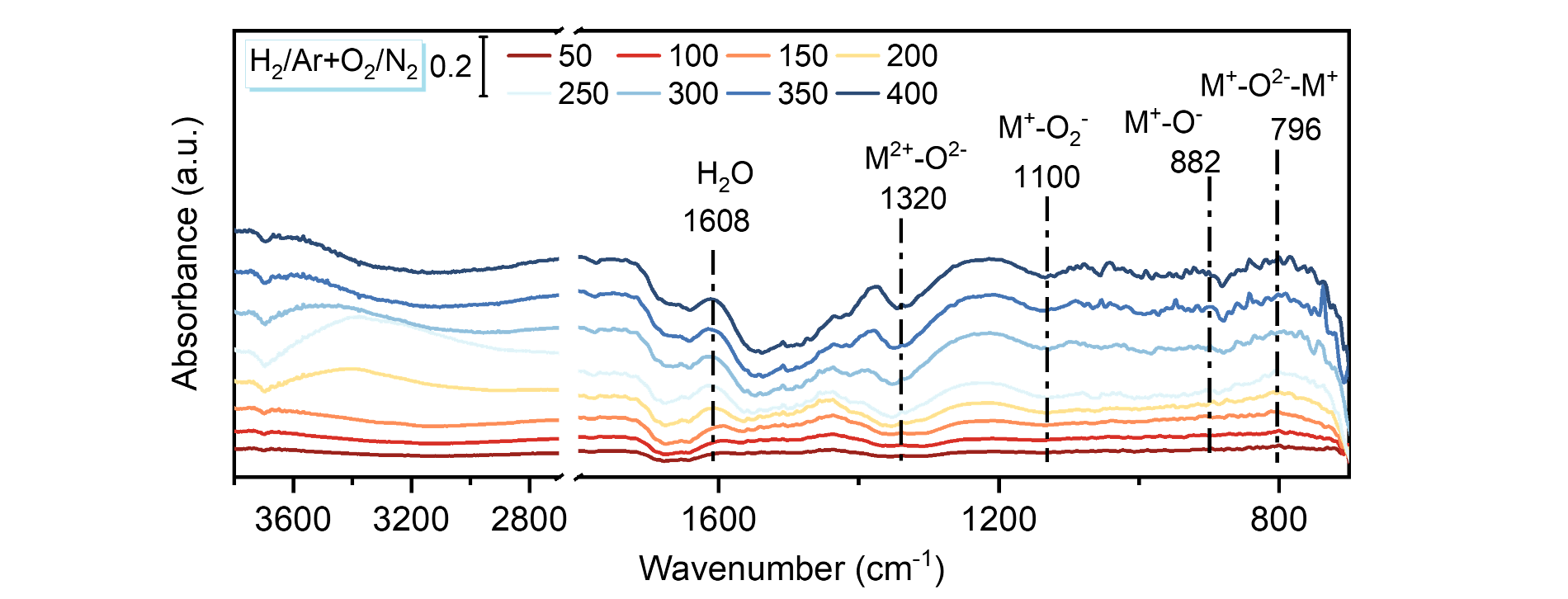


Figure S32. *In situ* DRIFTS of H_2_ oxidation by oxygen species on MnCo_4_-E surface in H_2_/Ar +O_2_/N_2_ as a function of temperature.


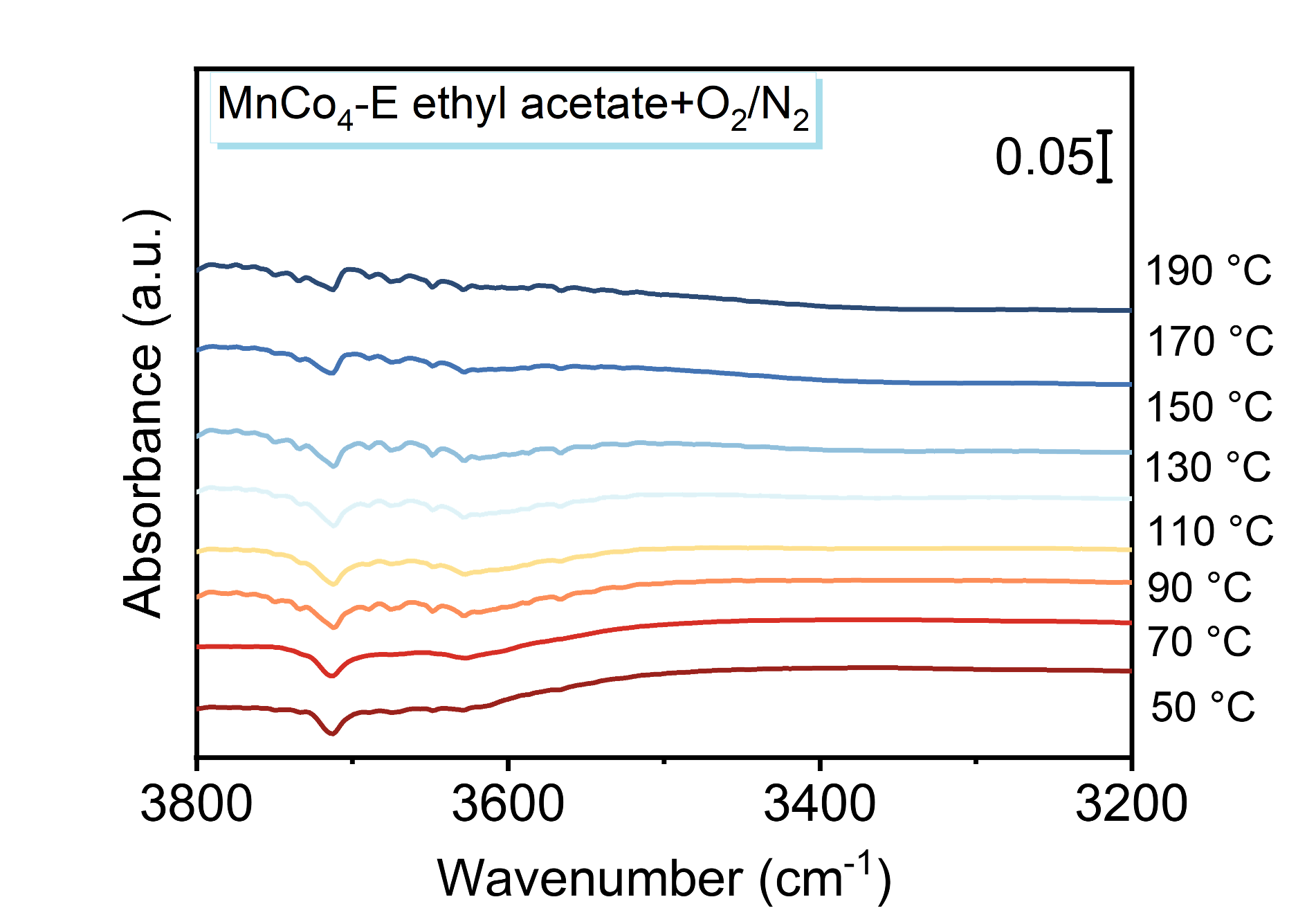


Figure S33. *In situ* DRIFTS of ethyl acetate oxidation in O_2_/N_2_ from 50 to 190 °C over MnCo_4_-E.


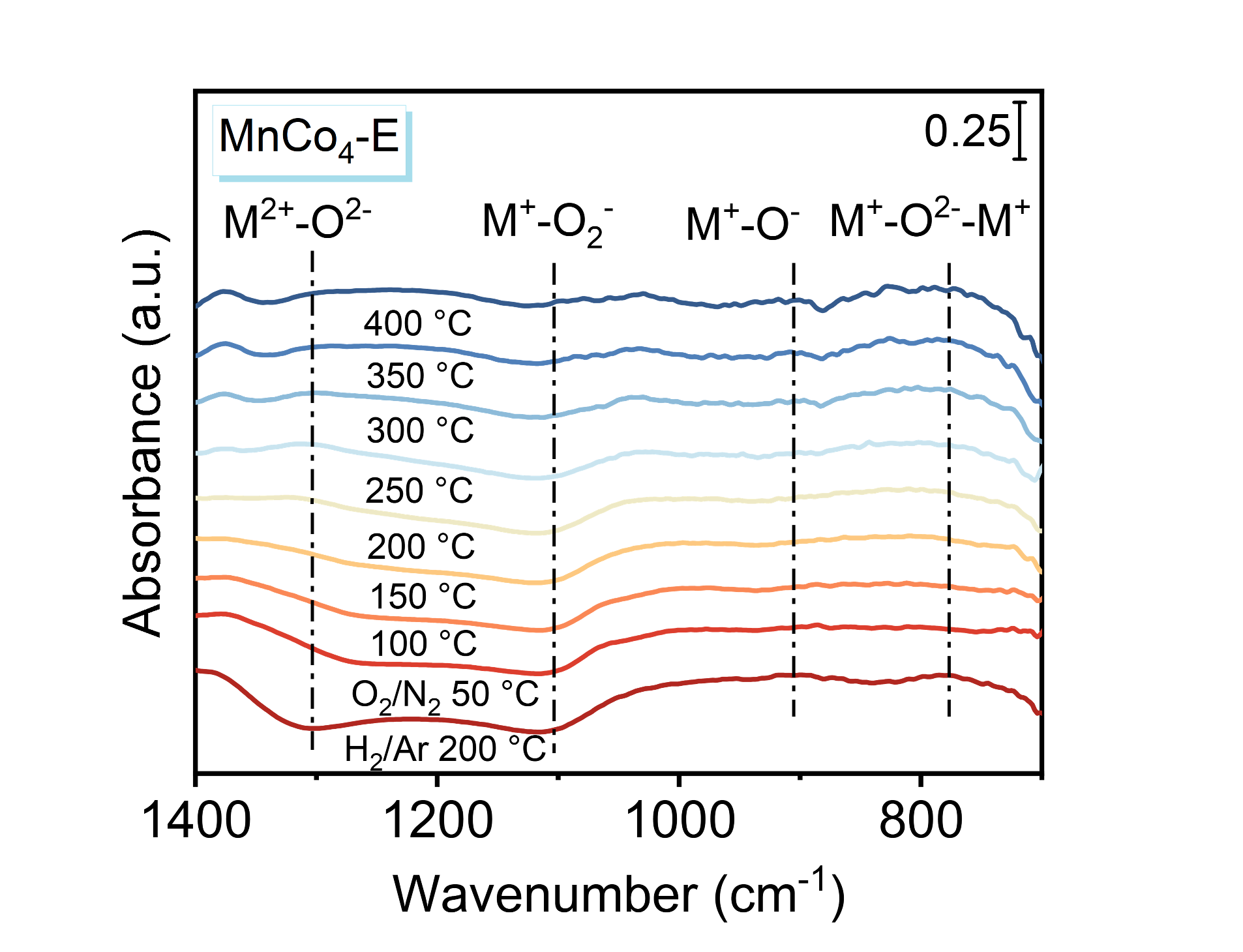


Figure S34. *In situ* DRIFTS of MnCo_4_-E catalyst replenishing oxygen vacancies in O_2_/N_2_ as a function of temperature.


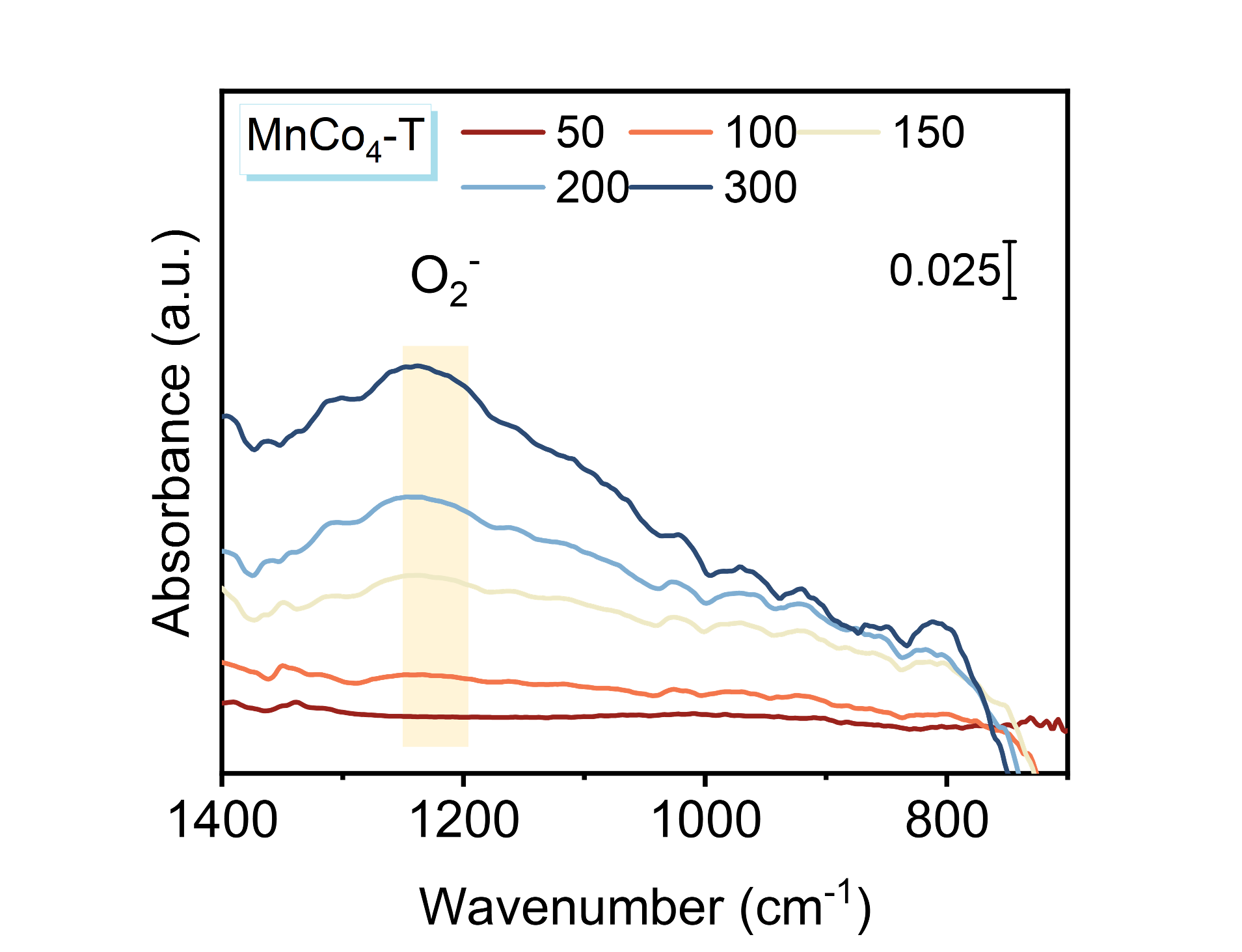


Figure S35. *In situ* DRIFTS of MnCo_4_-T exposed under the 20% O_2_/N_2_ as a function of temperature.


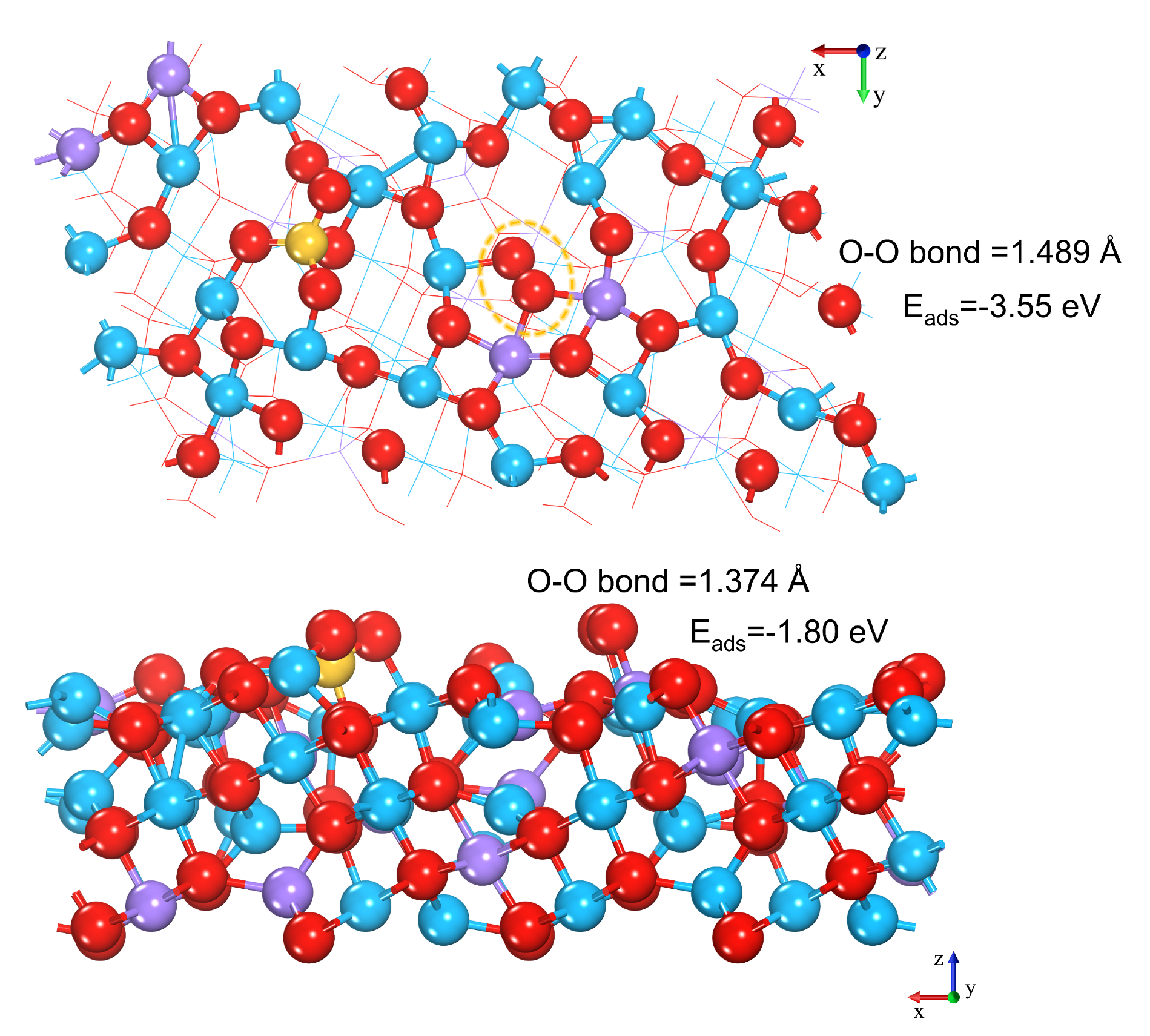


Figure S36. Optimized adsorption configurations of oxygen molecules on MnCo_4_-E catalysts.


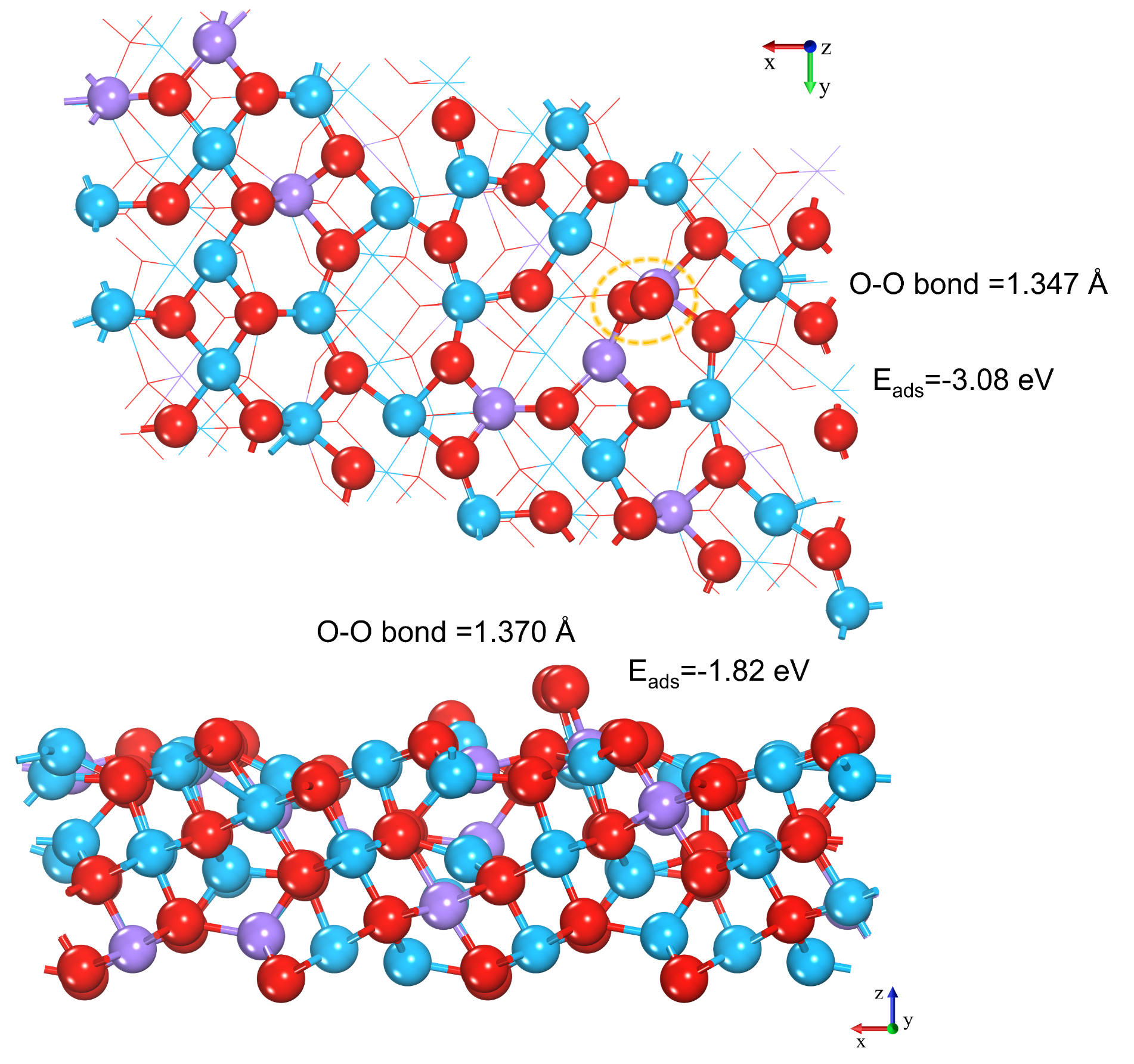


Figure S37. Optimized adsorption configurations of O_2_ molecules on MnCo_4_-T catalysts.


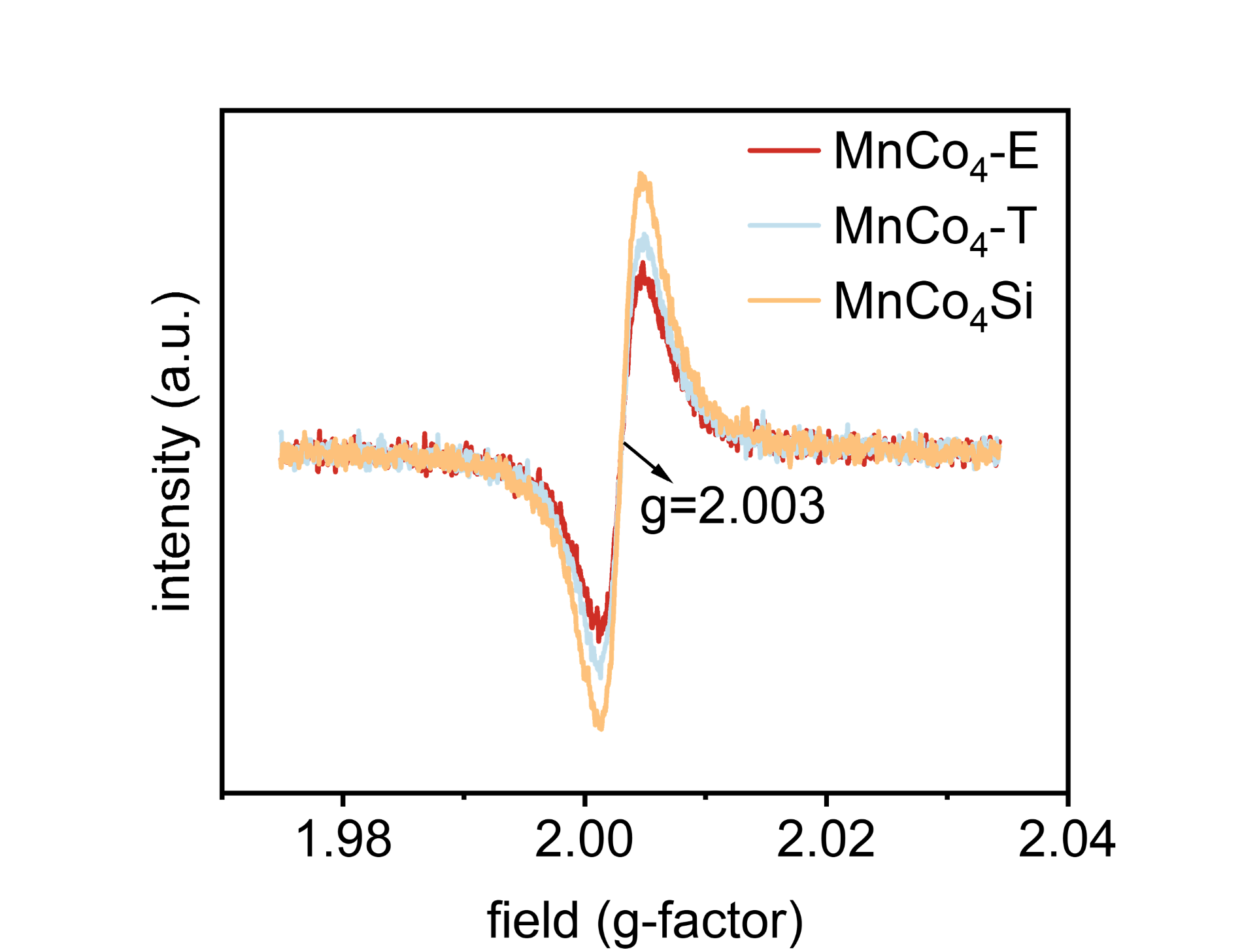


Figure S38. EPR profiles of MnCo_4_-E and related catalysts.


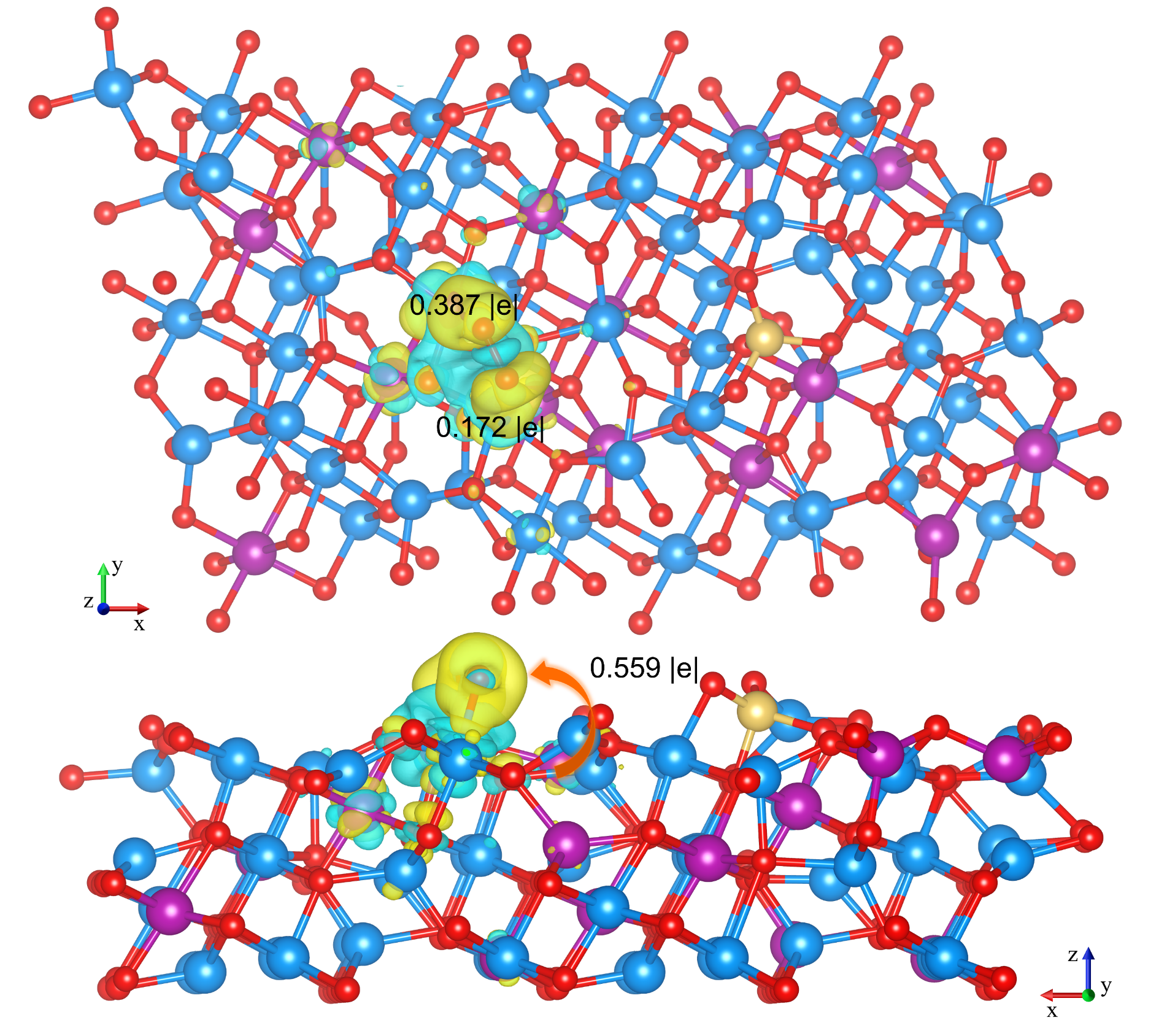


Figure S39. Bader charge variation of ethyl acetate for theoretical models of MnCo_4_-E catalysts.


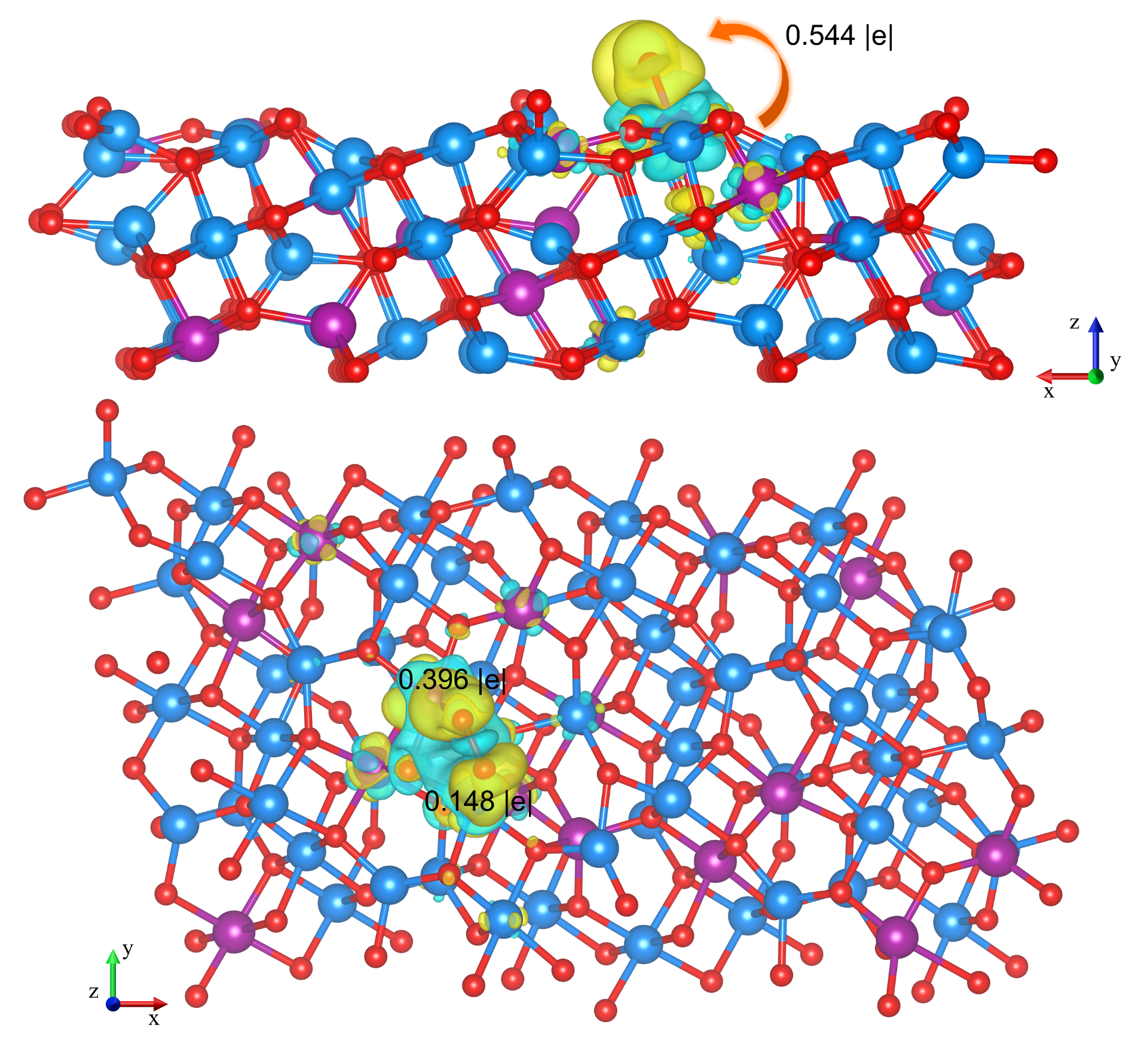


Figure S40. Bader charge variation of ethyl acetate for theoretical models of MnCo_4_-T catalysts.


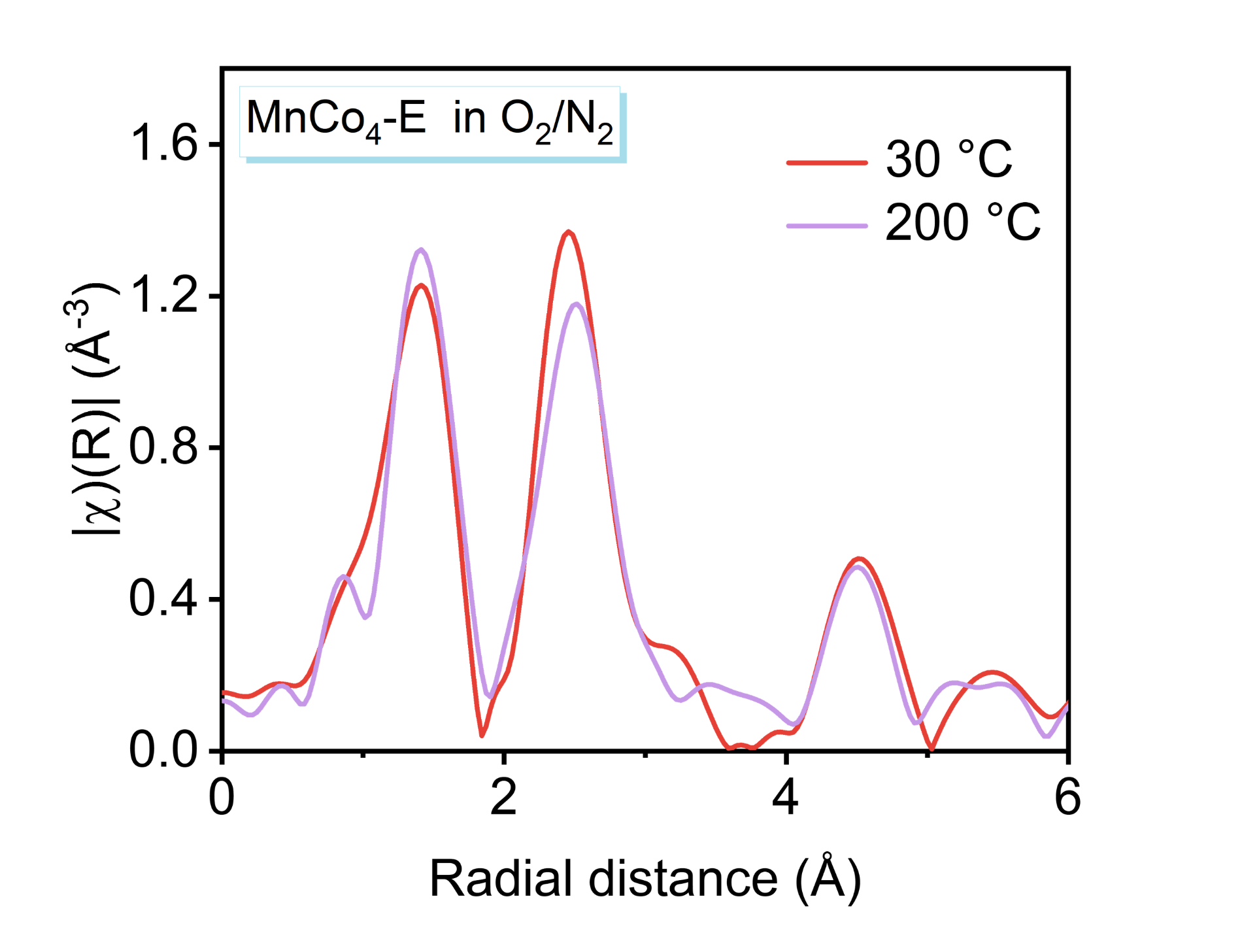


Figure S41. *In situ* EXAFS spectra of MnCo_4_-E under O_2_/N_2_ flow from 30 to 200 °C.


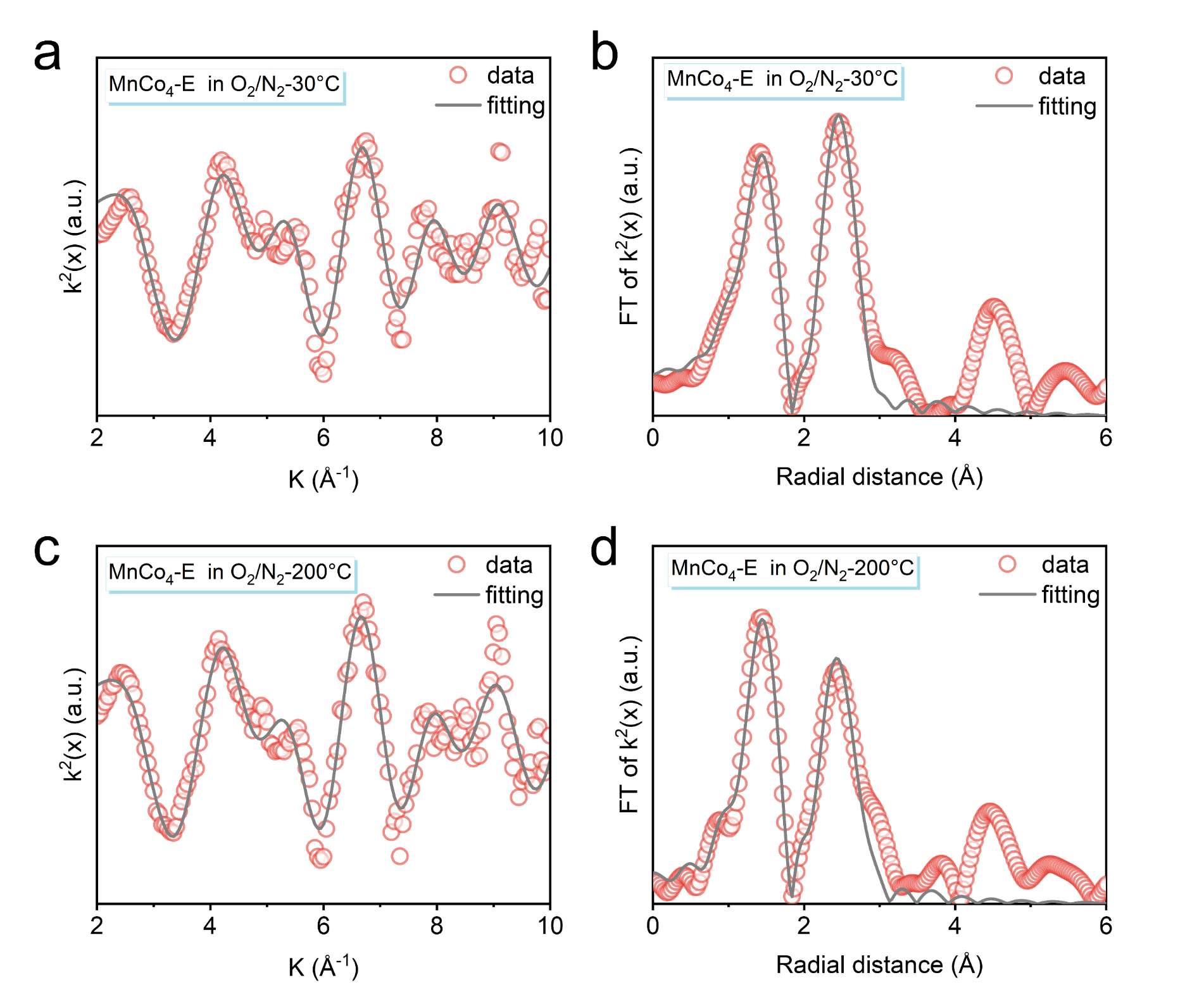


Figure S42. Mn K-edge EXAFS K and R space fitting curves of MnCo_4_-E under O_2_/N_2_ flow at (a-b) 50 °C, and (c-d) 100 °C.


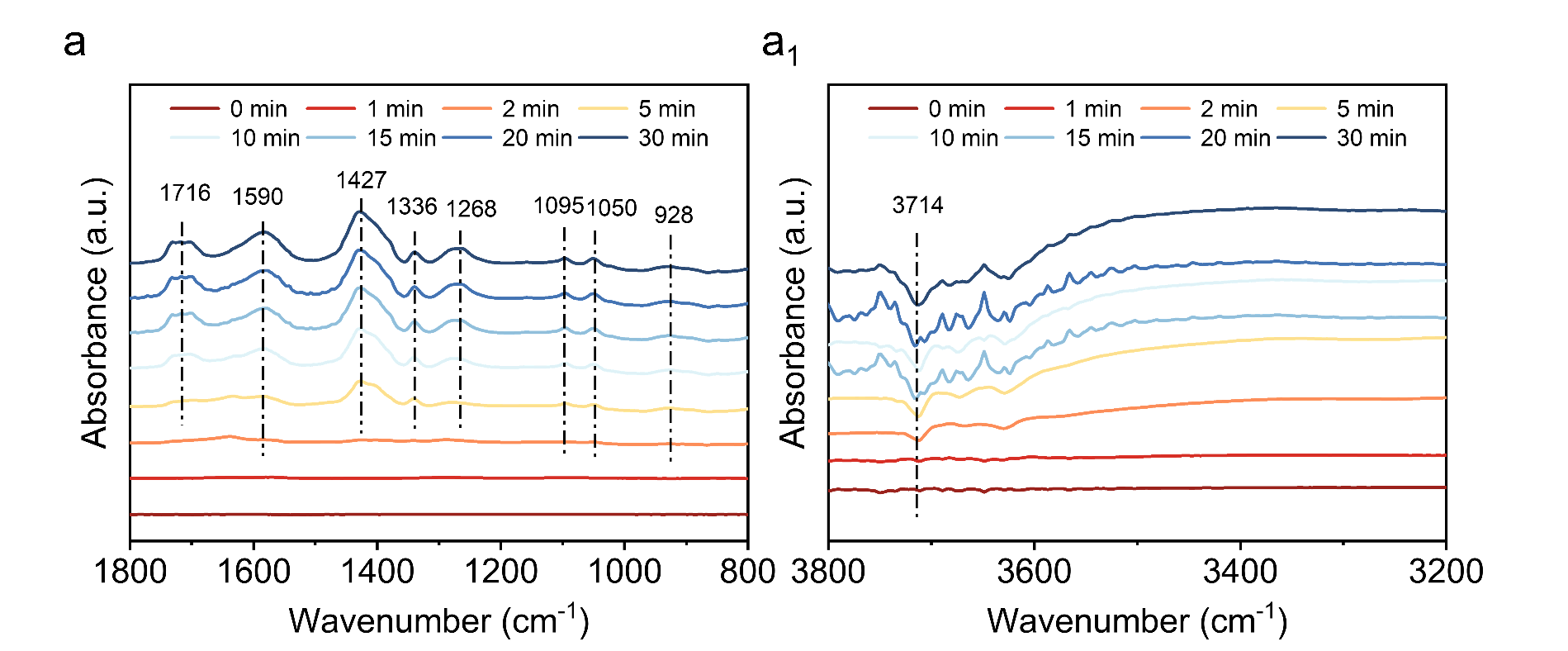


Figure S43. *In situ* DRIFTS of ethyl acetate adsorption on the MnCo_4_-E (a and a_1_) under 20% O_2_/N_2_ flow.


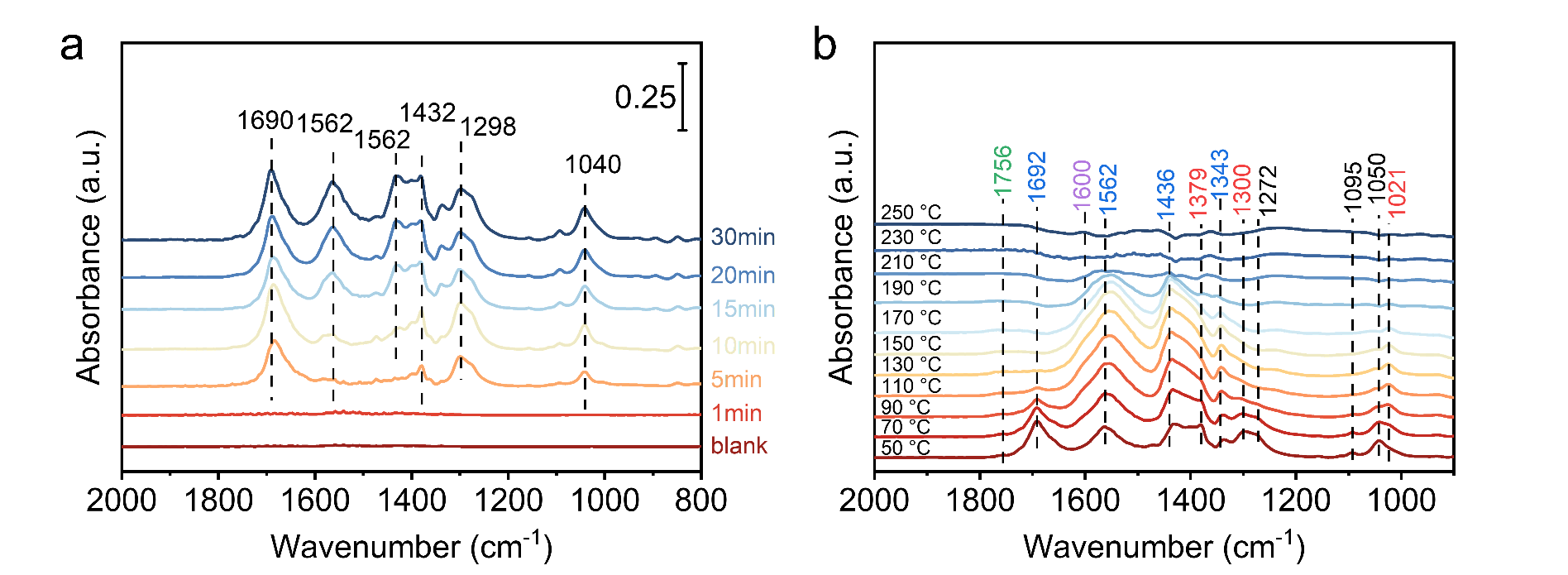


Figure S44. *In situ* DRIFTS of ethyl acetate (a) absorption in O_2_/N_2_ at 30 °C and (b) oxidation from 50 to 250 °C over MnCo_4_-T.


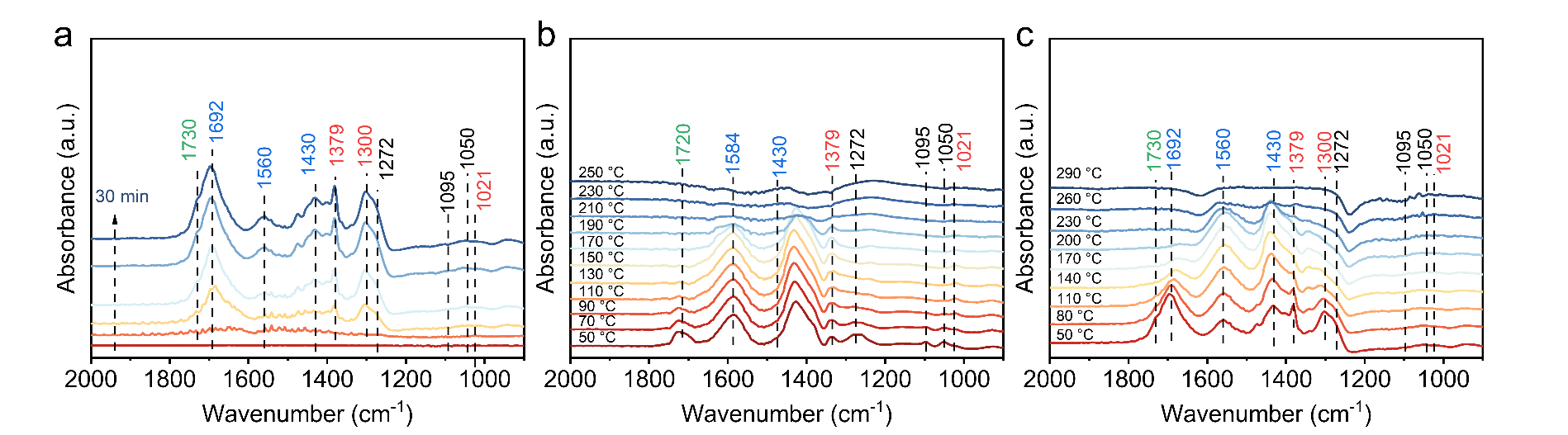


Figure S45. *In situ* DRIFTS of ethyl acetate adsorption over MnCo_4_Si from 0 to 30 min at 30 ℃ in (a) O_2_/N_2_ flow. *In situ* DRIFTS of ethyl acetate oxidation in (b) O_2_/N_2_, (c) N_2_ from 50 to 250 ℃ over MnCo_4_Si.


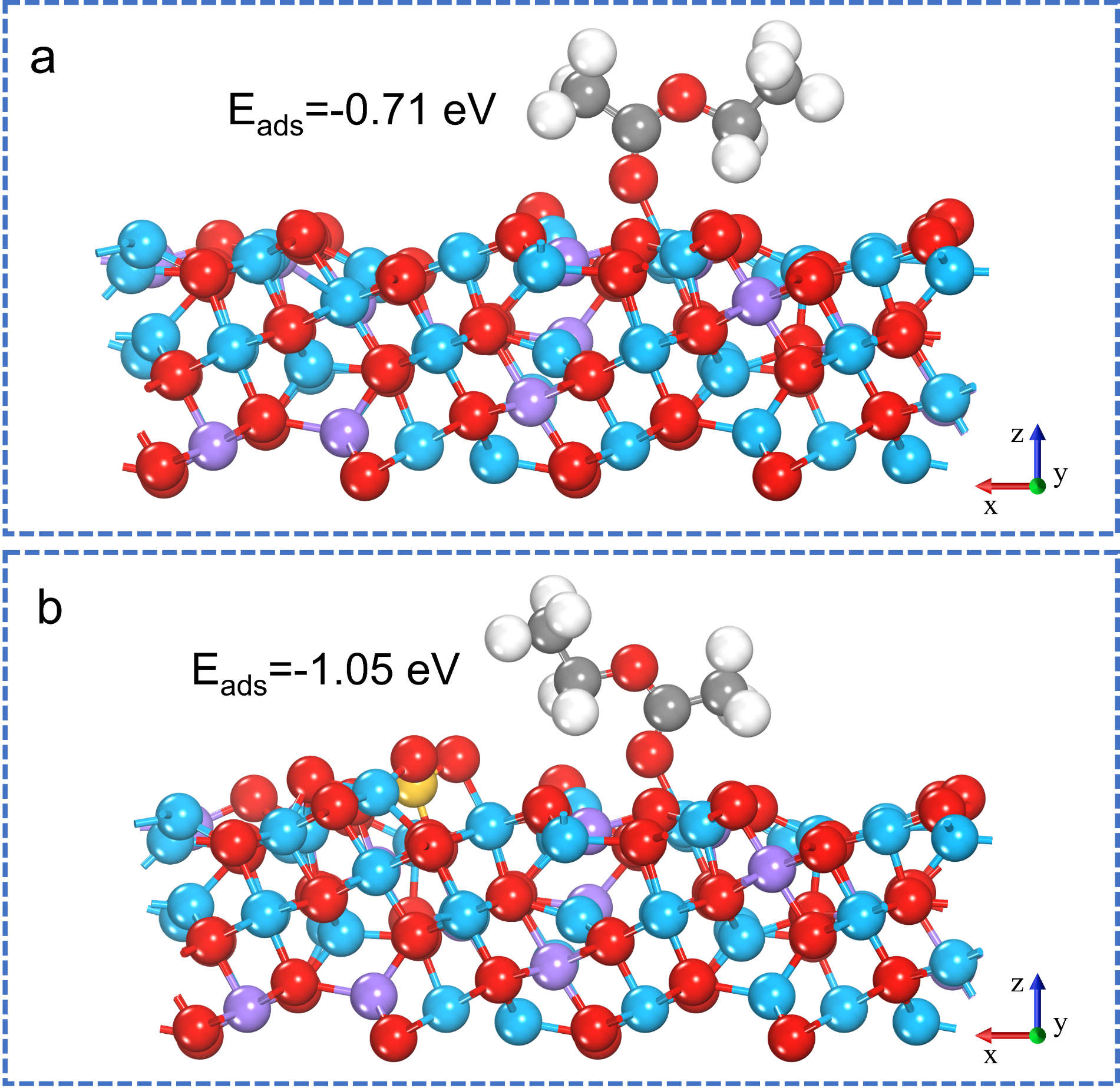


Figure S46. Optimized adsorption configurations of ethyl acetate on (a) MnCo_4_-T and (b) MnCo_4_-E catalysts.


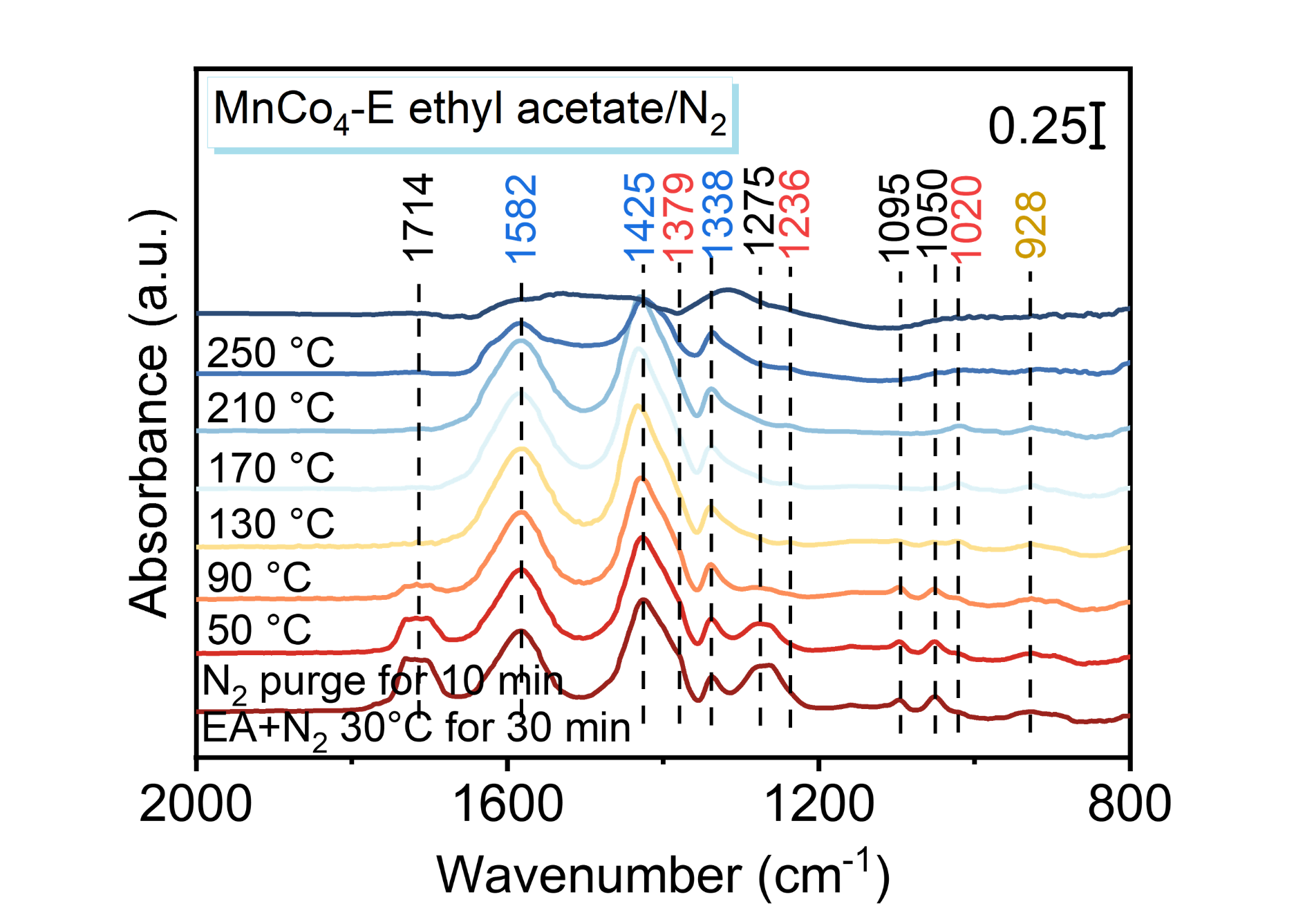


Figure S47. *In situ* DRIFTS of EA absorption in N_2_ at 30 °C and oxidation from 50 to 250 °C.


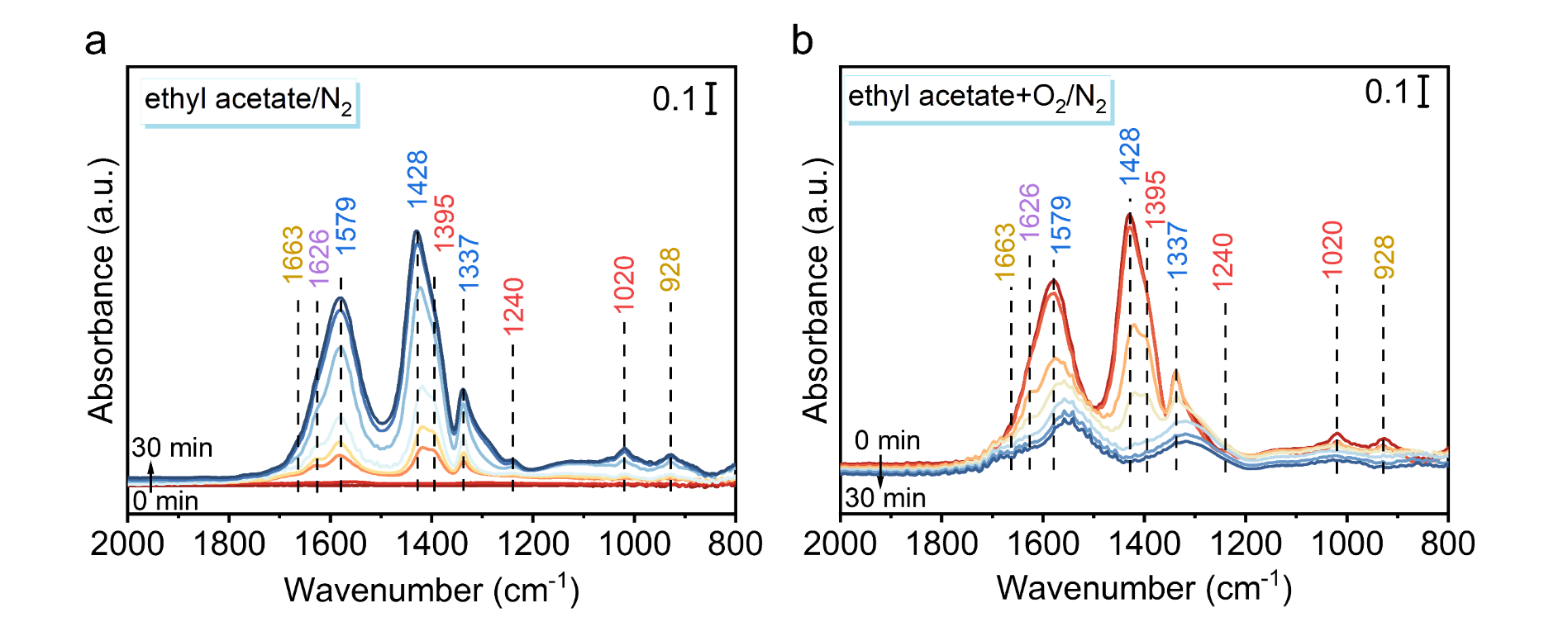


Figure S48. *In situ* DRIFTS of EA (a) absorption in N_2_ and (b) oxidation in O_2_/N_2_ as a function of time at 170 °C over MnCo_4_-E.


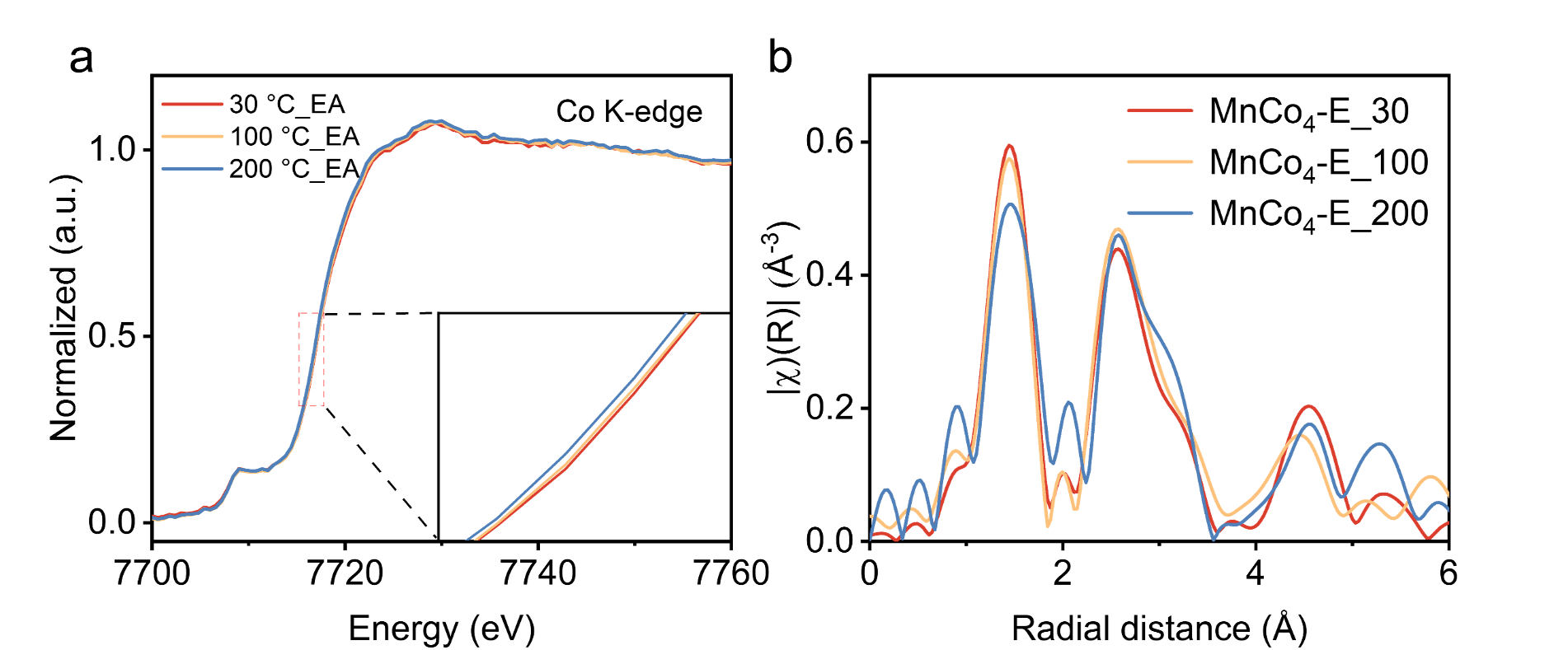


Figure S49. (a) Normalized Co K-edge *in situ* XANES spectra of MnCo_4_-E and (b) In-situ EXAFS spectra of the transient reaction with ethyl acetate from 30 to 200 °C.


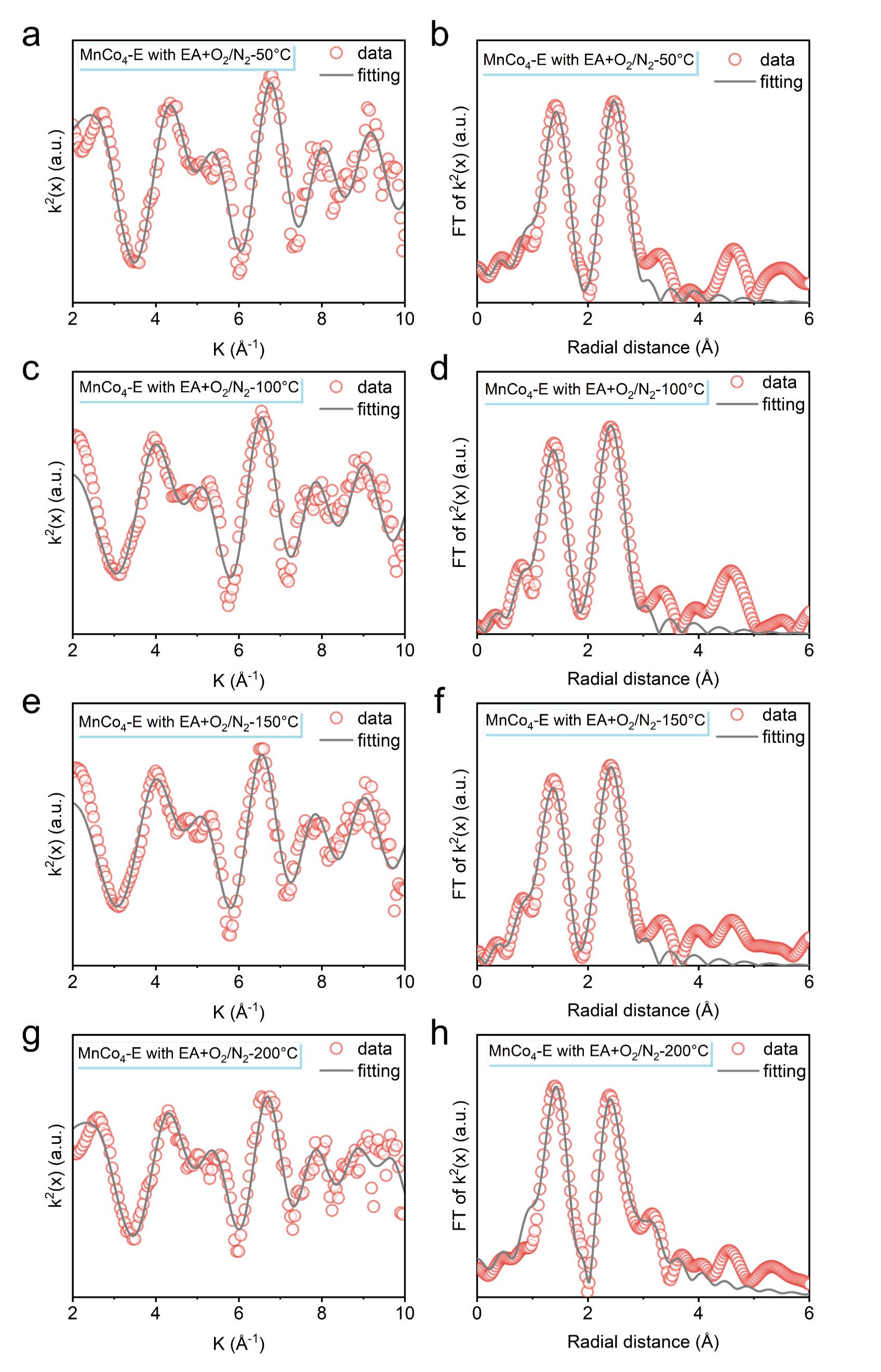


Figure S50. Mn K-edge EXAFS K and R space fitting curves of MnCo_4_-E under ethyl acetate flow balanced with O_2_/N_2_ at (a-b) 50 °C, (c-d) 100 °C, (e-f) 150 °C, and (g-h) 200 °C.

Supplementary Tables

Table S1. Physicochemical properties of catalysts measured by N_2_ sorption isotherms.

| **Sample** | **S_BET_ (m^2^ g^-1^)^a^** | **V _total_ (cm^3^ g^-1^)^b^** | **D_meso_ (nm)^b^** |
| --- | --- | --- | --- |
| MnCo_4_-E-10 | 61.4 | 0.40 | 10.56 |
| MnCo_4_-E-30 | 62.0 | 0.36 | 10.72 |
| MnCo_4_-E-60 | 62.8 | 0.40 | 10.33 |
| MnCo_4_-E-120 | 62.1 | 0.38 | 10.46 |
| MnCo_4_-E-360 | 57.0 | 0.36 | 8.75 |

^a^ Calculated using BET equation.

^b^ Calculated using BJH equation.

Table S2. Physicochemical properties of catalysts measured by N_2_ sorption isotherms.

| **Sample** | **S_BET_ (m^2^ g^-1^)^a^** | **V _total_ (cm^3^ g^-1^)^b^** | **D_meso_ (nm)^b^** |
| --- | --- | --- | --- |
| MnCo -E | 80.8 | 0.31 | 2.26 |
| MnCo_2_- E | 85.0 | 0.38 | 9.82 |
| MnCo_4_-E | 81.8 | 0.31 | 7.18 |
| MnCo_4_-T | 68.6 | 0.15 | 4.56 |
| MnCo_4_Si | 130.9 | 0.27 | 3.56 |

^a^ Calculated using BET equation.

^b^ Calculated using BJH equation.

Table S3. The Element content of MnCo_4_-E and related catalysts.

| **Sample** | **Mn content ^a^** | **Co**  **Content ^a^** | **Si**  **content ^a^** | **Si**  **content ^b^** | **Si**  **content ^c^** |
| --- | --- | --- | --- | --- | --- |
| MnCo_4_-E | 15.16 | 55.18 | 0.30 | 1.23 | 1.02 |
| MnCo_4_-T | 16.83 | 56.34 | **—** | / | / |
| MnCo_4_Si | 14.6 | 49.22 | 8.53 | 8.09 | / |

^a^ Determined by ICP-OES.

^b^ Obtained by EDS elemental mapping.

^c^ Determined by XRF.

Table S4. The ICP-OES result of MnCo_4_-E with different alkali-treated time.

| **Sample** | **Mn content** | **Co Content** | **Si content** |
| --- | --- | --- | --- |
| MnCo_4_-E-10 | 17.43 | 59.76 | 0.66 |
| MnCo_4_-E-30 | 16.72 | 57.33 | 0.76 |
| MnCo_4_-E-60 | 16.81 | 57.98 | 0.63 |
| MnCo_4_-E-120 | 16.67 | 57.79 | 0.48 |
| MnCo_4_-E-360 | 16.58 | 57.43 | 0.42 |

Table S5. XPS semi-quantitative analysis data of MnCo_4_-T and reference catalysts.

| **Sample** | **Co^3+^ ratio (%)** | **Mn^4+^ ratio (%)** | **O_ads_ ratio (%)** |
| --- | --- | --- | --- |
| MnCo_4_-T | 42 | 34 | 22 |
| MnCo_4_Si | 41 | 43 | 29 |
| MnCo_4_-E | 56 | 48 | 48 |

Table S6. H_2_ consumption of MnCo_4_-E and related catalysts.

| **Sample** | **Temperature (°C)** | | | | **H_2_ consumption**  **(mmol g^-1^)** | | | | **Total**  **(mmol g^-1^)** |
| --- | --- | --- | --- | --- | --- | --- | --- | --- | --- |
|  | Peak 1 | Peak 2 | Peak 3 | Peak 4 | Peak 1 | Peak 2 | Peak 3 | Peak 4 |  |
| MnCo_2_-E | 265 | 348 | 495 | 611 | 0.87 | 2.51 | 2.47 | 4.06 | 9.92 |
| MnCo_4_-E | 256 | 337 | 450 | 521 | 0.69 | 2.62 | 2.14 | 5.80 | 11.24 |
| MnCo_6_-E | 242 | 356 | 453 | 503 | 0.48 | 2.45 | 1.73 | 6.91 | 11.58 |
| MnCo_4_Si | 245 | 342 | 490 | 598 | 0.42 | 2.22 | 2.62 | 4.15 | 9.41 |
| MnCo_4_-T | 161 | 218 | 298 | 405 | 0.20 | 0.40 | 2.85 | 7.72 | 11.17 |

Table S7. O_2_ desorption on MnCo_4_-E and related catalysts.

| **Sample** | **Temperature (°C)** | | **O_2_ desorption**  **(mmol g^-1^)** | | **Total**  **(mmol g^-1^)** |
| --- | --- | --- | --- | --- | --- |
|  | **O_α_** | **O_β_** | **O_α_** | **O_β_** |  |
| MnCo_2_-E | 146 | 302 | 0.14 | 0.10 | 0.25 |
| MnCo_4_-E | 138 | 325 | 0.18 | 0.21 | 0.39 |
| MnCo_6_-E | 141 | 384 | 0.11 | 0.11 | 0.22 |
| MnCo_4_Si | 128 | 357 | 0.08 | 0.16 | 0.24 |
| MnCo_4_-T | 140 | 327 | 0.11 | 0.11 | 0.11 |

Table S8. Structural parameters of MnCo_4_-E, MnCo_4_Si, and MnCo_4_-T extracted from the EXAFS fitting.

| **Scattering pair** | | **CN** | **R (Å)** | **σ^2^ (10^-3^ Å)** | **ΔE_0_ (eV)** | **R factor** |
| --- | --- | --- | --- | --- | --- | --- |
| MnCo_4_-E | Mn-O | 4.5$\text{±}$0.6 | 1.93 | 6.3$\text{±}$1.9 | -5.4$\text{±}$0.5 | 0.010 |
|  | Mn-Co | 8.5$\text{±}$1.5 | 2.89 | 10.7$\text{±}$1.7 |  |  |
| MnCo_4_Si | Mn-O | 3.9$\text{±}$0.9 | 1.91 | 4.1$\text{±}$2.4 | -8.0$\text{±}$1.4 | 0.020 |
|  | Mn-Co | 7.6$\text{±}$2.1 | 2.89 | 10.8$\text{±}$2.6 |  |  |
| MnCo_4_-T | Mn-O | 4.4$\text{±}$0.9 | 1.91 | 5.4$\text{±}$2.4 | -8.4$\text{±}$0.9 | 0.019 |
|  | Mn-Co | 6.1$\text{±}$1.6 | 2.87 | 9.4$\text{±}$2.5 |  |  |

Table S9. Structural parameters of MnCo_4_-E, MnCo_4_Si, and MnCo_4_-T extracted from the EXAFS fitting.

| **Scattering pair** | | **CN** | **R (Å)** | **σ^2^ (10^-3^ Å)** | **ΔE_0_ (eV)** | **R factor** |
| --- | --- | --- | --- | --- | --- | --- |
| MnCo_4_-E | Co-O | 4.4$\text{±}$0.2 | 1.94 | 3.0 | -1.9$\text{±}$1.2 | 0.005 |
|  | Co-Mn | 3.3$\text{±}$0.3 | 2.85 | 3.0 | -8.4$\text{±}$2.9 |  |
|  | Co-Co | 10.5$\text{±}$3.3 | 3.36 | 9.2$\text{±}$2.7 | -8.3$\text{±}$1.6 |  |
| MnCo_4_Si | Co-O | 4.5$\text{±}$0.3 | 1.94 | 3.0 | -2.8$\text{±}$0.3 | 0.007 |
|  | Co-Mn | 3.2$\text{±}$0.4 | 2.85 | 3.0 | -9.8$\text{±}$3.3 |  |
|  | Co-Co | 12.9$\text{±}$4.3 | 3.37 | 10.9$\text{±}$3.1 | -8.5$\text{±}$1.8 |  |
| MnCo_4_-T | Co-O | 4.5$\text{±}$0.2 | 1.90 | 3.0 | 2.6$\text{±}$0.9 | 0.005 |
|  | Co-Mn | 2.8$\text{±}$0.3 | 2.84 | 3.0 | -0.7$\text{±}$1.7 |  |
|  | Co-Co | 12.0$\text{±}$3.0 | 3.33 | 11.2 | -0.7$\text{±}$1.7 |  |

Table S10. The parameters of Weisz-Prater criterion (*C_WP_*) for internal diffusion of all catalysts.

| Catalyst | ρ_c_  (kg m^-3^) | C_s_  (mol m^-3^) | Rate ×10^8^  (mol g_cat._ ^-1^ s^-1^) | D_eff_ ×10^6^  (m^2^ s^-1^) | C_WP_ | C*_WP_*  Compare to 1 |
| --- | --- | --- | --- | --- | --- | --- |
| MnCo_4_-E | 566 | 0.089 | 2.9 | 8.9 | 0.00186 | <1 |
| MnCo_4_-T | 707 | 0.089 | 2.8 | 8.9 | 0.00221 | <1 |
| MnCo_4_Si | 472 | 0.089 | 3.3 | 8.9 | 0.00176 | <1 |

Table S11. The catalytic activities of ethyl acetate oxidation over reported catalysts.

| **Catalyst** | **EA concentration (ppm)** | **WHSV**  **(mL g^-1^ h^-1^)** | **T_90_**  **(°C)** | **Ref.** |
| --- | --- | --- | --- | --- |
| MnO_2_/CoAlO-P | 1000 | 48,000 | 193 | (7) |
| 5% Mn-CeO_2_ | 1000 | 30,000 | 189 | (8) |
| CoMn-H1-T1 | 800 | 30,000 | 180 | (9) |
| α-MnO_2_ | 1000 | 78,000 | 176 | (10) |
| MnOx-NA | 1100 | 18,600 | 194 | (11) |
| 1Ru-5Cu/TiO_2_ | 500 | 60,000 | 208 | (12) |
| 5-MI-500 | 1000 | 60,000 | ~190 | (13) |
| N-Co_3_O_4_-C | 1000 | 30,000 | 177 | (14) |
| MnO_x_-CeO_2_-s | 500 | 60,000 | 205 | (15) |
| CoCe_0.75_Zr_0.25_-NF | 1000 | 60,000 | 227 | (16) |
| CuCe_0.75_Zr_0.25_/Z | — | 24,000 | 248 | (17) |
| Mn_2_O_3_-Cl | 1000 | 78,000 | 206 | (18) |
| MnCo_4_-E | 1000 | 60,000 | 178 | This work |
| MnCo_4_-E | 1000 | 30,000 | 166 | This work |

Table S12. Structural parameters of MnCo_4_-E in O_2_/N_2_ flow at different temperatures extracted from the EXAFS fitting.

| **Scattering pair** | | **CN** | **R (Å)** | **σ^2^ (10^-3^ Å)** | **ΔE_0_ (eV)** | **R factor** |
| --- | --- | --- | --- | --- | --- | --- |
| 30 ℃ | Mn-O | 4.3$\text{±}$0.7 | 1.91 | 5.3$\text{±}$2.5 | 0.4$\text{±}$1.1 | 0.011 |
|  | Mn-Co | 8.1$\text{±}$1.8 | 2.88 | 9.6$\text{±}$2.2 |  |  |
| 200 ℃ | Mn-O | 4.6$\text{±}$0.7 | 1.92 | 2.1$\text{±}$2.1 | -0.2$\text{±}$1.1 | 0.015 |
|  | Mn-Co | 8.6$\text{±}$2.4 | 2.89 | 9.4$\text{±}$2.8 |  |  |

Table S13. Structural parameters of MnCo_4_-E in ethyl acetate flow at different temperatures extracted from the EXAFS fitting.

| **Scattering pair** | | **CN** | **R (Å)** | **σ^2^ (10^-3^ Å)** | **ΔE_0_ (eV)** | **R factor** |
| --- | --- | --- | --- | --- | --- | --- |
| 50 ℃ | Mn-O | 4.0$\text{±}$0.8 | 1.91 | 5.7$\text{±}$3.1 | 3.0$\text{±}$0.9 | 0.017 |
|  | Mn-Co | 6.2$\text{±}$1.7 | 2.87 | 9.2$\text{±}$2.9 |  |  |
| 100 ℃ | Mn-O | 3.9$\text{±}$1.0 | 1.90 | 4.9$\text{±}$3.5 | -8.4$\text{±}$0.9 | 0.020 |
|  | Mn-Co | 6.1$\text{±}$1.7 | 2.87 | 8.5$\text{±}$2.9 |  |  |
| 150 ℃ | Mn-O | 3.9$\text{±}$1.1 | 1.90 | 6.5$\text{±}$4.2 | -8.3$\text{±}$1.2 | 0.017 |
|  | Mn-Co | 5.3$\text{±}$1.8 | 2.87 | 8.4$\text{±}$3.5 |  |  |
| 200 ℃ | Mn-O | 3.4$\text{±}$0.9 | 1.90 | 7.1$\text{±}$4.1 | 0.7$\text{±}$0.9 | 0.020 |
|  | Mn-Co | 4.5$\text{±}$2.1 | 2.87 | 8.5$\text{±}$3.8 |  |  |

Supplementary References

1. Kresse, G.; Furthmüller, J. “Efficiency of ab-Initio Total Energy Calculations for Metals and Semiconductors Using a Plane-Wave Basis Set.” *Computational Materials Science* 6 (1996): 15-50.
2. Kresse, G.; Hafner, J. “Ab Initio Molecular-Dynamics Simulation of the Liquid-Metal-Amorphous-Semiconductor Transition in Germanium.” *Physical Review B* 49 (1994): 14251.
3. Kresse, G.; Hafner, J. “Ab Initio Molecular Dynamics for Liquid Metals.” *Physical Review B* 47 (1993): 558.
4. Kresse, G.; Furthmüller, J. “Efficient Iterative Schemes for ab Initio Total-Energy Calculations Using a Plane-Wave Basis Set.” *Physical Review B* 54 (1996): 11169.
5. (29) Blöchl, P. E. “Projector Augmented-Wave Method.” *Physical Review B* 50 (1994): 17953.
6. Perdew, J. P.; Burke, K.; Ernzerhof, M. “Generalized Gradient Approximation Made Simple.” *Physical Review Letters* 77 (1996): 3865.
7. Zhao. Q, Zhang. Y, Liu. Q, et al. “Boosting the Catalytic Performance of Volatile Organic Compound Oxidation Over Platelike MnO_2_/CoAlO Catalyst by Weakening the Co–O Bond and Accelerating Oxygen Activation.” *ACS Catalysis* 13 (2023): 1492-1502.
8. Shen. Z, Gao. E, Meng. X, et al. “Mechanistic Insight into Catalytic Combustion of Ethyl Acetate on Modified CeO_2_ Nanobelts: Hydrolysis–Oxidation Process and Shielding Effect of Acetates/Alcoholates.” *Environmental Science Technology* 57 (2023): 3864-3874.
9. Shan. C, Zhang. Y, Zhao. Q, et al. “Acid Etching-Induced in Situ Growth of λ-MnO_2_ over CoMn Spinel for Low-Temperature Volatile Organic Compound Oxidation.” *Environmental Science Technology* 56 (2022):10381-10390.
10. Pan. T, Deng. H, Kang. S, et al. “Facile Homogeneous Precipitation Method to Prepare MnO_2_ with High Performance in Catalytic Oxidation of Ethyl Acetate.” *Chemical Engineering Journal* 417 (2021): 129246.
11. Zheng. Y, Liu. Q, Shan. C, et al. “Defective Ultrafine MnO_x_ Nanoparticles Confined Within a Carbon Matrix for Low-Temperature Oxidation of Volatile Organic Compounds.” *Environmental Science Technology* 55 (2021): 5403-5411.
12. Liu. X, Han. Q, Shi. W, et al. “Catalytic Oxidation of Ethyl Acetate Over Ru–Cu Bimetallic Catalysts: Further Insights into Reaction Mechanism via In Situ FTIR and DFT Studies.” *Journal of Catalysis* 369 (2019): 482-492.
13. Ye. Y, Xu. J, Gao. L, et al. “CuO/CeO_2_ Catalysts Prepared by Modified Impregnation Method for Ethyl Acetate Oxidation.” *Chemical Engineering Journal* 471 (2023): 144667.
14. Bi. F, Wei. J, Zhou. Z, et al. “Insight into the Synergistic Effect of Binary Nonmetallic Codoped Co_3_O_4_ Catalysts for Efficient Ethyl Acetate Degradation Under Humid Conditions.” *JACS Au* 5 (2025): 363-380.
15. Jiang. Y, Gao. J, Zhang. Q, et al. “Enhanced Oxygen Vacancies to Improve Ethyl Acetate Oxidation over MnO_x_-CeO_2_ Catalyst Derived from MOF Template.” *Chemical Engineering Journal* 371 (2019): 78-87.
16. Ma. M, Feng. X, Yang. R, et al. “Engineering CoCe_x_Zr_1−x_/Ni Foam Monolithic Catalysts for Ethyl Acetate Efficient Destruction.” *Fuel* 317 (2022): 123574.
17. Li. S, Hao. Q, Zhao. R, et al. “Highly Efficient Catalytic Removal of Ethyl Acetate Over Ce/Zr Promoted Copper/ZSM-5 Catalysts.” *Chemical Engineering Journal* 285 (2016): 536-543.
18. Zhang. Y, Wang. M, Kang. S, “Investigation of Suitable Precursors for Manganese Oxide Catalysts in Ethyl Acetate Oxidation.” *Journal of Environmental Sciences* 104 (2021): 17-26.
